# Structure-guided design of purine-based probes for selective Nek2 inhibition

**DOI:** 10.18632/oncotarget.13249

**Published:** 2016-11-09

**Authors:** Christopher R. Coxon, Christopher Wong, Richard Bayliss, Kathy Boxall, Katherine H. Carr, Andrew M. Fry, Ian R. Hardcastle, Christopher J. Matheson, David R. Newell, Mangaleswaran Sivaprakasam, Huw Thomas, David Turner, Sharon Yeoh, Lan Z. Wang, Roger J. Griffin, Bernard T. Golding, Céline Cano

**Affiliations:** ^1^ Northern Institute for Cancer Research, School of Chemistry, Newcastle University, Newcastle upon Tyne, UK; ^2^ Department of Molecular and Cell Biology, University of Leicester, Leicester, UK; ^3^ Cancer Research UK Cancer Therapeutics Unit, The Institute of Cancer Research, London, UK; ^4^ Northern Institute for Cancer Research, Newcastle University, Newcastle upon Tyne, UK

**Keywords:** cancer, Nek2, small molecule inhibitors, structure-guided design

## Abstract

Nek2 (NIMA-related kinase 2) is a cell cycle-dependent serine/threonine protein kinase that regulates centrosome separation at the onset of mitosis. Overexpression of Nek2 is common in human cancers and suppression can restrict tumor cell growth and promote apoptosis. Nek2 inhibition with small molecules, therefore, offers the prospect of a new therapy for cancer. To achieve this goal, a better understanding of the requirements for selective-inhibition of Nek2 is required. 6-Alkoxypurines were identified as ATP-competitive inhibitors of Nek2 and CDK2. Comparison with CDK2-inhibitor structures indicated that judicious modification of the 6-alkoxy and 2-arylamino substituents could achieve discrimination between Nek2 and CDK2. In this study, a library of 6-cyclohexylmethoxy-2-arylaminopurines bearing carboxamide, sulfonamide and urea substituents on the 2-arylamino ring was synthesized. Few of these compounds were selective for Nek2 over CDK2, with the best result being obtained for 3-((6-(cyclohexylmethoxy)-9*H*-purin-2-yl)amino)-*N,N*-dimethylbenzamide (CDK2 IC_50_ = 7.0 μM; Nek2 IC_50_ = 0.62 μM) with >10-fold selectivity. Deletion of the 6-substituent abrogated activity against both Nek2 and CDK2. Nine compounds containing an (*E*)-dialkylaminovinyl substituent at C-6, all showed selectivity for Nek2, *e.g*. (*E*)-6-(2-(azepan-1-yl)vinyl)-*N*-phenyl-9*H*-purin-2-amine (CDK2 IC_50_ = 2.70 μM; Nek2 IC_50_ = 0.27 μM). Structural biology of selected compounds enabled a partial rationalization of the observed structure activity relationships and mechanism of Nek2 activation. This showed that carboxamide 11 is the first reported inhibitor of Nek2 in the DFG-in conformation.

## INTRODUCTION

Abnormalities in centrosome number and function are common in many cancers, indicating that loss of centrosome cycle regulation may be a major factor in tumor progression [1]. Nek2 (NIMA-related kinase 2) is a human cell cycle-dependent serine/threonine protein kinase that localizes to the centrosome [2]. Nek2 is related to the fungal protein NIMA (never in mitosis gene A), an essential mediator of mitotic entry in *Aspergillus nidulans*. Like NIMA the activity of Nek2 peaks prior to mitotic entry, although in contrast to NIMA, Nek2 is not essential for mitotic entry in human cells. However, Nek2 does play a key role in ensuring timely assembly of the mitotic spindle, a scaffold that is vital for accurate segregation of sister chromatids during mitosis [3]. Nek2 interacts with centrosomal proteins that assemble into a filamentous linker that holds centrosomes in close proximity throughout interphase. Phosphorylation of these proteins by Nek2 in late G2 promotes linker disassembly and loss of centrosome cohesion; this in turn allows the timely separation of centrosomes in prophase [4]. In addition, Nek2 can promote cell cycle progression through phosphorylation of motor proteins that lead to disassembly of primary cilia [5]. Overexpression of Nek2 results in premature centrosome separation, which is a cause of chromosome segregation errors, aneuploidy and chromosomal instability, common genetic abnormalities observed in tumor cells. Upregulation of Nek2 expression has been observed in many human tumors, including ovarian [6], colorectal [7], prostatic [8], hepatocellular carcinoma [9] and breast cancer [10]. Nek2 depletion in a number of tumor cell lines causes growth suppression and apoptosis, while anti-tumor activity has been reported in a range of tumor cell lines following abrogation of Nek2 activity by RNAi depletion alone, or combined with cisplatin [11–14]. Hence, inhibiting Nek2 with small-molecule kinase inhibitors has potential as a novel cancer therapy.

Increasing efforts are now being applied to the development of Nek2 inhibitors with several small molecule inhibitors disclosed (Table 1). Examples include the aminopyrazine ATP-competitive inhibitor **1** (Nek2IC_50_ = 0.23 μM) [15] and a benzimidazole-based series with > 200-fold selectivity for Nek2 over Plk1 (*e.g*. **2**:Nek2 IC_50_ = 0.36 μM) [16]. To address the modest ligand efficiency (LE) of **2**, a hybrid class of compounds was generated by combining the core aminopyrazine moiety of the initial series with side-chains from the benzimidazole series. Optimisation of this new class of inhibitors improved potency, LE and kinase selectivity against a panel of cell cycle kinases, and culminated in the identification of **3** (Nek2 IC_50_ = 0.022 μM) [17]. A series of ATP-competitive reversible Nek2 inhibitors was also identified *via* high-throughput screening. These viridin/wortmannin-like compounds exhibited only modest Nek2-inhibitory activity (*e.g*. **4**:Nek2 IC_50_ = 1.9 μM), albeit with selectivity over other Nek family members. In addition, some activity was observed in cellular growth inhibition and centrosome separation assays [18]. More recently, an irreversible inhibitor (**5**) of Nek2 was identified *via* structure-guided design [19]. Interestingly, compound **5** was shown to react with the Cys22 residue of Nek2 and achieved sub-micromolar activity (Nek2 IC_50_ = 0.77 μM).

**Table 1:** Examples of reported small molecule Nek2 inhibitors

Number	Structure	Nek2 inhibitory activity IC_50_/μM	Reference
1	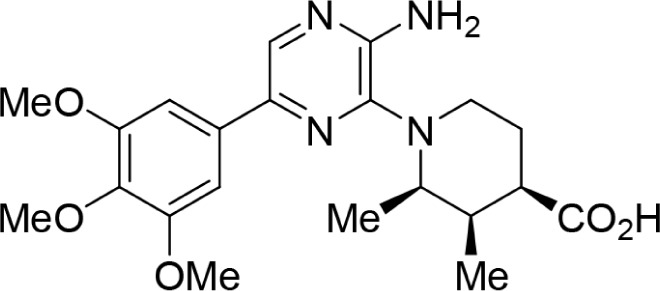	0.23	15
2	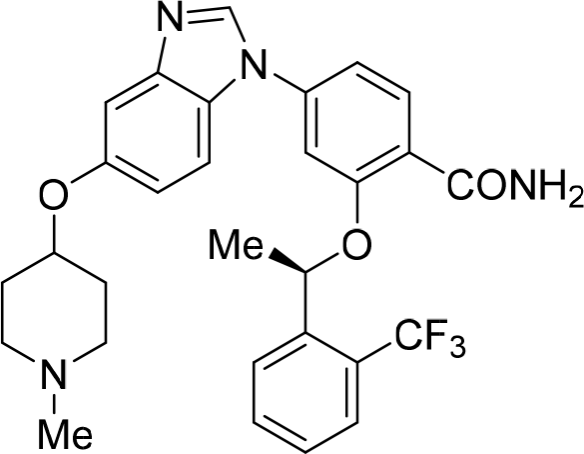	0.36	16
3	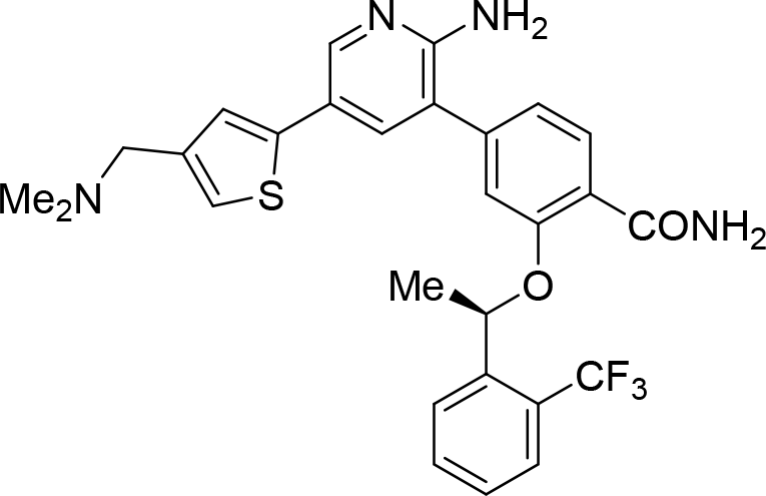	0.022	17
4	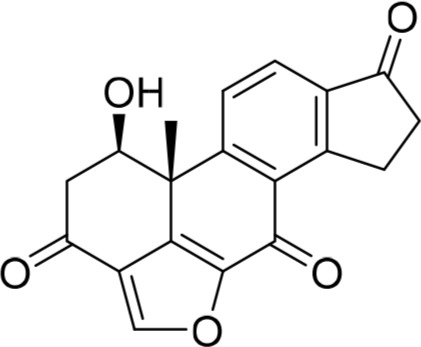	1.9	18
5	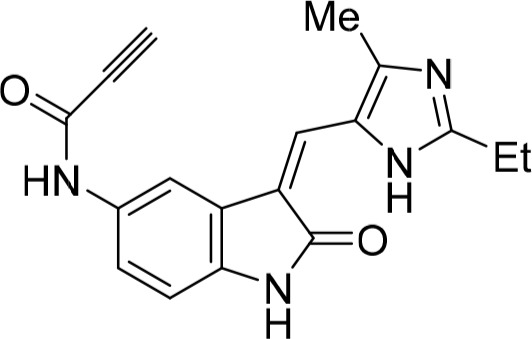	0.77	19
6	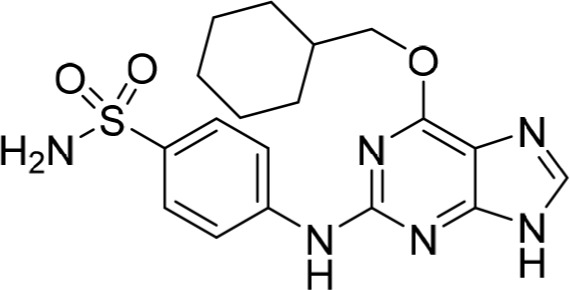	12.0	-
7	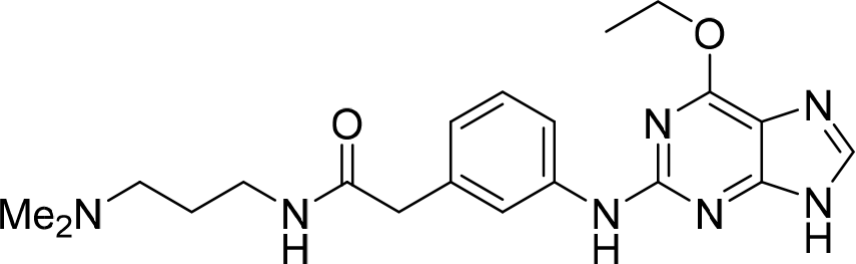	0.89	-

In the present study, a medium-throughput screen revealed that purines bearing 6-alkoxy substituents were ATP-competitive inhibitors of both Nek2 and CDK2 (*e.g*. **6**: CDK2 IC_50_ = 0.005 μM; Nek2 IC_50_ = 12 μM) (Table 1). Modifications around the purine scaffold were carried out to repurpose this class of kinase inhibitor to improve the potency against Nek2, whilst reducing activity against CDK2. Whilst it is acknowledged that a significant extent of structural homology exists between kinome members, and dissecting CDK2 inhibiton from Nek2 inhibition was expected to present a significant challenge, it was considered that structural differences between the two proteins could be highlighted using appropriately designed probes. From our initial screen, it was evident that in particular, purines bearing *meta*- or *para*-substituted 2-arylamino groups that contained a basic functionality afforded improved selectivity for Nek2 over CDK2 (*e.g*. **7**: CDK2 IC_50_ = 5.6 μM; Nek2 IC_50_ = 0.89 μM).

This paper describes extensive structure-guided design, synthesis and structure-activity relationship (SAR) studies conducted with the purine scaffold and directed towards the development of potent and selective reversible Nek2 inhibitors. These tools have provided an initial insight in to some of the key requirements for selective inhibition of Nek2 over CDK2 using purine-based inhibitors.

## RESULTS AND DISCUSSION

### Structure-guided inhibitor design

**Figure 1:**
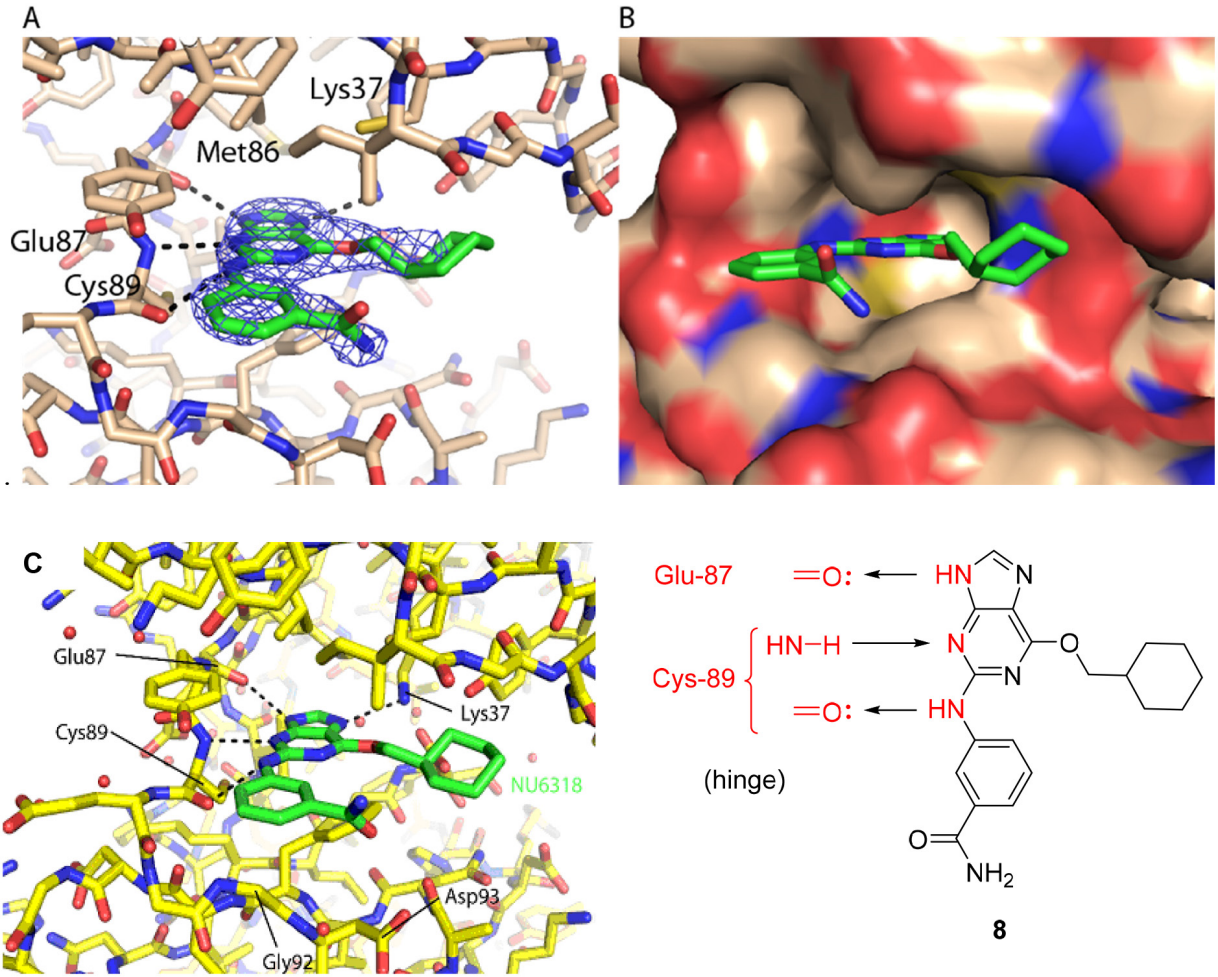
X-ray crystal structure of Nek2 in complex with 6-alkoxypurine inhibitor 8 **A**. View of compound **8** (carbon atoms coloured green) in the ATP-binding pocket of Nek2 (carbon atoms coloured beige). H-bonds are shown as dashed lines. A 2mF_o_-dF_c_ electron density map is shown as a blue wire-mesh around the compound. **B**. View of the ATP-binding pocket of Nek2 shown as a surface. **C**. Crystal structure of carboxamide **8** (green) bound to the T175A Nek2 mutant (carbon atoms are coloured yellow, oxygen coloured red, and nitrogen coloured blue). Hydrogen bonds are represented as dotted lines and important residues are highlighted.

As a starting point for understanding the mode of binding of arylaminopurines to Nek2, the X-ray crystal structure of **8**, identified from the initial screening, was determined in complex with Nek2 and revealed that the compound binds *via* a hydrogen bonding triplet between the purine N9-H, N3 and C2-NH, and the kinase hinge region residues Cys-89 and Glu-87 (Figure 1). Alkylation or removal of the participating purine nitrogen atoms would, therefore, be expected to be detrimental to activity towards Nek2 as for CDK2 and offer no basis for differentiation. However, the 6-alkoxy substituent was considered a candidate for remodeling of prototype inhibitors to differentiate between Nek2 and CDK2 inhibition. The 6-cyclohexylmethyl group occupies a lipophilic cavity near the ribose-binding pocket in CDK2 and is critical for activity [20]. A van der Waals contact may be formed between the 2-arylamino group and Gly-92. No definite interactions were observed between the amide functionality of **8** with the binding site, although the amide group is close to Asp-93 (Figure 1C), and it was considered that this may be exploitable.

**Figure 2:**
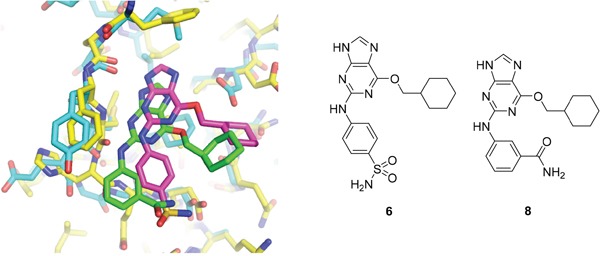
Structural overlay of 8-T175A-Nek2A complex (green and cyan) and the 6-T160pCDK2-cyclin A (pink and yellow) Rotation of the 2-arylamino group with respect to the purine is only observed for **6** in CDK2, but not for **8** in Nek2.

A comparison of the purines **6** and **8** in the CDK2 and Nek2 ATP-binding sites is shown in Figure 2. The aromatic ring systems of **8** in Nek2 are co-planar, whereas for **6** bound to CDK2 the 2-arylamino ring is rotated ~13° relative to the purine core due to sulfonamide interactions with Asp-86 (equivalent to Asp-93 of Nek2). Thus, interactions between the 3-benzamide moiety and the Nek2 binding site do not appear to affect the conformation of the purine. As a starting point for these studies, it was proposed that selective inhibition of Nek2 over CDK2 may be achieved through judicious modification of the purine 2-arylamino motif or the *O*^6^-substituent.

### Synthesis of purine-based probes for selective Nek2 inhibition

#### Modifications at the 2-Arylamino Position

Investigation at the purine 2-position began with the synthesis of simple 2-arylamino purine derivatives containing hydrogen-bond donors/acceptors, in which the substitution pattern around the aryl ring was varied and the linker length between the ring and various functionalities was altered. From the initial screening data, several structural motifs at this position showed Nek2 inhibition, including carboxamides, sulfonamides and ureas. These served as the targets for synthesis. Initial compounds were prepared by direct nucleophilic aromatic substitution of a purine 2-fluoro substituent of **9** with a range of anilines (see supporting information for synthesis of corresponding anilines where otherwise not shown); a second approach allowed diversification of some of these simple 2-arylamino-purines.

**Scheme 1:**
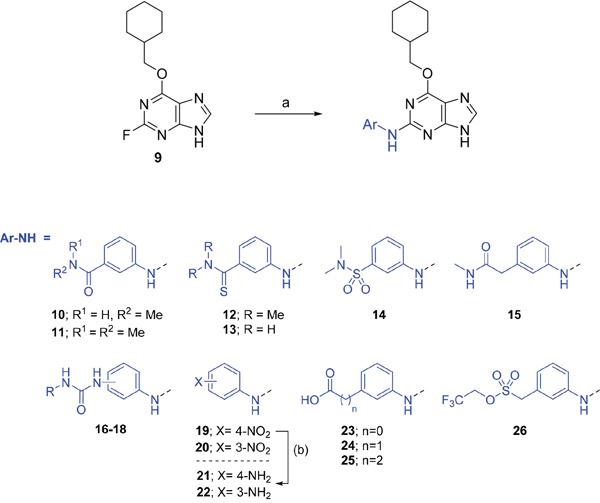
Synthesis of 2-substituted purine derivatives I.*^a^* *^a^* Reagents and conditions: (a) Appropriate aniline, TFA, 2,2,2-trifluoroethanol, 90°C, 18 h, 17-77%; (b) Pd/C, H_2_, MeOH, RT, 18 h.

**Scheme 2:**
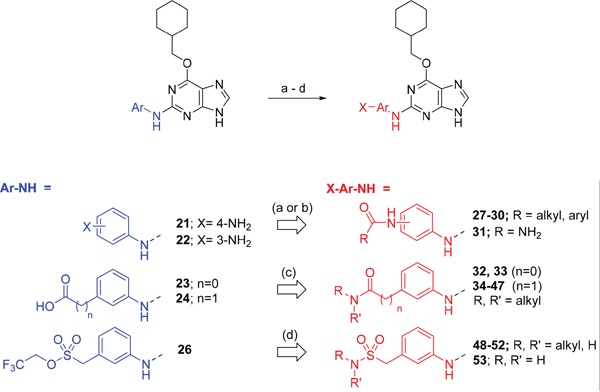
Synthesis of 2-substituted purine derivatives II.*^a^* ^a^ Reagents and conditions: (a) i. RCOCl, Et_3_N, DMAP, THF, RT (or 70°C as required), 18 h, 31-81%; ii. TFA, DCM, RT, 18 h, 43 - 100%; (b) i. NaOCN, TFA, DMF, RT, 18 h; ii. TFA, DCM, RT, 18 h; (c) i. CDI, DIPEA, DMF, RT, 90 min; ii. RR'NH, RT, 18 h, 20-81%; (d) i. DBU, RR'NH, THF, MW, 160°C, 15 min; ii. (for compound **53**) **52**, TFA (neat), RT, 6 h, 85%.

As one of the original hits from the screen was a carboxamide derivative, it was decided to begin this study by synthesising analogues of the carboxamide to investigate how this may be modified. Carboxamides **10** and **11** and related thiocarboxamides **12** and **13** were prepared by reaction of fluoropurine **9**, with either a commercially available or a synthesized (see ESI) aniline, in 2,2,2-trifluoroethanol (TFE) containing trifluoroacetic acid (TFA) [20–22] (Scheme 1). A carboxamide isostere, the thiocarboxamide group, was investigated as the sulfur atom of a thioamide is a weaker hydrogen bond acceptor than the oxygen atom of an amide, but has a stronger hydrogen bond donating ability at the NH [23]. The sulfonamide equivalent of carboxamide **11** was synthesized *via* a similar route affording **14**. To probe the effect of sidechain homologation of compound **10**, *N*-methyl-homocarboxamide **15** was prepared from the corresponding precursor, 3-aminophenyl-(*N*-methyl)-acetamide (see ESI). Compounds **16-18**, comprising a urea motif bearing linkers to a terminal basic group, were also prepared according to the standard method using synthesised anilines (see ESI). *Para*- and *meta*-nitrophenylaminopurines **19** and **20** were prepared in the same manner and subsequently converted in to the amino compounds (**21** and **22**) by catalytic hydrogenation. Anilines **21** and **22**, carboxylic acids **23** and **24** and sulfonate ester **26** were prepared principally for further elaboration of the 2-arylamino sidechain (Scheme 2).

As indicated*, para*- and *meta*-aminophenylaminopurines **21** and **22** (Scheme 1) were treated with acyl chlorides to provide the desired *N*-acyl aniline ‘reversed amide’ derivatives **27**-**30** without purine *N*-9 protection (Scheme 2). In addition to the desired products, the reaction also afforded an unstable acylation product at the purine *N*-9, which was easily removed by treatment with TFA. This allowed the investigation of the exact preferences for the amide group orientation, as well as the substitution position at the 2-arylamino group. To investigate whether a second hydrogen bond-donor group could form additional favourable interactions over that of a simple carboxamide and to quantify this in comparison with more complex urea derivatives, the parent urea *e.g*. **31** was prepared. Arylureas were synthesised by treatment of precursor aniline **22** with isocyanic acid (HN=C=O), generated *in situ* from sodium cyanate and TFA (Scheme 2) [24]. As previously observed within the reversed amides series, an undesired urea product was also formed at the purine N-9 and was cleaved by treatment with TFA.

For the synthesis of a focussed set of homocarboxamides a convergent multiple-parallel approach was undertaken (Scheme 2). Using carboxylic acids **25** and **26** a library of amides (**32-47**) was obtained by coupling with aliphatic or aromatic amines [25, 26]. To further understand the effect of homologation of the hydrogen bond donor-acceptor group, a series of *N*-2-arylmethanesulfonamides (**48-52**) was also synthesized from the parent 2,2,2-trifluoroethanesulfonate **26** (Scheme 2). To complete the SARs, the *meta*-substituted methylsulfonamide **53** was also synthesized by deprotection of the *p*-methoxybenzyl (PMB) group of **52**.

### Modifications at the *O*^6^-Alkyl Position

#### Deletion and contraction of the 6-cyclohexylmethyl substituent

**Figure 3:**
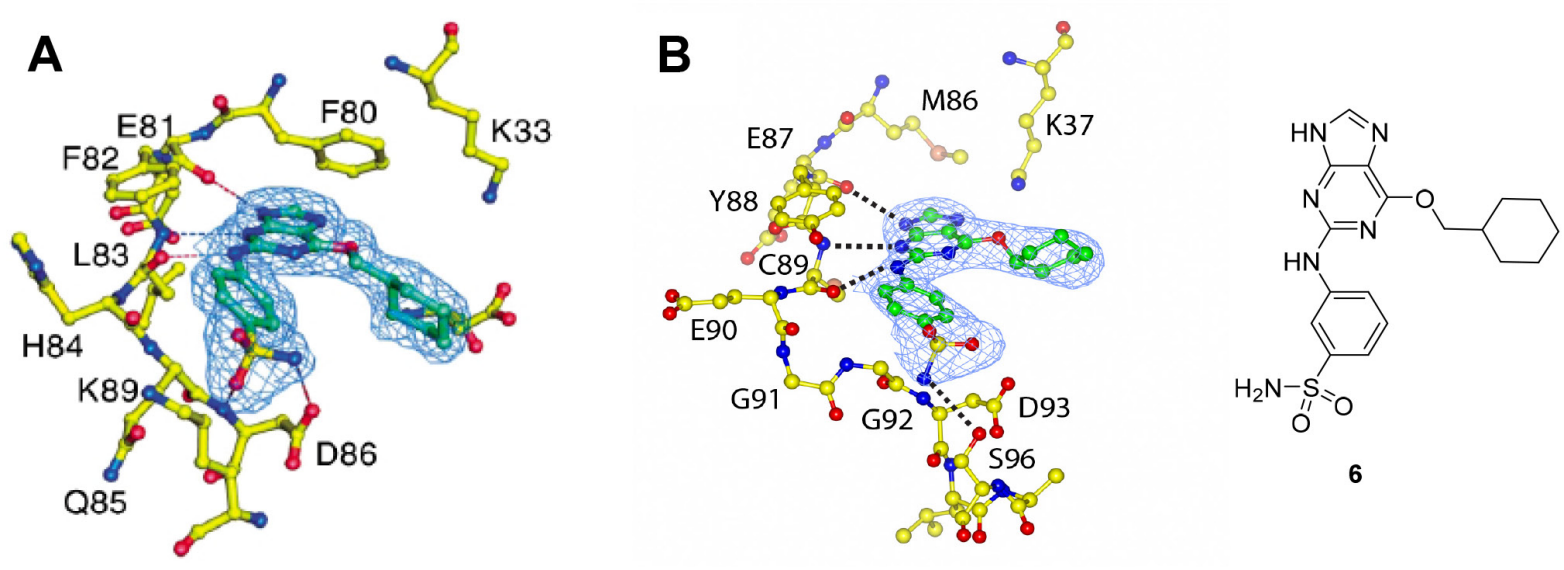
Binding of purine 6 to: **A**. active Thr 160-phosphorylated CDK2-cyclin A complex (T160pCDK2-cyclin A); and **B**. inactive Nek2.

Crystal structures of Nek2 and CDK2 suggested that the *O*^6^-cyclohexylmethyl group was more important for CDK2 inhibition than Nek2, and indicates that varying the size and conformation of the purine 6-substituent might enable discrimination between Nek2 and CDK2. Furthermore, a suitable group at this position could possibly make a favorable interaction with Lys-37 (K37), which is near the ribose binding pocket of Nek2 (Figure 3B). Although CDK2 also has a lysine residue (K33) near the ribose site (Figure 3A), its disposition and environment differs and so substituents at the purine 6-position could increase Nek2 selectivity by exhibiting a selective impact on its Lys-37.

**Scheme 3:**
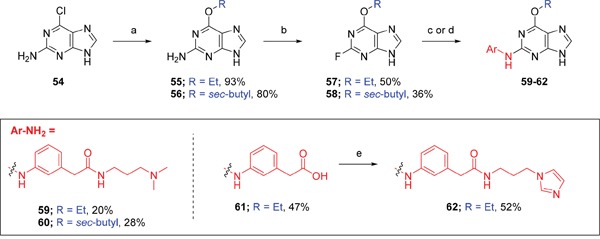
Synthesis of *O*6-alkyl-2-arylaminopurines.*^a^^a^* Reagents and conditions: (a) ROH, Na, reflux, 18 h; (b) HBF_4_, NaNO_2_, H_2_O, 0°C → RT, 24 h; (c) (i) 3-aminophenylacetic acid, TFA, 2,2,2-trifluoroethanol, 90°C, 24 h, (ii) NaOH, THF/H_2_O,RT, 18 h; (d) TFA, 2,2,2-trifluoroethanol, 90°C, 18 h; (e) (i) CDI, DIPEA, DMF, RT, 90 min, (ii) 1-(3-aminopropyl)imidazole, RT, 18 h

Purines bearing *O*^6^-alkyl substituents, ethyl and *sec*-butyl (**59** and **60**, respectively), were prepared from the 6-chloropurine precursor (**54**), by 6-position substitution with alkoxides, followed by addition of anilines at the 2-position that had been found to confer Nek2 inhibition (Scheme 3). To overcome some purification problems encountered during the synthesis of purines with dimethylaminopropyl side chains, carboxylic acid **61** was synthesized and used without further purification in the preparation of 1-(3-aminopropyl)imidazole derivative **62**.

**Scheme 4:**
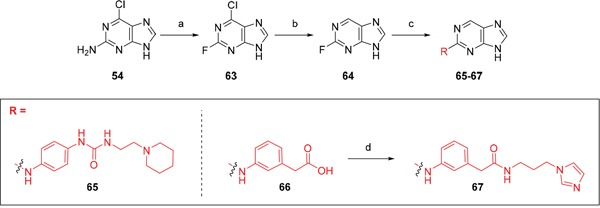
Synthesis of 6-unsubstituted 2-arylaminopurines.*^a^* ^a^ Reagents and conditions: (a) HBF_4_, NaNO_2_, 0°C to RT, 75 min, 75%; (b) Pd(OH)_2_, NH_4_OOCH, MeOH, 65°C, 1 h, 100%; (c) (i) anilines, TFA, 2,2,2-trifluoroethanol, 90°C, 48 h; (ii) for **66**: KOH, THF/H_2_O, RT, 18 h 34-45%; (d) (i) CDI, DIPEA, DMF, RT, 90 min; (ii) RNH_2_, RT, 18 h, 40 – 59%

To provide a reference point for these studies, the 6-substituent was deleted entirely. Thus, the 6-unsubstituted intermediate **64** was prepared from 2-fluoro-6-chloropurine (**63**) [27], by selective dehalogenation of the 6-chloro group using catalytic transfer hydrogenation [28, 29]. Coupling of **64** with the appropriate anilines gave derivatives **65** and **66**, with **66** being converted to amide **67** (Scheme 4).

#### Replacement of the *O*^6^-cyclohexylmethyl substituent

**Scheme 5:**
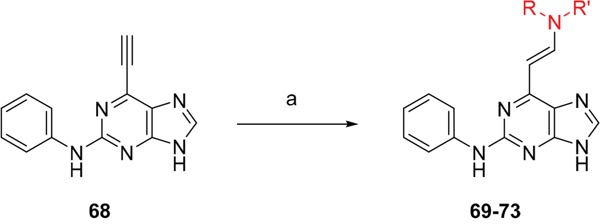
Synthesis of 6-(dialkylamino)vinyl-purines.*^a^* ^a^ Reagents and conditions: (a) RR′NH, THF, microwave 100°C, 10 min, 60 – 98% (Supplementary Table 1)

Given the differences between the ribose-binding domains of Nek2 and CDK2, it was considered that potency may be improved and off-target activity reduced, if the group at C-6 could be conformationally restricted by the introduction of an alkene. This premise was explored by synthesis of (*E*)-6-(2-dialkylaminovinyl)purines (**69-73**). 6-Ethynyl- and 6-vinylpurines have been reported as substrates for conjugate addition reactions with secondary amines affording (*E*)-enamines or ethylamines, respectively [30, 31]. Addition of primary amines to a 6-ethynyl group afforded an inseparable mixture of (*Z*)- and (*E*)-enamines as a consequence of imine-enamine tautomerism. Reported conditions for this transformation utilized purine *N*-9 protection and typically required lengthy reaction times of 1-3 days at room temperature [30]. We found that the addition of amines to the 6-ethynylpurine **68** could be accelerated by microwave heating (Scheme 5). 6-Ethynylpurine **68** was prepared from *N*-9 THP-protected 2-fluoro-6-chloropurine utilizing Sonagashira alkynylation. Following cleavage of the *N*-9 hemiaminal ether by acidic hydrolysis, introduction of the 2-arylamino group and silyl group deprotection afforded the alkynyl intermediate. In all cases the subsequent conjugate addition took place smoothly in moderate-to-high yield without *N*-9 protection and a series of (*E*)-enamines (**69-73**) was obtained (see ESI for more examples). The configuration at the newly formed double bond was validated by ^1^H NMR (^3^*J* = 15.0 Hz). This methodology is applicable for the facile synthesis of enamine derivatives from a diverse set of secondary amines.

#### Biological evaluation of synthesized purines

Preliminary data attained following a medium-throughput screening campaign, identified purines bearing 6-alkoxy substituents (*e.g*. **6**; CDK2 IC_50_ = 0.005 μM; Nek2 IC_50_ = 12 μM) as dual inhibitors of Nek2 and CDK2. Interestingly, deletion of the sulfonamide moiety from **6**, resulted in a 200-fold reduction in CDK2 inhibitor activity as well as some reduction in Nek2 activity (2-aminophenyl-6-(cyclohexylmethoxy)-9*H*-purine: CDK2 IC_50_ = 1 μM; Nek2 IC_50_ = 22 μM). To gain a further understanding of the requirements for selective Nek2 inhibition, a structure-activity study was undertaken to explore the scope for modification of the purine hits at the 2- and 6-positions.

#### Modifications at the 2-arylamino position

We have previously reported that hydrogen bond donor-acceptor groups, such as sulfonamides at the 2-arylamino position, form important interactions (*e.g*. with Asp-86) at the ‘specificity surface’ of CDK2 [32]. To discriminate between the interactions required for Nek2 inhibition and CDK2 inhibition, variations of the 2-arylamino substitution were studied by synthesis of acetamide, carboxamide, sulfonamide and urea derivatives. Both 4- and 3-nitro-substituted precursors were inactive against Nek2 yet retained modest potency against CDK2 (**19**; CDK2 IC_50_ = 1.9 μM; Nek2 IC_50_ = > 50 μM and **20**; CDK2 IC_50_ = 0.89 μM; Nek2 IC_50_ = > 50 μM, respectively). The corresponding aniline improved activity against Nek2 in both cases (**21**; CDK2 IC_50_ = 0.22 μM; Nek2 IC_50_ = 6.3 μM and **22**; CDK2 IC_50_ = 0.7 μM; Nek2 IC_50_ = 5.9 μM, respectively), suggesting that there may be a hydrogen-bonding interaction available in the Nek2 ATP-binding domain. Activity was not enhanced by acylation of the respective aniline compounds, with *N*-acetyl compound **27**, showing only weak activity against Nek2. Further homologation and branching of the acyl group of the 4-substituted derivatives did not enhance Nek2 inhibition (*e.g*. **28**), and substantial loss of Nek2 inhibitory activity occurred with benzoyl (**29**) or isonicotinoyl (**30**) groups.

The 3-substituted purine **8** had modest activity against CDK2 (IC_50_ = 0.48 μM) and weak activity against Nek2 (IC_50_ = 19 μM). Addition of a methyl substituent at the amide nitrogen (compound **10**) reduced potency against CDK2 but only marginally increased Nek2. Reversing the orientation of the carboxamide group (**27**; CDK2 IC_50_ = 0.88 μM; Nek2 IC_50_ = 8.3 μM) increased Nek2 inhibition in comparison to **8**, without increasing activity against CDK2. However, unexpectedly, addition of a second methyl group to produce the dimethylamide **11** afforded a marked increase in Nek2 inhibitory activity whilst also greatly reducing CDK2 inhibition, suggesting that the amide NH hydrogen bond may not be required for Nek2 inhibition. Although tethering the dimethylamino group into a ring, as in piperidine derivative **32**, further reduced CDK2 inhibitory activity, Nek2 inhibition was also abolished.

Replacing the carboxamide group by a carboxylate (**23**) increased Nek2 activity (IC_50_ = 4.3 μM) compared to **8**, indicating a possible ionic interaction with the carboxylate and that one hydrogen bond donor is optimal. However, **23** still retained potency against CDK2 (IC_50_ = 0.99 μM). The thioamides **12** and **13** were similar in potency to their amide equivalents (**11** and **8**) against CDK2, but were less active against Nek2.

**Figure 4:**
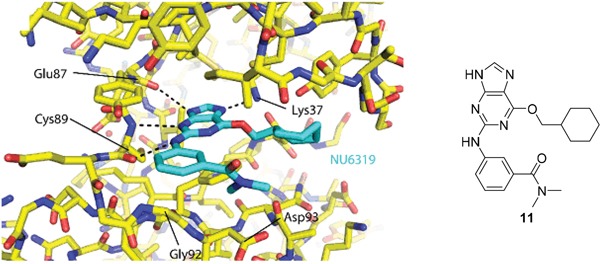
Crystal structure of carboxamide 11 (cyan) bound to the T175A Nek2 mutant (carbon atoms are coloured yellow, oxygen coloured red, and nitrogen coloured blue) Hydrogen bonds are represented as dotted lines and important residues are highlighted.

Overall, the SARs around the amides, benzamides and thioamides at the *meta* position of the 2-arylamino-*O*^6^-cyclohexylmethylpurines revealed that a hydrogen bond donor at the *meta* position favoured activity against CDK2 (*e.g*. **8**, **10, 13, 23**), whereas introducing a larger, bulkier substituent (**32**) was unfavourable for activity against both CDK2 and Nek2 (Table 2). Furthermore, the dimethylcarboxamide **11** was both the most potent Nek2 inhibitor and the compound with the greatest Nek2/CDK2 selectivity (> 10-fold) in this series. These observations could possibly be rationalised through a putative interaction with Asp-93 (Figure 4), and may also be the case with primary carboxamide **8** in Figure 1; however, no direct evidence for this supposition has yet been attained.

With the homocarboxamide series (Table 2, sub-structure C), removing the donor NH of the amide group by synthesis of di-substituted carboxamides (**37** and **38**) in general retained affinity for CDK2 but not Nek2. This observation was clearly shown by the matched pair of compounds **36** and **37**, where a methyl group replaced a hydrogen bond donor and markedly reduced activity against Nek2 but not CDK2. Increasing the size of the di-substituted amides decreased Nek2 activity further whilst retaining modest CDK2 activity (*e.g*. **38**), indicating that hydrophobic interactions may be particularly unfavourable for Nek2 inhibitory activity in this region. A similar decrease in potency was observed for Nek2 and CDK2 (~2-fold) when the size of the alkyl ring of cyclic secondary amides was increased (**35** and **36**). Compound **15** possesses the amide NH hydrogen bond donor but had a higher potency against CDK2. Replacing the methyl group of **15** with *n*-propyl (**39**) reduced activity against Nek2 whilst maintaining CDK2 inhibition. The *iso*-butyl analogue (**34**) was also less active against Nek2 than **15** but equipotent to **39**, indicating that the Nek2 active site is sterically limited. In contrast, the effect on CDK2 inhibition was limited (IC_50_ values ranging from 0.3-0.9 μM).

**Table 2:** Inhibition of CDK2 and Nek2 by representative 2-arylamino-*O*^6^-cyclohexylmethylpurines.*^a^*

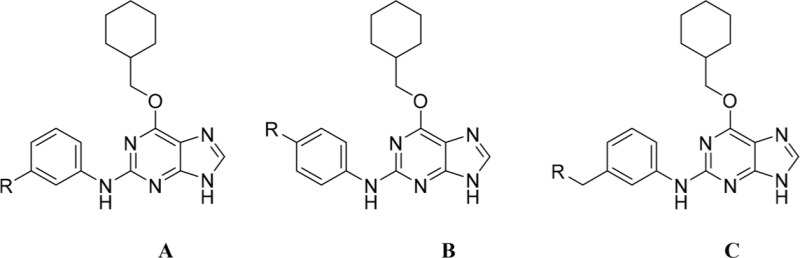									
Compound	Sub-structure	R	IC_50_ (μM) / % inhibition		Compound	Sub-structure	R	IC_50_ (μM) / % inhibition	
			Nek2	CDK2				Nek2	CDK2
**8**	A	CONH_2_	19	0.48	**35**	C	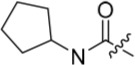	4.1	1.1
**10**	A	CONHCH_3_	15	0.83	**36**	C	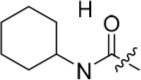	8.4	2.1
**11**	A	CON(CH_3_)_2_	0.62	7.0	**37**	C	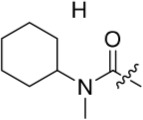	26% @ 50 μM	1.6
**12**	A	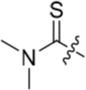	5.2	5.7	**38**	C	CON(*i*-Pr)_2_	21	3.2
**13**	A	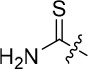	46% @ 50 μM	0.33	**39**	C	CONH-n-Pr	82% @ 50 μM	0.74
**14**	A	SO_2_N(CH3)_2_	40% @ 50 μM	3.5	**40**	C	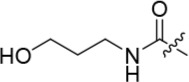	1.3	0.8
**15**	C	CONHCH_3_	1.3	0.32	**41**	C	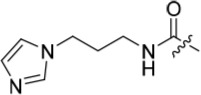	6.13	1.6
**16**	B	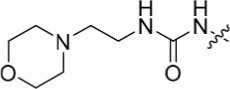	2.2	1.1	**42**	C	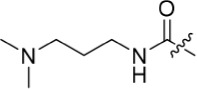	0.28	1.0
**17**	B	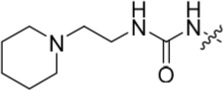	1.1	0.86	**43**	C	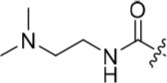	1.0	0.59
**18**	B	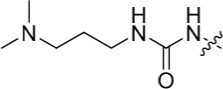	1.2	0.72	**44**	C	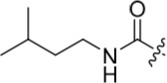	29% @ 50 μM	3.7
**23**	A	CO_2_H	4.3	0.99	**45**	C	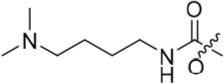	0.7	0.86
**27**	A	NHCOMe	8.3	0.88	**46**	C	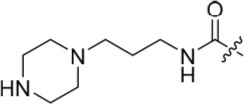	3.6	2.4
**28**	B	NHCO^*t*^Bu	24	9.7	**47**	C	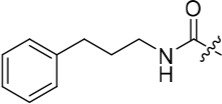	Inactive	10
**29**	B	NHCOPh	36% at 50 μM	2.9	**48**	C	SO_2_N(Me)_2_	0.83	0.77
**30**	B	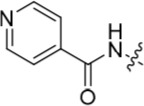	51% at 50 μM	2.2	**49**	C	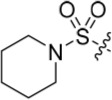	53% @ 50 μM	1.6
**31**	A	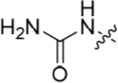	3.6	0.38	**50**	C	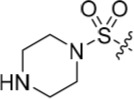	2.6	0.45
**32**	A	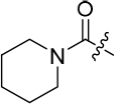	57% @ 50 μM	18	**51**	C	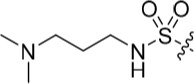	1.42	0.53
**33**	A	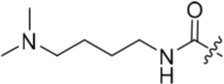	3.8	0.73	**52**	C	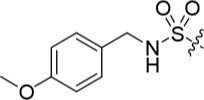	38% @ 100 μM	3.0
**34**	C	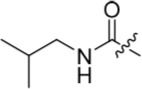	79% @ 50 μM	0.94	**53**	C	SO_2_NH	81% @ 50 μM	0.24

All IC_50_ values are results obtained from n = 3 determinations.

^*a*^See also Supplementary Table S1 (ESI) for complete compound data.

^*b*^Nek2 Caliper assay conducted at 30 μM ATP concentration – full details in reference 12.

^*c*^CDK2 assay conducted at 12.5 μM – full details in reference 16.

Introducing a terminal hydroxy group on the propyl chain (**40**) was tolerated for Nek2 inhibition, and a dimethylamino group (**42**) at the end of the propyl side chain increased activity against Nek2. Shortening the alkyl chain of **42** from propyl to ethyl (**43**) did not improve Nek2 inhibition, and removal of the amino functionality of **43** to give **44** greatly reduced Nek2 inhibition, suggesting that the basicity of the tertiary amino group was required. In summary, these results indicated the possibility of an additional interaction in the Nek2 binding site for a basic moiety at the end of the propyl chain.

Selectivity for CDK2 over Nek2 was observed for the sulfonamides shown in Table 2 with the exception of **48**, which was equipotent against both CDK2 and Nek2. The primary sulfonamide **53** exhibited good potency against CDK2 and some activity against Nek2. Cyclic secondary sulfonamides (*e.g*. **49**) were essentially inactive against Nek2 except for heterocyclic derivatives (*e.g*. **50**), which showed modest activity against Nek2. Nevertheless, all of the sulfonamides tested were more potent against CDK2 than Nek2. Inhibition of Nek2 by **50** could be attributed to the basic properties of the piperidine ring, which appears to be favourable for Nek2 activity. Compound **49** supports this observation given its lower activity against Nek2. Larger lipophilic substituents (*e.g*. **52**) resulted in greatly decreased activity against Nek2 and variable effects on CDK2 activity. In comparison to the most potent carboxamide (**42**) the corresponding sulfonamide (**51**) was both less active (5-fold) and less selective for Nek2.

The urea moiety was also investigated as a putative non-classical isosteric replacement for the sulfonamide functional group. 6-Alkoxy-2-arylaminopurines with urea-based side-chains exhibited low-micromolar activity against Nek2, as compared to micromolar or sub-micromolar inhibition of CDK2; as exemplified by **16** (Nek2 IC_50_ = 2.2 μM, CDK2 IC_50_ = 1.1 μM). Disappointingly, primary ureas *e.g*. **31** were found to be 10-fold selective for CDK2 over Nek2. Elaboration of this group bysynthesis of secondary ureas *e.g. N*-ethylmorpholine derivative **16**, enhanced activity against Nek2. Replacement of the *N*-ethyl-morpholino group by *e.g*. *N*-ethyl-piperidine (**17**) and dimethylaminopropyl (**18**) side-chains, did not greatly affect inhibition or selectivity profile for Nek2 or CDK2. These results suggest that whilst, potency against Nek2 may be gained from incorporation of a basic group and a donor-acceptor moiety at the 2-arylamino position, the additional hydrogen-bond acceptor in the morpholine is unnecessary or even detrimental.

#### Modifications at the purine 6-position

In order to improve potency against Nek2 whilst retaining the selectivity observed within some examples from the homocarboxamide series *e.g*. **42**, alternative substitution at the 6-position was investigated (Table 3). In comparison to **42**, selectivity for Nek2 over CDK2 was maintained by combining an *O*^6^-ethyl substituent with the dimethylaminopropyl homocarboxamide side chain (**59**;CDK2 IC_50_ = 5.6 μM; Nek2 IC_50_ = 0.89 μM), albeit with some loss of potency against both kinases. Imidazole derivative **62** exhibited 2-fold improved Nek2 inhibitory activity combined with a 2-fold reduction in CDK2 inhibition, compared with the analogous cyclohexylmethyl derivative **41**. Interestingly, the *O*^6^-*sec*-butyl derivative with a *N,N*'-dimethylaminopropyl side chain **60** gained potency against CDK2 with 10-fold selectivity over Nek2.

**Table 3:** Nek2 and CDK2 inhibition by selected 6-unsubstituted and 6-substituted purines. ^*a*^

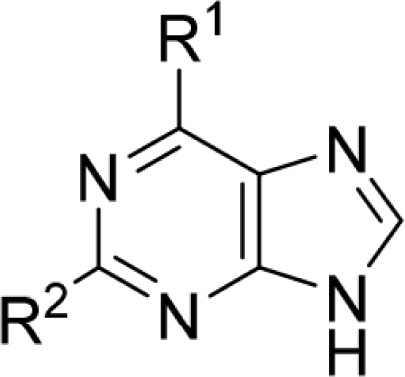				
Compound	R^1^	R^2^	IC_50_ (μM) or % inhibition	
			Nek2(30 μM ATP) ^*a*^	CDK2(12.5 μM ATP) ^*b*^
**59**	OEt	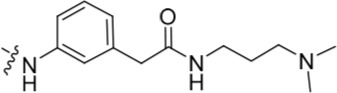	0.89	5.6
**60**	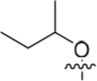	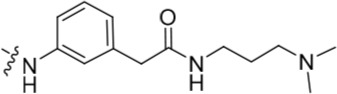	4.9	0.41
**62**	OEt	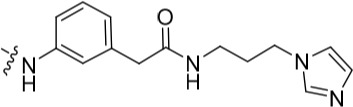	2.8	5.2
**65**	H	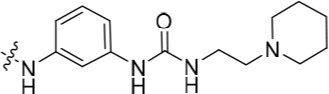	4.5	30% @ 100 μM
**67**	H	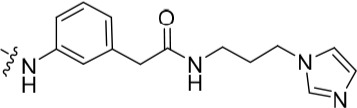	5.9	>100
**69**	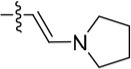	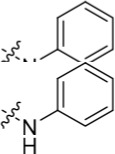	0.34	5.8
**70**	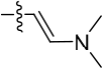		0.77	3.0
**71**	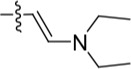	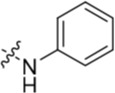	0.24	0.65
**72**	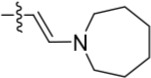	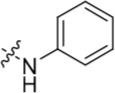	0.27	2.7
**73**	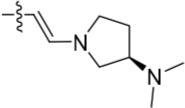	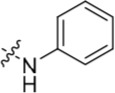	3.0	6.9

All IC_50_ values are results obtained from n = 3 determinations.

^*a*^See also Supplementary Table S2 (ESI) for complete compound data.

^*b*^Nek2 Caliper assay conducted at 30 μM ATP concentration – full details in reference 12.

^*c*^CDK2 assay conducted at 12.5 μM – full details in in reference 16.

Guanine derivatives lacking the *O*-alkyl substituent (**65** and **67**) exhibited a dramatic reduction in potency against CDK2 compared with the parent 6-alkoxy compounds, yet Nek2 inhibition was maintained. The 6-unsubstituted purine derivative bearing a substituted urea side-chain (**65**)was approximately 4-fold less potent against Nek2 than the parent 6-alkoxypurine **17** but, importantly, gave only 30% inhibition of CDK2 at 100 μM. Activity against Nek2 was also maintained for the 6-unsubstituted imidazole homocarboxamide purine (**67**), and CDK2 inhibitory activity was again abolished. This result indicated that a 6-cyclohexylmethyl substituent conferred potent CDK2 inhibition but was not necessary for Nek2 inhibition. A significant improvement in selectivity was gained as a result of this modification, confirming that there are exploitable differences between the ribose-binding pockets of Nek2 and CDK2.

**Figure 5:**
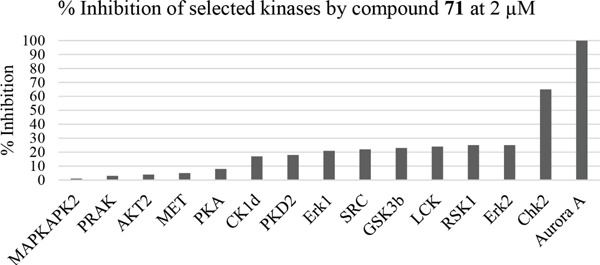
Counter-screening of 71 against a panel of kinases

**Figure 6:**
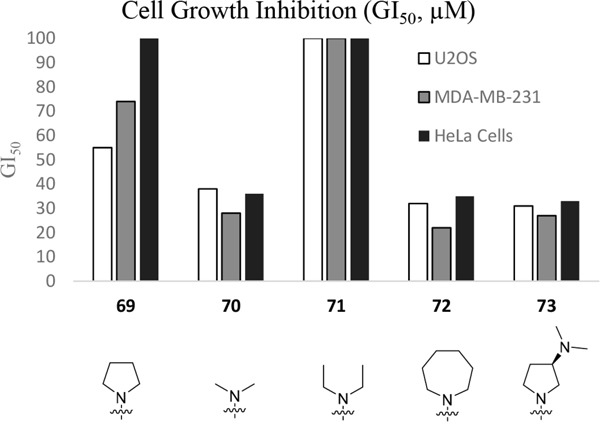
Growth inhibition (GI50) of selected cell-lines by enamines 69-73

Having generated a series of enamines to probe the replacement of the *O^6^*-alkoxy substituent, SARs revealed that polar substituents, *e.g*. morpholine (see ESI), and hydrophobic alkyl substituents such as those in compounds **69**-**71** were tolerated. Larger cyclic secondary amines such as homopiperidine (**72**) were found to be sub-micromolar inhibitors of Nek2 (IC_50_ = 0.27 μM). However, the addition of a second basic group as in 3-dimethylaminopyrrolidine (**73**) reduced the inhibition of Nek2 around 10-fold as compared with **69**. The CDK2 counter-screening data were largely unremarkable as the compounds **69**-**73** were only low-micromolar CDK2 inhibitors with the exception of the diethylamino compound **71** (IC_50_ = 0.65 μM). From the data obtained, it appeared that the most potent enamine-based Nek2 inhibitors were generally also more potent against CDK2. To assess the broader kinase selectivity of this series of 6-(dialkylamino)vinyl-purines, the diethylamino derivative **71** was chosen for wider screening against 24 kinases at 2 μM inhibitor concentration (Figure 5 – method as in reference 12). Compound **71** did not significantly inhibit other kinases studied with the exception of the mitotic kinases aurora A (100% inhibition at 2 μM) and Chk2 (65% at 2 μM). If **71** is representative of the entire series, this may indicate a lack of selectivity for Nek2. In a separate counter-screen, dimethylamino derivative **70** was found to inhibit weakly another mitotic kinase, Plk1 (41% at 10 μM – method as in reference 12). Cell growth inhibition studies (Figure 6) with compounds **69-73** in human tumor cell lines, including U2OS, MDA-MB-231 and HeLa cells, showed modest effects on cell viability (GI_50_ > 10 μM). Interestingly, the cellular activity did not reflect the relative potencies in the Nek2 kinase assay and may indicate that cell growth inhibition is due to inhibition of a combination of Nek2 and other kinases *e.g*. aurora A.

**Figure 7:**
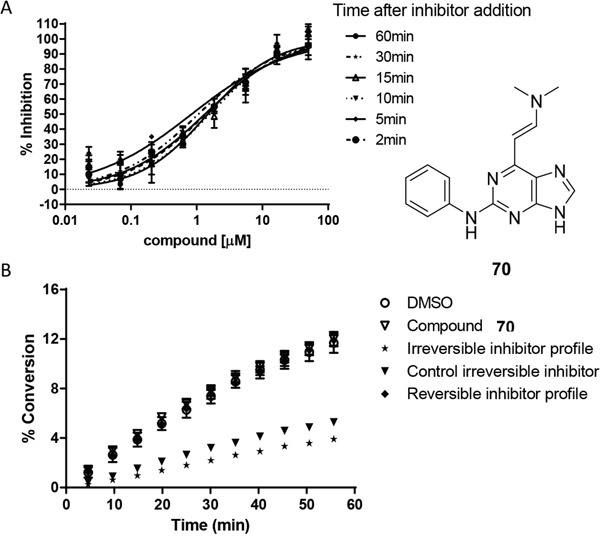
Mechanism of action studies with enamine 70 **A**. The effect of pre-incubation (2-60 min) of Nek2 with **70** on inhibitory potency. **B**. Reversibility studies indicating that **70** was not an irreversible inhibitor of Nek2 (Supplementary Table 4).

A representative enamine (**70**) was shown not to exhibit time-dependent Nek2 inhibition kinetics (Figure 7A (see also ESI), indicating that the compound binds reversibly to Nek2 and that the enamine is not chemically reactive within the Nek2 active site. This observation was also confirmed by kinetic studies of kinase inhibition, which showed that removal of the inhibitor by rapid dilution of the assay medium, restored kinase activity (Figure 7B) [33].

**Figure 8:**
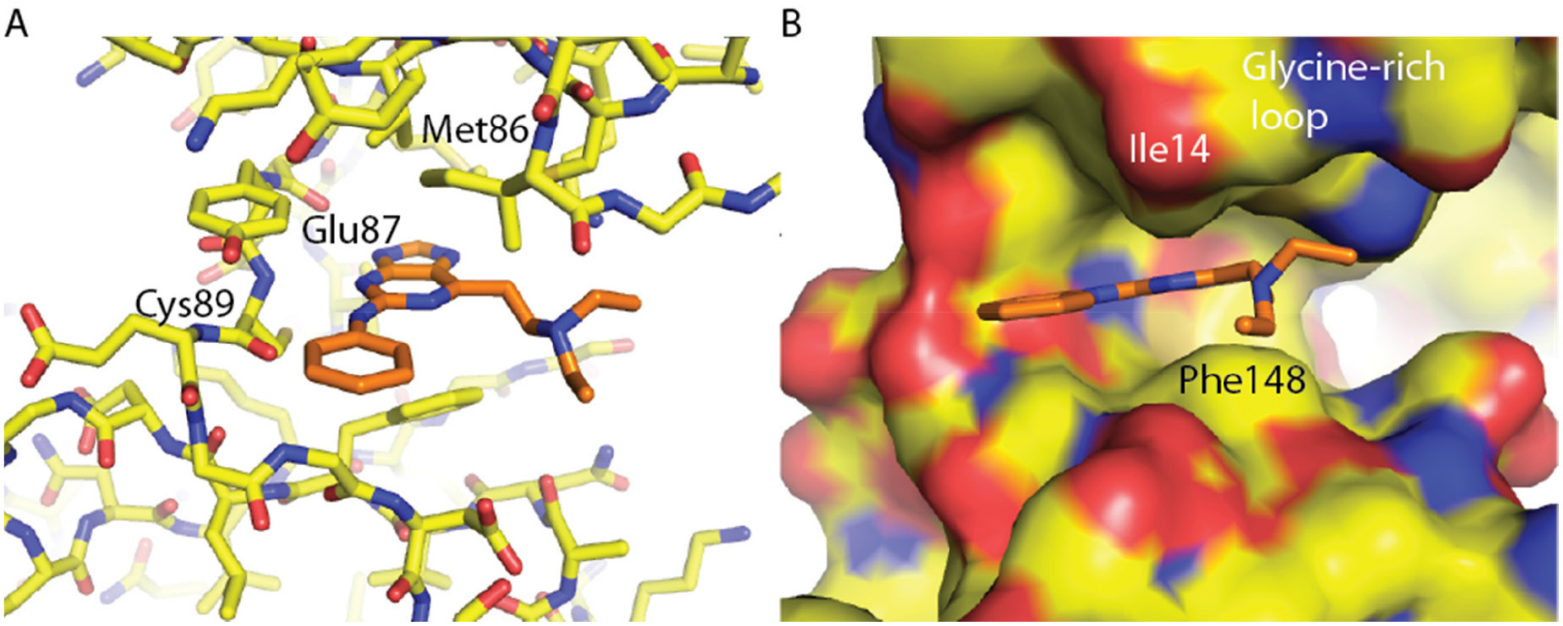
Crystal structures of compound 71 in complex with Nek2 **A**. Interaction of 6-(2-aminovinyl)purine derivative **71** (carbon atoms coloured orange) with the hinge region of Nek2. **B**. Orientation of the C-6 substituents towards the glycine-rich loop.

Structural biology studies confirmed that the 6-(2-aminovinyl)purine Nek2 inhibitors occupied a binding orientation nearly identical to that of the *O*^6^-alkylpurines. The interactions between the purine and the kinase hinge motif were clearly visible (Figure 8A) and the enamine 6-substituent was shown to point towards the hydrophobic glycine-rich loop (Figure 8B), which is consistent with the *O^6^*-alkyl purine hits from the initial screening results.

#### Stability of enamines

**Figure 9:**
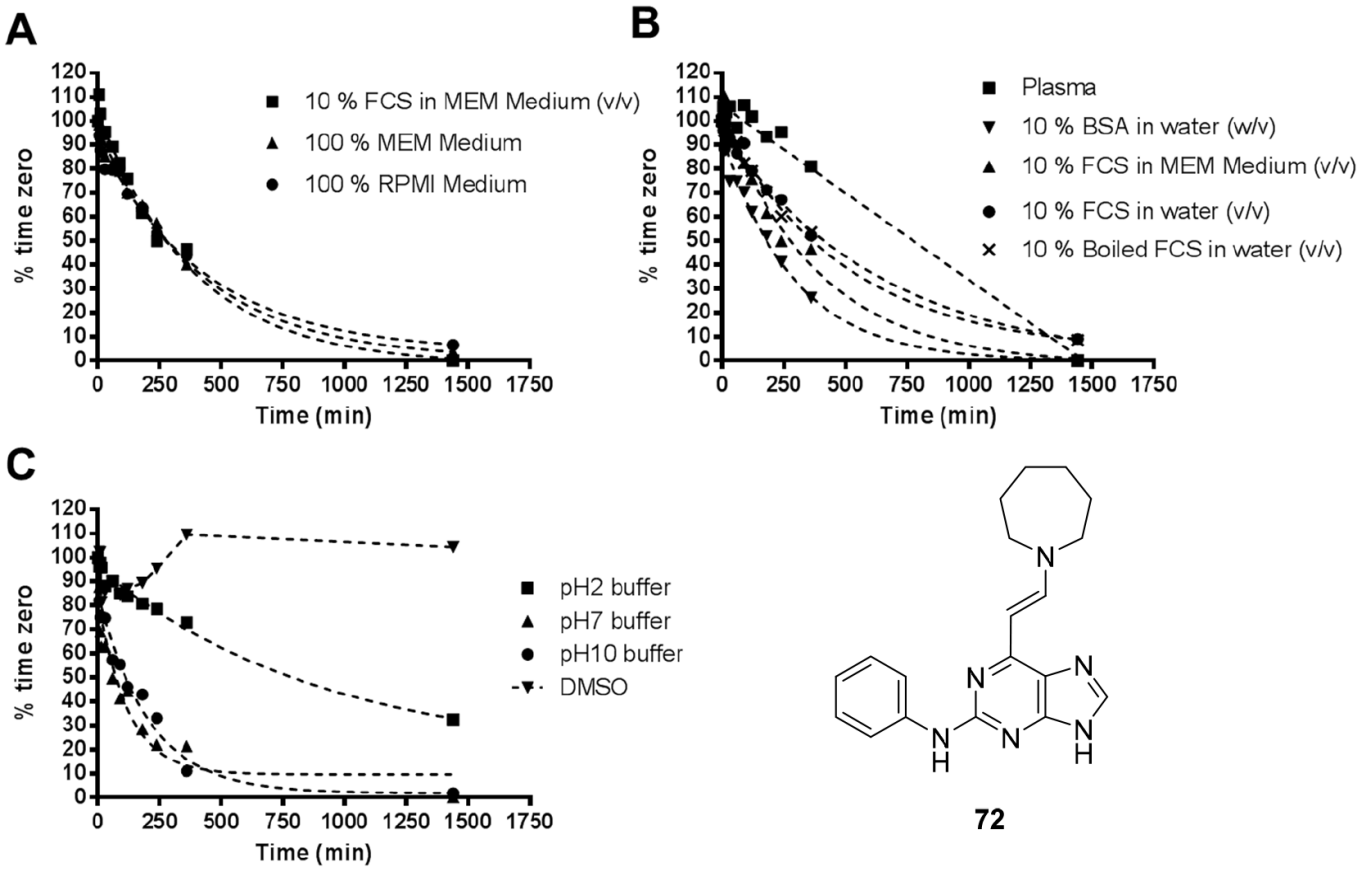
Stability of 72 in various assay media BSA (bovine serum albumin) and FCS. Note: MEM (minimum essential medium) and RPMI (Roswell Park Memorial Institute) media contain no proteins or enzymes.

To determine whether the enamines were hydrolysed under the assay conditions, the stability of representative enamine derivative **72** was assessed in a range of assay media and pH buffers. Stock solutions of **72** in each of the various media were prepared and aliquots were extracted from each solution at various time points and subjected to HPLC analysis to measure the remaining intact enamine (Figure 9).

In two different standard assay media (MEM and RPMI, Figure 9A) enamine **72** was degraded at a similar rate. Addition of 10% foetal calf serum (FCS) to the media caused no difference in the observed stability, suggesting that breakdown was independent of the composition of the media and was non-enzymatic. The lack of enzymatic involvement in the breakdown was confirmed by incubation of **72** in protein solutions (Figure 9B). Similar rates of decomposition of **72** were observed in media containing FCS and water containing FCS, both of which contained active enzymes, compared with solutions of boiled (inactivated) FCS in water. A surprising result was that **72** was relatively stable over a period of 24 hours in human plasma, potentially reflecting the impact of reversible plasma protein binding. Whilst, the enamine **72** was stable in DMSO solutions for over 24 h and relatively stable at pH 2.2, under neutral (pH 7) and basic (pH 10.4) conditions the enamine **72** was rapidly degraded (Figure 9C), contrary to the expected profile. Together, these stability observations suggest that the enamine motif is relatively unstable under model conditions, which are similar to those used for the cell-based assays. This may explain the mostly flat SARs observed in cellular assays for this series, perhaps due to the enamine group degradation to a reactive aldehyde, which is a hydrolysis product common to all members of this series. Some notable differential activity is noted, however, and may be attributed to varying rates of enamine hydrolysis to the corresponding aldehyde more or less quickly than **72**. Reduction of the enamine to the corresponding tertiary amine may provide a possible solution to stability problems.

#### Structural insights into Nek2 activation

**Figure 10:**
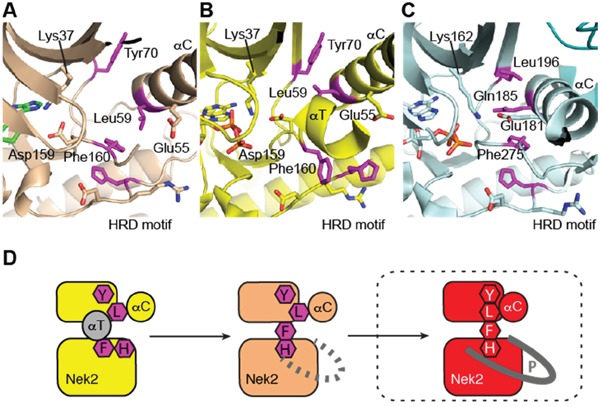
Purine ATP-competitive inhibitors induce a pre-active, DFG-in, αC-out conformation of Nek2 **A**. Crystal structure of Nek2 in complex with compound **11**. **B**. Crystal structure of Nek2 in complex with ADP (PDB code 2W5A). **C**. Crystal structure of active Aurora-A (PDB code 2W5A). In panels A-C, hydrophobic R-spine residues are coloured magenta. D. Schematic illustration of activation pathway of Nek2.

To gain an insight in to the molecular mechanisms of Nek2 activation, structural biology studies were conducted using Nek2 in complex with carboxamide **11**, which was one of the newly synthessied inhibitors with the greatest Nek2-selectivity profile (Table 2). In complex with purine-based ATP-competitive inhibitors, it has been previously shown that Nek2 adopts an inactive conformation characterized by a partially disordered activation loop and an outward position of the αC-helix (Figure 10A). This is similar to that observed in previously determined Nek2 structures such as the complex with ADP (Figure 10B) [34]. Here, in complex with carboxamide **11**, and in contrast to previous structures, the activation loop of Nek2 has a DFG-in conformation, indicating that **11** is the first reported inhibitor of Nek2 to function in this way. The phenylalanine (Phe-160 in Nek2) of the DFG motif is one of the four regulatory (R-) spine residues, which adopt a continuous hydrophobic column in active kinases (Figure 10C) [35]. The DFG-in conformation is required to form the R-spine. The HRD motif also adopts the position expected in an active kinase structure and thus, in the presence of **11**, Nek2 has a more ordered R-spine. However, one of the four R-spine residues is out of position compared to that expected in an active kinase because the αC-helix is in an αC-out position.

The conformation adopted here by Nek2 appears to be stabilized by **11**. The clearest connection here is the interaction of the ligand with Lys-37, which is connected to the DFG motif through an interaction with the side chain of Asp-159. The conformations of the DFG and HRD motifs are coupled through aromatic stacking between the side chains of Phe-160 and His-139. Thus, the purine scaffold induces a specific conformation of key structural elements of Nek2 through a network of H-bond interactions.

The crystal structure of Nek2 bound to purine compounds suggests an intermediate state of a kinase poised for activity, and we propose a stepwise pathway for Nek2 activation (Figure 10D). Unphosphorylated Nek2 is in an autoinhibited state, in which the activation loop forms an α-helix (αT) that stabilizes the outwards position of the αC-helix and blocks formation of an R-spine. Refolding of αT by formation of a DFG-in conformation of the activation loop is coupled to changes in the HRD motif that results in a partially formed R-spine and a disordered activation loop. In this pre-active state the outward location of the αC-helix is destabilized and the activation loop is disordered. The kinase may have sufficient flexibility to transiently adopt an active state necessary for autophosphorylation. Nek2 autophosphorylation is expected to result in a fully-active conformation of the kinase with a fully-assembled R-spine and an ordered activation loop. In our study, the pre-active state of Nek2 was induced by ATP-competitive inhibitors based on a purine scaffold (*i.e*. carboxamide **11**). In a physiological context, it is likely that Nek2 activation is promoted by protein-protein interactions, such as Nek2 dimerization.

**Figure 11:**
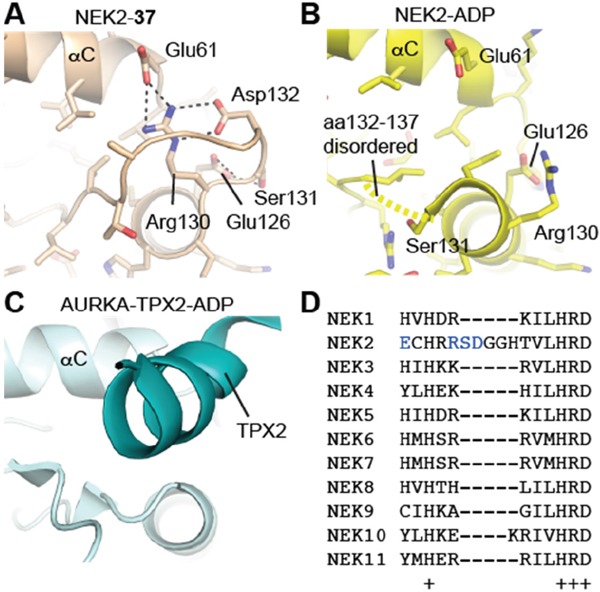
Nek2 has a unique insertion N-terminal to the ‘HRD’ motif **A**. Crystal structure of Nek2 in complex with compound **11**. **B**. Crystal structure of Nek2 in complex with ADP (PDB code 2W5A). **C**. Crystal structure of active Aurora-A (PDB code 2W5A). **D**. Sequence alignment of human Neks in the vicinity of the ‘HRD’ motif, showing the insert sequence that is unique to Nek2.

In crystal structures of Nek2 bound to purine inhibitors, the region C-terminal to the αE helix and N-terminal to the HRD motif is ordered (Figure 11A), unlike other structures of Nek2 (Figure 11B). The loop structure contacts the αC-helix in the N-lobe of the protein and occupies the equivalent space as the activatory helix of TPX2 in the Aurora-A/TPX2 complex (Figure 11C). Based on these observations, we predict that this loop will fulfill a similar role in the stabilization of the activation loop in the active Nek2 structure. This loop is not present in other Neks and is indeed unique to Nek2 among human kinases (Figure 11D). Importantly, targeting this unique protein conformation with small molecules presents an attractive opportunioty for selective inhibition of Nek2 over other kinome members.

In conclusion, a number of key structural requirements for selective Nek2 inhibition over CDK2 have been elucidated by synthesis and evaluation of purines probes bearing informative modifications, specifically at the 2-arylamino and 6-positions. These significantly include the importance of a terminal basic group, especially the NMe_2_ residue, on the *meta*-substituent of 2-arylamino derivatives which consistently showed good potency when incorporated into (homo) sulfonamide, (homo) carboxamide and urea sidechains. It was also noteworthy that a hydrogen bond donor-acceptor group, *e.g*. urea, played a role in potent Nek2 inhibition, whilst sulfonamides may confer CDK2 inhibitory activity due to interactions previously reported [32]. It may also be concluded that *para*-substitution on the 2-arylamino ring may be preferable for CDK2 inhibition, whereas *meta*-substitution generally affords lower CDK2 inhibitory activities, and in some cases improved Nek2 inhibition.

Aided by structural biology, we have shown that it may be possible to obtain further selectivity by removal of the *O*^6^-cyclohexylmethyl group, which abolished CDK2 inhibition but was not essential for Nek2 inhibition. A facile and rapid synthesis of 6-(dialkylaminovinyl) purines was described and such groups at the purine 6-position conferred potent Nek2 inhibition and a degree of kinase selectivity. Subsequently, it has been found that the precursor of these enamines, 6-ethynyl-2-phenylaminopurine **68**, reacts covalently with Cys-22 of Nek2, thus behaving as an irreversible inhibitor. Identification and structure-activity relationships for 2-arylamino-6-ethynylpurines as irreversible inhibitors of Nek2 kinase will be the subject of our next publication.

Crystal structures of Nek2 in complex with purines *e.g*. carboxamide **11** provide a snapshot of Nek2 in a conformation closer to that of an active kinase than was observed in previous Nek2 structures. This provides a potentially unique and selective molecular target for small molecule kinase inhibition. Moving forward, the development of tool compounds with improved potency and selectivity will be required in future studies to assist with target validation, cellular studies and to understand better the role of Nek2 in mitosis and cancer.

## MATERIALS AND METHODS

### Synthetic chemistry

All chemicals were purchased from standard suppliers. Solvents were purified and stored according to standard procedures. Melting points were obtained on a Stuart Scientific SMP3 apparatus and are uncorrected. TLC was performed with Merck 60 F254 silica gel plates. Where appropriate, compound mixtures were separated and purified using either medium pressure (‘flash’) chromatography, employing Davisil silica 40-60 μm, or using a Biotage SP4 automated chromatography system with UV monitoring at 254 and 290 nm. When using the Biotage SP4 to purify samples, the stationary phase was KP-SIL (silica), Biotage KP-NH or KP-C18 as appropriate. 12+M, 25+M or 40+M pre-packed columns were used as required. KP-NH was used for the normal-phase purification of polar organic amines. KP-C18 (18% carbon by weight) was used for the separation of polar and ionisable organic compounds, requiring a water-based eluent and lipophilic stationary phase. ^1^H NMR and ^13^C NMRspectra were recorded on a Bruker Spectrospin AC 300E spectrometer (300 MHz for ^1^H, 75 MHz for ^13^C) or a Bruker AMX (500 MHz for ^1^H, 126 MHz for ^13^C). Samples were acquired in deuterated solvents including CDCl_3_ and DMSO-*d*_6_. Where appropriate (compounds **69**-**73**), reactions were carried out with microwave heating, in sealed vessels, using a Biotage Initiator reactor equipped with a ‘Sixty robot’. Samples were irradiated at 2.45 GHz, reaching temperatures from 60-250°C (rate of heating 2-5°C/sec) and pressures up to 20 bars. LCMS was carried out on either a Micromass Platform instrument operating in positive and negative ion electrospray mode, employing a 50 × 4.6 mm C18 column (Supelco Discovery or Waters Symmetry) and a 15 min gradient elution of 0.05% formic acid and methanol (10-90%), or on a Finnegan LCQ instrument in positive ion mode with a Phenomenex 5μ Luna C18 column, 4.6 mm x 50 mm and an 8 min gradient of 0.1% aqueous formic acid and acetonitrile (5-98%), with a flow rate of 2 mL/min. IR spectra were recorded on a Bio-Rad FTS 3000MX diamond ATR. HRMS were measured using a Finnigan MAT 95 XP or a Finnigan MAT 900 XLT by the EPSRC National Mass Spectrometry Service Centre (Swansea).

#### Synthesis of 2-arylaminopurines (compounds 8, 10-26, 59-61, 65, 66). Method I. General Procedure

To a stirred suspension of the appropriate 2-fluoro-9*H*-purine (0.49 mmol) and the required aniline derivative (0.98 mmol) in TFE (25 mL/g of fluoropurine) was added TFA (0.19 mL, 2.46 mmol) dropwise. The resulting solution was heated under reflux for 48 h under a nitrogen atmosphere. The solvent was removed *in vacuo* and the residue was redissolved in EtOAc (10 mL). The solution was washed several times with saturated NaHCO_3_ solution (3 × 10 mL), and the aqueous extracts were combined and washed with EtOAc (10 mL). The combined organic layers were dried (Na_2_SO_4_) and the solvent was removed to give a residue that was purified as indicated.

#### 3-(6-Cyclohexylmethoxy-9*H*-purin-2-ylamino)benzamide (8)

The title compound was synthesised according to Method I using 2-fluoro-6-cyclohexylmethoxypurine (**9**, 125 mg, 0.50 mmol) and 3-aminobenzamide (136 mg, 1.0 mmol), TFE (5 mL), and TFA (0.19 mL, 2.5 mmol). The crude product was dry-loaded onto silica (~30 mL) and purified by column chromatography (eluent: EtOAc) to give a light brown powder, (62 mg, 34%): *R_f_* = 0.18 (MeOH-EtOAc; 1:9); mp 231-232°C; IR (cm^−1^) 3350, 2922, 2851, 2160, 1577, 1541, 1118; ^1^H NMR (300 MHz, DMSO-*d_6_*) δ 0.85-1.70 (11H, m, cyclohexyl), 4.36 (2H, d, *J* = 5.4 Hz, OCH2), 7.24-7.34 (2H, m, 2 × ArH), 7.39 (1H, d, *J* = 8.4 Hz, ArH), 7.85 (2H, s, CONH_2_), 7.99 (1H, s, H-8),8.37 (1H, s, ArH), 9.43 (1H, s, ArNHAr), 12.83 (1H, s br, N9-H); ^13^C NMR (75 MHz, DMSO-*d_6_*) δ 25.6, 26.4, 29.6, 37.4, 71.6, 97.0, 114.4, 118.7, 120.2, 121.6, 128.4, 135.5, 141.9, 155.8, 169.0; LCMS (ES^+^) *m/z* 367 [M+H]^+^; HRMS (ES^+^) calcd for C_19_H_23_N_6_O_2_ [M+H]^+^ 367.1877, found 367.1875; λ_max_ (EtOH) 273, 293 nm.

#### 3-(6-Cyclohexylmethoxy-9*H*-purin-2-ylamino)-N-methylbenzamide (10)

The title compound was synthesised according to Method I using 2-fluoro-6-cyclohexylmethoxypurine (**9**, 75 mg, 0.30 mmol), 3-amino-*N*-methylbenzamide (see ESI **S3**; 102 mg, 0.68 mmol), TFE (3 mL), and TFA (0.11 mL, 1.5 mmol). The crude product was allowed to stand in DCM (20 mL) for 2 h at room temperature. The resulting precipitate was filtered and washed with DCM (2 × 5 mL) to give the title compound as a pale pink solid (88 mg, 77%): *R_f_* = 0.10 (EtOAc); mp 252-254°C; IR (cm^−1^) 3257, 3061, 2928, 2854, 1638, 1612, 1579, 1538, 1485, 1446, 1381, 1257; ^1^H NMR (300 MHz, DMSO-*d*_6_) δ 1.0–1.84 (11H, m, cyclohexyl), 2.77 (3H, d, *J* = 4.4 Hz, NHC*H*_3_), 4.35 (2H, d, *J* = 6.1 Hz, OCH_2_), 7.32 (2H, m, 2 × ArH), 7.85 (1H, br, CONH), 7.98 (1H, s, Ar*H*), 8.27-8.35 (2H, m, Ar*H* and H-8), 9.45 (1H, s, ArN*H*Ar), 12.83 (1H, s, N9-H); LCMS (ES^+^) *m/z* 381.45 [M+H]^+^; HRMS (ES^+^) calcd for C_20_H_25_N_6_O_2_ [M+H]^+^ 381.2034, found 381.2034; λ_max_ (EtOH) 273 nm.

#### 3-(6-Cyclohexylmethoxy-9*H*-purin-2-ylamino)-N,N-dimethylbenzamide (11)

The title compound was synthesised according to Method I using 2-fluoro-6-cyclohexylmethoxypurine (**9**, 80 mg, 0.32 mmol), 3-amino-*N,N*-dimethylbenzamide (see ESI **S4**; 105 mg, 0.64 mmol), TFE (4 mL), and TFA (0.12 mL, 1.6 mmol). The crude product was dry-loaded onto silica and purified by column chromatography (EtOAc-petrol; 9:1) to give an off-white powder (68 mg, 54%): *R_f_* = 0.09 (EtOAc); mp 120-121°C; IR (cm^−1^) 3266, 2922, 2849, 1973, 1581, 1435, 1386; ^1^H NMR (300 MHz, DMSO-*d*_6_) δ 0.95-1.80 (11H, m, cyclohexyl), 2.95 (6H, s, N(CH_3_)_2_), 4.33 (2H, d, *J* = 6.3 Hz, OCH_2_), 6.90 (1H, d, *J* = 7.6 Hz, Ar*H*), 7.31 (1H, dd, *J* = 7.8, 8.0 Hz, ArH), 7.77 (1H, d, *J* = 8.1 Hz, ArH), 7.98 (1H, s, Ar*H*), 8.03 (1H, s, H-8), 9.45 (1H, s, ArNHAr), 12.91 (1H, s, N9-H); ^13^C NMR (75 MHz, DMSO-*d*_6_) δ 25.6, 26.4, 29.6, 37.3, 71.5, 114.1, 116.93, 119.3, 119.4, 128.6, 137.3, 140.2, 141.6, 155.6, 160.0, 170.8; LCMS (ES^+^) *m/z* 395 [M+H]^+^; HRMS (ES^+^) calcd for C_21_H_27_N_6_O_2_ [M+H]^+^ 395.2190, found 395.2189; λ_max_ (EtOH) 272, 295 nm.

#### 3-(6-Cyclohexylmethoxy-9*H*-purin-2-ylamino)-*N,N*-dimethylthiobenzamide (12)

The title compound was synthesised according to Method I using 2-fluoro-6-cyclohexylmethoxypurine (**9**, 88 mg, 0.35 mmol), 3-amino-*N,N*-dimethylthiobenzamide (see ESI **S7**; 140 mg, 0.78 mmol), TFE (3 mL), and TFA (0.13 mL, 1.8 mmol). The crude product was dry-loaded onto silica (4 mL) and purified by column chromatography (EtOAc-petrol; 7:3) followed by further purification by semi-prep HPLC (mobile phase A – eluted with 40% acetonitrile, flow-rate 12.75 mL/min, wavelength 280 nm) to give the title compound as a yellow solid (35 mg, 24%): *R_f_* = 0.18 (EtOAc-petrol; 7:3); mp 231-232°C (dec.); IR (cm^−1^) 2926, 2851, 1629, 1580, 1521, 1487, 1431, 1393, 1258, 1116; ^1^H NMR (300 MHz, DMSO-*d*_6_) δ 0.9-1.7 (11H, m, cyclohexyl), 3.17 (3H, s, NCH_3_), 3.50 (3H, s, NCH_3_), 4.33 (2H, d, *J* = 6.1 Hz, OCH_2_), 6.78 (1H, d, *J* = 7.4 Hz, Ar*H*), 7.26 (1H, dd, *J* = 7.9, 7.9 Hz, Ar*H*), 7.66 (1H, d, *J* = 7.7 Hz, Ar*H*), 7.90 (1H, s, Ar*H*), 8.01 (1H, s, H-8), 9.45 (1H, s, ArN*H*Ar), 12.72 (1H, s br, N9-H); LCMS (ES^+^) *m/z* 411 [M+H]^+^; HRMS (ES^+^) calcd for C_21_H_27_N_6_OS [M+H]^+^ 411.1962, found 411.1959; λ_max_ (EtOH) 236, 277 nm.

#### 3-(6-Cyclohexylmethoxy-9*H*-purin-2-ylamino)thiobenzamide (13)

The title compound was synthesised according to Method I using 2-fluoro-6-cyclohexylmethoxypurine (**9**, 75 mg, 0.3 mmol), 3-aminothiobenzamide (see ESI **S8**; 92 mg, 0.6 mmol), TFE (3 mL), and TFA (0.11 mL, 1.5 mmol). The crude product was dry-loaded onto silica (4 mL) and purified by column chromatography (EtOAc-petrol; 7:3 up to EtOAc) followed by further purification by semi-prep HPLC (5 → 100% *v/v* acetonitrile: water: NH_4_OH over 25 min; flow-rate 12.75 mL/min, wavelength 280 nm) to give the title compound as a yellow solid (37 mg, 32%): *R_f_* = 0.28 (EtOAc-petrol; 9:1); mp 148-149°C (dec.); IR (cm^−1^) 3271, 3077, 2920, 2849, 2363, 2337, 1597, 1537, 1483, 1438, 1391, 1352, 1319, 1283, 1117, 973; ^1^H NMR (300 MHz, DMSO-*d*_6_) δ 1.00-1.90 (11H, m, cyclohexyl), 4.37 (2H, d, *J* = 5.8 Hz, OCH_2_), 7.29 (2H, d, *J* = 7.3 Hz, Ar*H*), 7.79 (1H, d, *J* = 7.3 Hz, Ar*H*), 7.98 (1H, s, H-8), 8.44 (1H, s, Ar*H*), 9.43 (2H, br, NH_2_), 9.82 (1H, s, ArNHAr), 12.81 (1H, s br, N9-H); ^13^C NMR (125 MHz, DMSO-*d*_6_) δ 25.2, 26.0, 29.2, 36. 9, 71.2, 114.9, 118.2, 118.8, 120.9, 127.8, 139.0, 140.7, 140.7, 154.2, 155.3, 160.1, 201.1; LCMS (ES^+^) *m/z* 411 [M+H]^+^; HRMS (ES^+^) calcd for C_19_H_23_N_6_OS [M+H]^+^ 383.1649, found 383.1647; λ_max_ (EtOH) 278, 295 nm.

#### 3-(6-Cyclohexylmethoxy-9*H*-purin-2-ylamino)-*N,N*-dimethylbenzenesulfonamide (14)

The title compound was synthesised according to Method I using 2-fluoro-6-cyclohexylmethoxypurine (**9**,75 mg, 0.30 mmol), 3-amino-*N,N*-dimethylbenzenesulfonamide (see ESI **S10**; 136 mg, 0.68 mmol), TFE (3 mL), and TFA (0.11 mL, 1.5 mmol). After concentration *in vacuo*, the residual oil was extracted into DCM (10 mL) and cooled to 0°C overnight. The resulting precipitate was collected by filtration under vacuum, washed with DCM (10 mL) and dried (Na_2_SO_4_) to give the title compound as an off-white solid (35 mg, 27%): *R_f_*= 0.54 (EtOAc); mp 143-145°C; IR (cm^−1^) 2926, 2850, 1598, 1579, 1543, 1433, 1394, 1334, 1249, 1149, 951; ^1^H NMR (300 MHz, DMSO-*d_6_*) δ 1.2-1.9 (11H, m, cyclohexyl), 2.64 (6H, s, N(CH_3_)_2_, 4.36 (2H, d, *J* = 6.2 Hz, OCH_2_), 7.24 (1H, d, *J* = 7.5 Hz, Ar*H*), 7.52 (1H, dd, *J* = 8.0, 8.0 Hz, Ar*H*), 7.96 (1H, d, *J* = 7.4 Hz, Ar*H*), 8.04 (1H, s, H-8), 8.50 (1H, s, Ar*H*), 9.77 (1H, s, ArNHAr), 12.89 (1H, s, N9-H); ^13^C NMR (125 MHz, DMSO-*d_6_*) δ 25.2, 26.0, 29.2, 36.9, 37.7, 71.3, 116.2, 119.1, 122.0, 129.3, 134.9, 142.0, 154.9; LCMS (ES^+^) *m/z* 431 [M+H]^+^; Anal. calcd for C_20_H_26_N_6_O_3_: C, 55.80; H, 6.09; N, 19.52%; found: C, 55.77; H, 6.00; N, 19.47; λ_max_ (EtOH) 275, 293 nm.

#### [3-(6-Cyclohexylmethoxy-9*H*-purin-2-ylanilino]-*N*-methylacetamide (15)

The title compound was synthesised according to Method I using 2-fluoro-6-cyclohexylmethoxypurine (**9**, 75 mg, 0.30 mmol), 2-(3-aminophenyl)-*N*-methylacetamide (see ESI **S12**; 98 mg, 0.60 mmol), TFE (3 mL), and TFA (0.11 mL, 1.5 mmol). The crude product was adsorbed onto silica gel (~3 mL) and purified by column chromatography (eluent: 5% MeOH/ EtOAc) and HPLC to give an off-white powder (53 mg, 45%): *R_f_* = 0.23 (MeOH-EtOAc; 1:9); mp 176-177°C; IR (cm^−1^) 3267, 2921, 2850, 2362, 2160, 2013, 1587, 1499, 1396, 1346, 1239, 1119; ^1^H NMR (300 MHz, DMSO-*d_6_*) δ 1.0–1.8 (11H, m, cyclohexyl), 2.50 (3H, d, *J* = 4.6 Hz, NCH_3_), 4.26 (2H, d, *J* = 6.3 Hz, OCH_2_), 6.74 (1H, d, *J =* 7.6 Hz, Ar*H*), 7.10 (1H, dd, *J* = 7.8, 7.9 Hz, Ar*H*), 7.56 (1H, s, Ar*H*), 7.62 (1H, d, *J* = 8.2 Hz, Ar*H*), 7.85 (1H, q, *J* = 4.6 Hz, CONH), 7.96 (1H, s, H-8), 9.21 (1H, s, ArNHAr), 12.68 (1H, br, N9-H); ^13^C NMR (75 MHz, DMSO-*d_6_*) δ 25.6, 26.0, 26.4, 29.6, 37.3, 43.1, 44.5, 71.5, 117.2, 119.9, 122.0, 128.4, 134.6, 136.8, 139.8, 141.4, 156.0, 170.8; LCMS (ES^+^) *m/z* 395 [M + H]^+^; Anal. calcd for C_21_H_26_N_6_O_2_: C, 63.94; H, 6.64; N, 21.31%; found: C, 63.80; H, 6.85; N, 20.97; λ_max_ (EtOH) 207.0, 271.5, 293.5 nm.

#### 1-(4-(6-(Cyclohexylmethoxy)-9*H*-purin-2-ylamino)phenyl)-3-(2-morpholinoethyl)urea (16)

Following Method I, the title compound was prepared using 6-(cyclohexylmethoxy)-2-fluoro-9*H*-purine (**9**) (0.15 g, 0.59 mmol) and 1-(4-aminophenyl)-3-(2-morpholinoethyl) urea (see ESI **S18**; 0.31 g, 1.17 mmol) and TFA (0.27 mL, 3.5 mmol) in TFE (6.0 mL). The compound was purified by recrystallisation from EtOAc to obtain a white solid (50 mg, 17%): mp 161-163°C; IR (cm^−1^) 3134, 2849, 2812, 1634 υ(NN'C=O), 1558, 1506; ^1^H NMR (300 MHz, DMSO-*d_6_*) δ 1.12-1.42 (5H, m, cyclohexyl), 1.78-1.99 (6H, m, cyclohexyl), 2.54 (6H, m, N(CH_2_)_3_), 3.38 (2H, dt, *J =* 6.0, 12.0 Hz, CH_2_), 3.73 (4H, t, *J =* 4.5 Hz, OCH_2_ morpholine), 4.37 (2H, d, *J =* 6.0 Hz, OCH_2_), 7.21 (2H, d, *J =* 8.5 Hz, H-2′ and H-6′), 7.67 (2H, d, *J =* 8.5 Hz, H-3′ and H-5′), 7.92 (1H, s, H-8); ^13^C NMR (75 MHz, DMSO-*d_6_*) δ 27.3, 28.1, 31.2, 36.4, 39.1, 54.1, 59.4, 68.1, 73.3, 115.1, 121.4, 121.6, 123.0, 135.2, 137.9, 151.1, 151.9, 158.0, 159.1; LCMS (ES^+^) *m/z* 495.1 [M+H]^+^; λ_max_ (EtOH) 280.0, 238.0 nm.

#### 1-(4-(6-(Cyclohexylmethoxy)-9*H*-purin-2-ylamino)phenyl)-3-(2-(piperidin-1-yl)ethyl)urea (17)

Following Method I, the title compound was prepared using 6-(cyclohexylmethoxy)-2-fluoro-9*H*-purine (**9**, 0.15 g, 0.59 mmol) and 1-(4-aminophenyl)-3-(2-(piperidin-1-yl)ethyl)urea (see ESI **S20**; 0.30 g, 1.17 mmol) and TFA (0.27 mL, 3.5 mmol) in TFE (4 mL). The compound was purified using the Biotage SP4 chromatography (KP-NH; MeOH-EtOAc; 1:9) to obtain the desired compound as an off-white solid (0.14 g, 48%): mp 158-160°C; IR (cm^−1^) 2924, 2849, 1644 υ(NN'C=O), 1610, 1556, 1506; ^1^H NMR (300 MHz, DMSO-*d_6_*) δ 1.10-1.19 (5H, m, cyclohexyl), 1.48-1.55 (6H, m, CH_2_ piperidine), 1.76-1.84 (6H, m, cyclohexyl), 3.17 (2H, m, CH_2_), 2.33 (6H, m, N(CH_2_)_3_), 4.30 (2H, d, *J =* 6.0 Hz, OCH_2_), 7.27 (2H, d, *J =* 8.0 Hz, H-2′ and H-6′), 7.62 (2H, d, *J =* 8.0 Hz, H-3′ and H-5′), 7.95 (1H, s, H-8), 8.47 (1H, s, NH-4′), 9.09 (1H, s, NH); ^13^C NMR (125 MHz, DMSO-*d_6_*) δ 24.1, 25.2, 25.5, 26.0, 29.2, 36.4, 36.8, 54.0, 58.2, 70.9, 118.1, 119.2, 119.3, 128.4, 134.3, 134.9, 138.5, 154.3, 155.3, 155.7, 160.3; LCMS (ES^+^) *m/z* 493.6 [M+H]^+^; HRMS (ES^+^) calcd for C_26_H_36_N_8_O_2_ [M+H]^+^ 493.3034, found 493.3040; λ_max_ (EtOH) 281.5, 238.0 nm.

#### 1-(4-(6-(Cyclohexylmethoxy)-9*H*-purin-2-ylamino)phenyl)-3-(3-(dimethylamino)propyl)urea (18)

Following Method I, the title compound was prepared using 6-(cyclohexylmethoxy)-2-fluoro-9*H*-purine (**9**) (0.15 g, 0.59 mmol) and 1-(4-aminophenyl)-3-(3-(dimethylamino)propyl)urea (see ESI **S22**; 0.30 g, 1.17 mmol) and TFA (0.27 mL, 3.5 mmol) in TFE (4 mL). The compound was purified using the Biotage SP4 chromatography (KP-NH; MeOH-EtOAc; 1:4) to obtain title compound as an off-white solid (94 mg, 34%): mp 110-112°C; IR (cm^−1^) 2925, 2855, 2127, 2050, 1672 υ(NN'C=O), 1614, 1551, 1508; ^1^H NMR (300 MHz, DMSO-*d_6_*) δ 1.07-1.21 (5H, m, cyclohexyl), 1.50-1.57 (2H, quin., *J* = 7.0 Hz, CH_2_), 1.69-1.75 (6H, m, cyclohexyl), 2.12 (6H, s, N(CH_3_)_2_), 2.22 (2H, t, *J =* 7.0 Hz, CH_2_), 3.09 (2H, dt, *J* = 5.5, 7.0 Hz, CH_2_), 4.30 (2H, d, *J =* 6.0 Hz, OCH_2_), 6.15 (1H, br t, *J =* 5.5 Hz, NH), 7.27 (2H, d, *J =* 8.5 Hz, H-2′ and H-6′), 7.62 (2H, d, *J =* 8.5 Hz, H-3′ and H-5′), 7.96 (1H, s, H-8), 8.34 (1H, s, NH-4′), 9.08 (1H, s, NH); ^13^C NMR (125 MHz, DMSO-*d_6_*) δ 25.2, 26.0, 27.8, 29.2, 36.8, 37.5, 45.2, 56.8, 70.9, 118.2, 119.2, 134.3, 134.9, 138.9, 155.5, 155.7, 158.1; LCMS (ES^+^) *m/z* 467.7 [M+H]^+^; HRMS (ES^+^) calcd for C_24_H_35_N_8_O_2_ [M+H]^+^ 467.2877, found 467.2870; λ_max_ (EtOH) 279.0, 238.0 nm.

#### 6-(Cyclohexylmethoxy)-*N*-(4-nitrophenyl)-9*H*-purin-2-amine (19)

The title compound was prepared according to Method I using: 6-(cyclohexylmethoxy)-2-fluoro-9*H*-purine (**9**, 2.00 g, 8.0 mmol), 4-nitroaniline (2.21 g, 16.0 mmol) and TFA (3.06 mL, 40.0 mmol) in TFE (50 mL). The crude orange solid was purified by recrystallisation from EtOAc and obtained as a yellow solid (2.18 g, 74%): mp 180-181°C; IR (cm^−1^) 3390, 3331, 2936, 2862, 2758, 1728, 1591, 1522 υ(strong, br, NO_2_ asymmetric stretch), 1330 υ(strong, sharp, NO_2_ symmetric stretch); ^1^H NMR (300 MHz, DMSO-*d_6_*) δ 1.02-1.29 (5H, m, cyclohexyl), 1.66-1.85 (6H, m, cyclohexyl), 4.32 (2H, d, *J* = 6.0 Hz, OC*H*_2_), 8.04 (2H, d, *J* = 9.0, H-2′ and H-6′), 8.18 (2H, d, *J* = 9.0, H-3′ and H-5′), 8.21 (1H, s, H-8); ^13^C NMR (75 MHz, DMSO-*d_6_*) δ 21.0, 25.4, 26.5, 30.1, 71.9, 117.5, 125.5, 140.2, 148.2, 154.6; LCMS (ES^+^) *m/z* 369.3 [M+H]^+^; HRMS (ES^+^) calcd for C_18_H_20_N_6_O_3_ [M+H]^+^ 369.3614, found 369.3611.

#### 6-(Cyclohexylmethoxy)-*N*-(3-nitrophenyl)-9*H*-purin-2-amine (20)

According to Method I, the title compound was prepared using: 6-(cyclohexylmethoxy)-2-fluoro-9*H*-purine (**9**) (2.00 g, 8.0 mmol), 3-nitroaniline (2.21 g, 16.0 mmol) and TFA (3.06 mL, 40.0 mmol) in TFE (50 mL). The crude orange solid was purified by column chromatography (silica; EtOAc-petrol; 4:6) and isolated as a yellow solid (1.89 g, 64%): mp 217-218°C; IR (cm^−1^) 3419, 3351, 2931, 2854, 2769, 1732, 1592, 1532 υ(strong, br, NO_2_ asymmetric stretch), 1351 υ(strong, sharp, NO_2_ symmetric stretch); ^1^H NMR (300 MHz, DMSO-*d_6_*) δ 1.17 (5H, m, cyclohexyl), 1.77 (6H, m, cyclohexyl), 4.38 (2H, d, *J =* 6.0 Hz, OCH_2_), 7.53 (1H, dd, *J =* 7.5, 8.0 Hz, H-5′), 7.73 (1H, dd, *J* = 1.5, 2.0 Hz, H-2′), 7.94-8.11 (2H, m, H-4′, H-6′), 8.29 (1H, s, H-8), 9.10 (1H, s, N-2 H); LCMS (ES^+^) *m/z* 369.4 [M+H]^+^; HRMS (ES^+^) calcd for C_18_H_20_N_6_O_3_ [M+H]^+^ 369.3614, found 369.3609; λ_max_ (EtOH) 379, 292, 271, 227.5 nm.

#### Reduction of aryl-nitro groups to corresponding anilines (compounds 21 and 22)

To a stirred solution of the nitroaromatic compound in sufficient anhydrous solvent as indicated was added 10% palladium on activated carbon (30% *w/w*). The resulting mixture was stirred under an atmosphere of H_2_ at room temperature for 24 h. The reaction mixture was filtered through a bed of Celite eluting with a mixture of MeOH-DCM (1:9), to afford the title compound following removal of the solvent *in vacuo*.

#### 2-(4-Aminophenyl)amino-6-cyclohexylmethoxy-purine (21)

The title compound was synthesised from 6-(cyclo hexylmethoxy)-*N*-(4-nitrophenyl)-9*H*-purin-2-amine (**19**, 1.20 g, 3.26 mmol) with 10% palladium on activated carbon (0.36 g) in THF (100 mL) to obtain the title compound as a beige solid (1.10 g, 100%): *R_f_*= 0.30 (MeOH-DCM; 1:9); mp 228-230°C; IR (cm^−1^) 3431 υ(NH), 3370 υ(NH_2_), 3234, 2923, 2834, 2359, 1613, 1584; ^1^H NMR (300 MHz, DMSO-*d_6_*) δ 1.02-1.29 (5H, m, cyclohexyl), 1.69-1.85 (6H, m, cyclohexyl), 4.26 (2H, d, *J* = 6.0 Hz, OCH_2_), 4.72 (2H, br s, NH_2_), 6.50 (2H, d, *J* = 8.5 Hz, H-2′ and H-6′), 7.35 (2H, d, *J* = 8.5 Hz, H-3′ and H-5′), 7.99 (1H, s, H-8), 9.01 (1H, s, NH); ^13^C NMR (75 MHz, DMSO-*d_6_*) δ 25.2, 26.5, 29.6, 37.2, 71.1, 113.8, 121.3, 130.2, 143.7, 156.6; LCMS (ES^+^) *m/z* 339.1 [M+H]^+^; HRMS (ES^+^) calcd for C_18_H_22_N_6_O [M+H]^+^ 389.1928, found 389.1931; λ_max_ (EtOH) 277.0, 240.0 nm.

#### 2-(3-Aminophenyl)amino-6-cyclohexylmethoxy-purine (22)

The title compound was synthesised from 6-(cyclo hexylmethoxy)-*N*-(3-nitrophenyl)-9*H*-purin-2-amine (**20**, 0.30 g, 0.82 mmol) with 10% palladium on activated carbon (90 mg) in THF (40 mL) to afford the title compound as a brown solid (0.23 g, 84%): mp 117-118°C (dec); IR (cm^−1^) 3254 υ(NH_2_), 2922, 2847, 1587, 1443, 1389, 1352, 1118; ^1^H NMR (300 MHz, DMSO-*d_6_*) δ 1.38-1.87 (5H, m, cyclohexyl), 1.72 (6H, m, cyclohexyl), 4.31 (2H, d, *J =* 6.0 Hz, OCH_2_), 4.89 (2H, br s, NH_2_), 6.18 (1H, dd, *J =* 7.5, 8.0 Hz, H-5′), 6.95 (3H, m, H-2′, H-4′, H-6′), 7.95 (1H, s, H-8), 8.96 (1H, s, NH); ^13^C NMR (125 MHz, DMSO-*d_6_*) δ 25.2, 26.0, 29.2, 36.9, 70.9, 104.7, 107.3, 107.7, 114.5, 128.5, 138.6, 141.5, 148.6, 155.7, 160.0; LCMS (ES^+^) *m/z* 339.0 [M+H]^+^; HRMS (ES^+^) calcd for C_18_H_22_N_6_O [M+H]^+^ 389.1928, found 389.1934; λ_max_ (EtOH) 301, 271, 224.5 nm.

#### Synthesis of aminophenylacetic acid derivatives (23 – 25)

To a mixture of the aniline (2.25 mol. equiv.) and (**9**, 1 mol. equiv.) in TFE (5 mL/mmol) was added TFA (5 mol. equiv.). The mixture was boiled at reflux for 24 h and allowed to cool to room temperature. After concentration *in vacuo*, THF (20 mL) and NaOH aqueous solution (1 M, 15 mL) were added to the residue and the resulting mixture was stirred overnight. The pH was adjusted to around 1 with conc. HCl and the product was extracted with EtOAc (250 mL). The organic phase was separated, washed with 10 % HCl solution and dried (NaSO_4_). Removal of the solvent gave crude product, to which Et_2_O (100 mL) was added. After allowing the mixture to stand for 3 h the resulting precipitate was collected by suction filtration and washed with diethyl ether (30 mL). Recrystallisation from MeOH gave the pure product.

#### 3-(6-Cyclohexylmethoxy-9*H*-purin-2-ylamino)benzoic acid (23)

The title compound was synthesised using 6-cyclohexylmethoxy-2-fluoro-9-methyl-9*H*-purine (**9**, 250 mg, 1.0 mmol), 3-aminobenzoic acid (309 mg, 2.3 mmol), TFE (5.0 mL), and TFA (0.37 mL, 5 mmol) to give an off-white solid (259 mg, 71%): *R_f_* = 0.18 (MeOH-EtOAc; 1:9); mp 182-184°C (dec.); IR (cm^−1^) 2925, 2850, 1593, 1564, 1440, 1357, 1114, 972; ^1^H NMR (300 MHz, DMSO-*d*_6_) δ 1.0-1.9 (11H, m, cyclohexyl), 4.36 (2H, d, *J* = 5.8 Hz, OCH_2_), 7.35 (1H, dd, *J* = 7.6, 7.8 Hz, Ar*H*), 7.49 (1H, d, *J* = 7.3 Hz, Ar*H*), 7.92 (1H, d, *J* = 7.6 Hz, Ar*H*), 8.03 (1H, s, Ar*H*), 8.55 (1H, s, H-8), 9.50 (1H, s, ArNHAr), 12.86 (1H, s br, N9-H); LCMS (ES^+^) *m/z* 368 [M+H]^+^; HRMS (ES^+^) calcd for C_19_H_22_N_6_O_3_ [M+H]^+^ 368.1717, found 368.1722; λ_max_ (EtOH) 225, 274 nm.

#### [3-(6-Cyclohexylmethoxy-9*H*-purin-2-ylamino)phenyl]acetic acid (24)

The title compound was synthesised using 6-cyclohexylmethoxy-2-fluoro-9-methyl-9*H*-purine (**9**, 2.0 g, 8.0 mmol), 3-aminophenylacetic acid (2.7 g, 18 mmol), TFE (20 mL), and TFA (3.0 mL, 40 mmol) to give a colourless solid (1.7 g, 55%): *R_f_* = 0.05 (MeOH-EtOAc; 0.5-9.5); mp 221-222°C (dec.); IR (cm^−1^) 3434, 3118, 2923, 2851, 1603, 1493, 1417, 1261; ^1^H NMR (300 MHz, DMSO-*d*_6_) δ 1.0-1.9 (11H, m, cyclohexyl), 3.51 (2H, s, ArCH_2_), 4.33 (2H, d, *J* = 6.2 Hz, OCH_2_), 6.79 (1H, d, *J* = 7.3 Hz, Ar*H*), 7.18 (1H, dd, *J* = 7.8, 7.9 Hz, Ar*H*), 7.65 (1H, d, *J* = 8.3 Hz, Ar*H*), 7.73 (1H, s, Ar*H*), 8.00 (1H, s, H-8), 9.29 (1H, s, ArNHAr), 12.29 (1H, s br, CO_2_H), 12.78 (1H, s br, N9-H); LCMS (ES^+^) *m/z* 382 [M+H]^+^; λ_max_ (EtOH) 209, 272 nm.

#### [3-(6-Cyclohexylmethoxy-9*H*-purin-2-ylamino)phenyl]propionic acid (25)

The title compound was synthesised using 6-cyclohexylmethoxy-2-fluoro-9-methyl-9*H*-purine (**9**, 300 mg, 1.2 mmol), 3-(3-aminophenyl)propionic acid (446 mg, 2.7 mmol), TFE (5.0 mL), and TFA (0.45 mL, 6.0 mmol) to give an off-white solid (225 mg, 48%): *R_f_* = 0.18 (MeOH-EtOAc; 1:9); mp 231-232°C (dec.); IR (cm^−1^) 2922, 2849, 1707, 1639, 1595, 1491, 1415, 1251, 1128, 972; ^1^H NMR (300 MHz, DMSO-*d*_6_) δ 1.0-1.9 (11H, m, cyclohexyl), 2.53 (2H, t, *J* = 7.9 Hz, C*H*_2_CO_2_H), 2.80 (2H, t, *J* = 7.7 Hz, ArCH_2_), 4.35 (2H, d, *J* = 6.3 Hz, OCH_2_), 6.81 (1H, d, *J* = 7.6 Hz, Ar*H*), 7.17 (1H, dd, *J* = 7.8, 7.9 Hz, Ar*H*), 7.58 (1H, d, *J* = 8.1 Hz, Ar*H*), 7.71 (1H, s, Ar*H*), 8.38 (1H, s, H-8), 9.41 (1H, s, ArNHAr), 12.10 (1H, s br, N9-H); ^13^C NMR (125 MHz, DMSO-*d_6_*) δ 21.1, 25.2, 25.9, 29.1, 30.7, 35.3, 36.8, 39.0, 71.4, 116.5, 118.5, 121.0, 128.3, 139.4, 140.7, 141.0, 154.3, 156.0, 159.0, 173.7; LCMS (ES^+^) *m/z* 396 [M+H]^+^; HRMS (ES^+^) calcd for C_21_H_26_N_5_O_3_ [M+H]^+^ 396.2030, found 396.2026; λ_max_ (EtOH) 272 nm.

#### 2,2,2-Trifluoroethyl-3-(6-cyclohexylmethoxy-9*H*-purin-2-ylamino)phenylmethanesulfonate (26)

ThThe title compound was synthesised according to Method I using 2-fluoro-6-cyclohexylmethoxypurine (9) (1.24 g, 5.0 mmol), 2,2,2-trifluoroethyl 3-aminophenylmethanesulfonate (S26, 2.8 g, 10 mmol), TFE (25 mL), and TFA (1.84 mL, 25 mmol). The crude product was adsorbed onto silica (~50 mL) and purified by chromatography (silica; EtOAc-petrol; 6:4) to give a viscous oil that was triturated with DCM (35 mL), to afford a pale yellow powder (1.83 g, 74%): *R_f_* = 0.22 (EtOAc-petrol; 6:4); mp 199-200°C; IR (cm^−1^) 3436, 3112, 2924, 2848, 1591, 1537, 1494, 1435, 1392, 1338, 1287, 1151, 1028; ^1^H NMR (300 MHz, DMSO-*d*_6_) δ 1.0-1.9 (11H, m, cyclohexyl), 4.35 (2H, d, *J* = 6.2 Hz, OCH_2_), 4.84 (2H, s, ArCH_2_), 4.94 (2H, q, *J* = 8.6 Hz, CH_2_CF_3_), 6.99 (2H, d, *J* = 7.6 Hz, 2 × Ar*H* overlap), 7.31 (1H, t, *J* = 7.8, 8.1 Hz, Ar*H*), 7.86-7.88 (2H, m, 2 × Ar*H*), 8.03 (1H, s, H-8), 9.44 (1H, s, ArNHAr), 12.86 (1H, br, N9-H); ^13^C NMR (125 MHz, DMSO-*d*_6_) δ 25.2, 26.0, 29.2, 36.9, 55.5, 55.7, 64.9 (q, ^2^*J*_C-F_ = 36 Hz), 71.2, 114.9, 118.8, 121.5, 122.6 (q, ^1^*J*_C-F_ = 276 Hz), 123.2, 128.6, 139.0, 141.4, 155.3, 160.1; ^19^F NMR (470 MHz, DMSO-*d_6_*) δ -72.86 (t, *J* = 8.6 Hz, CF_3_); LCMS (ES^+^) *m/z* 500 [M+H]^+^; Anal. calcd for C_21_H_24_F_3_N_5_O_4_S: C, 50.50; H, 4.84; N, 14.02%; found: C, 50.80; H, 4.56; N, 13.91; λ_max_ (EtOH) 272, 292 nm.

#### Synthesis of 4-*N*-acyl-(2—phenyl)-amino-9*H*-purines (compounds 27-30)

A stirred solution of the 4- (**21**) or 3-substituted aniline (**22**) (0.20 g, 0.59 mmol), 4-dimethylaminopyridine (0.07 g, 0.59 mmol) and Et_3_N (0.33 mL, 2.36 mmol) in THF (5 mL) was maintained at 0°C. To the chilled mixture was slowly added the required acyl chloride (1.77 mmol) and the reaction mixture was allowed to warm to room temperature (*or* heated to reflux as stated) with continued stirring for 18 h. EtOAc (20 mL) was added to the crude mixture and washed with sat. NaHCO_3_ solution (3 × 10 mL). The combined aqueous phase was re-extracted using EtOAc (2 × 10 mL). The combined organic extracts were washed with 0.1 M HCl (3 × 10 mL) and the acidic extracts again re-extracted with EtOAc (2 × 10 mL). The combined organic extracts were dried (Na_2_SO_4_). Products were obtained after purification as described. The *N*-9 acyl compound (0.10 g) was dissolved in a 1:1 mixture of DCM (2 mL) and TFA (2 mL) and stirred at room temperature for 18 h. After this time, the solvent was removed *in vacuo* and the trifluoroacetate salt of the purine was suspended in EtOAc (5 mL). The suspension was washed with saturated NaHCO_3_ solution (3 × 10 mL) and the organic phase was dried (Na_2_SO_4_). Solvents were removed under reduced pressure and the resulting solid was purified as required.

#### *N*-(3-(6-(Cyclohexylmethoxy)-9*H*-purin-2-ylamino)phenyl)acetamide (27)

The title compound was obtained using acetyl chloride (0.13 mL, 1.77 mmol) with *N-*1-(6-(cyclohexylmethoxy)-9*H*-purin-2-yl)benzene-1,3-diamine (**22**). The crude mixture was purified using chromatography (silica; EtOAc) to obtain the compound as an off-white solid (0.15 g, 60%): mp 83-85°C; IR (cm^−1^) 3294, 2923, 2852, 1971, 1734 υ(amide C=O), 1671 υ(amide C=O), 1586, 1541; ^1^H NMR (300 MHz, DMSO-*d_6_*) δ 1.16 (5H, m, cyclohexyl), 1.74 (6H, m, cyclohexyl), 2.04 (3H, s, CH_3_), 2.88 (3H, s, CH_3_), 4.34 (2H, d, *J =* 6.0 Hz, OCH_2_), 7.02-7.11 (1H, m, H-4′), 7.13-7.23 (1H, dd, *J =* 7.5, 8.0 Hz, H-5′), 7.38-7.46 (1H, m, H-6′), 8.14-8.15 (1H, m, H-2′), 8.43-8.43 (1H, s, H-8), 9.65 (1H, s, NH), 9.86 (1H, br s, CONH); ^13^C NMR (125 MHz, DMSO-*d_6_*) δ 24.0, 25.2, 25.9, 26.0, 29.1, 36.7, 36.8, 71.0, 71.4, 110.2, 110.5, 112.4, 113.0, 113.9, 114.3, 128.3, 137.7, 139.3, 156.0, 160.7, 168.0 (C=O), 168.2 (C=O); LCMS (ES^+^) *m/z* 423.3 [M+H]^+^; λ_max_ (EtOH) 306.5, 209.0 nm. After acidolysis according to the general procedure, the title compound was isolated as an orange solid was achieved without further purification (90 mg, 100%): mp 93-95°C; IR (cm^−1^) 3260, 2923, 2851, 2031, 1666 υ(amide C=O), 1591, 1537; ^1^H NMR (300 MHz, DMSO-*d_6_*) δ 1.04-1.32 (5H, m, cyclohexyl), 1.64-1.84 (6H, m, cyclohexyl), 2.03 (3H, s, CH_3_), 4.34 (2H, d, *J* = 6.0 Hz, OCH_2_), 7.07-7.18 (2H, m, H-4′ and H-6′), 7.47 (1H, dd, *J* = 8.0, 8.5 Hz, H-5′), 7.96 (1H, m, H-2′), 7.99 (1H, s, H-8), 9.30 (1H, s, NH), 9.85 (1H, s, CONH); ^13^C NMR (125 MHz, DMSO-*d_6_*) δ 18.5, 24.0, 25.2, 26.0, 29.2, 36.8, 56.0, 71.0, 110.2, 112.4, 113.9, 114.7, 138.8, 139.3, 141.3, 154.2, 155.5, 160.1, 168.0 (C=O); LCMS (ES^+^) *m/z* 381.4 [M+H]^+^; HRMS (ES^+^) calcd for C_20_H_24_N_6_O_2_ [M+H]^+^ 381.2034, found 381.2037; λ_max_ (EtOH) 293.5, 270.0, 230.0 nm.

#### *N*-(4-(6-(Cyclohexylmethoxy)-9*H*-purin-2-ylamino)phenyl)pivalamide (28)

The title compound was obtained from pivaloyl chloride (0.22 mL, 1.77 mmol) with *N*-1-(6-(cyclohexylmethoxy)-9*H*-purin-2-yl)benzene-1,4-diamine (**21**, 0.20 g, 0.59 mmol), DMAP (70 mg, 0.59 mmol) and triethylamine (0.33 mL, 2.36 mmol) at reflux for 18 h. The crude mixture was purified using column chromatography (silica; EtOAc-petrol; 1:1) to isolate the compound as an off-white solid (92 mg, 31%): mp 109-111°C; IR (cm^−1^) 3421, 3328, 3123, 2969, 2921, 2844, 1732 υ(amide C=O), 1658 υ(amide C=O), 1592, 1561, 1517; ^1^H NMR (300 MHz, DMSO-*d_6_*) δ 1.11 (9H, s, ^t^Bu), 1.16 (5H, m, cyclohexyl), 1.50 (9H, s, ^t^Bu), 1.71 (6H, m, cyclohexyl), 4.34 (2H, d, *J =* 6.0 Hz, OCH_2_), 7.55 (1H, d, *J =* 9.0 Hz, H-2′ and H-6′), 7.67 (1H, d, *J =* 9.0 Hz, H-3′ and H-5′), 8.43 (1H, s, H-8), 9.11 (1H, s, CONH), 9.51 (1H, s, NH); ^13^C NMR (125 MHz, DMSO-*d_6_*) δ 25.9, 26.0, 27.0, 27.1, 27.3, 28.6, 29.2, 36.6, 37.7, 70.9, 71.4, 118.3, 120.4, 120.7, 127.5, 128.7, 132.7, 136.7, 137.7, 155.5, 176.0 (C=O), 176.4 (C=O); LCMS (ES^+^) *m/z* 507.5 [M+H]^+^; λ_max_ (EtOH) 312.0; 266.5, 207.5 nm. After acidolysis according to the general procedure, the title compound was isolated as a pale pink solid and was used without further purification (83 mg, 100%): mp 164-166°C; IR (cm^−1^) 2921, 2850, 2161, 1622 υ(amide C=O), 1589, 1508; ^1^H NMR (300 MHz, DMSO-*d_6_*) δ 0.92-1.48 (14H, m, ^t^Bu and cyclohexyl), 1.70-1.84 (6H, m, cyclohexyl), 4.30.4.34 (2H, d, *J =* 6.0 Hz, OCH_2_), 7.50 (2H, d, *J =* 7.0 Hz, H-2′ and H-6′), 7.60 (2H, d, *J =* 7.0 Hz, H-3′ and H-5′), 7.95 (1H, s, H-8), 9.15 (1H, s, NH), 9.22 (1H, s, CONH); LCMS (ES^+^) *m/z* 423.5 [M+H]^+^; HRMS (ES^+^) calcd for C_23_H_30_N_6_O [M+H]^+^ 423.2503, found 423.2509; λ_max_ (EtOH) 369.5, 300.0, 291.5, 284.0, 207.0 nm.

#### *N*-(4-(6-(Cyclohexylmethoxy)-9*H*-purin-2-ylamino)phenyl)benzamide (29)

The title compound was obtained from benzoyl chloride (0.21 mL, 1.77 mmol) with *N*-1-(6-(cyclohexylmethoxy)-9*H*-purin-2-yl)benzene-1,4-diamine (**21**, 0.20 g, 0.59 mmol), DMAP (70 mg, 0.59 mmol) and triethylamine (0.33 mL, 2.36 mmol). The crude mixture was purified using chromatography (silica; EtOAc-petrol; 1:4) to obtain the compound as a white solid (0.26 g, 81%): mp 206-208°C; IR (cm^−1^) 3301, 3139, 2920, 2845, 1701 υ(amide C=O), 1634 υ(amide C=O), 1549, 1506; ^1^H NMR (300 MHz, DMSO-*d_6_*) δ 1.00-1.39 (5H, s, cyclohexyl), 1.60-1.95 (6H, s, cyclohexyl), 4.35 (2H, d, *J =* 6.0 Hz, OCH_2_), 7.19-7.29 (2H, d, *J =* 8.0 Hz, H-2′ and H-6′), 7.38-7.46 (2H, d, *J =* 8.0 Hz, H-3′ and H-5′), 7.49-7.69 (5H, m, Ph), 7.80-7.88 (1H, t, *J =* 7.5, Hz, Ph), 7.89-7.99 (4H, m, Ph), 8.43 (1H, s, H-8), 9.52 (1H, s, NH); ^13^C NMR (125 MHz, DMSO-*d_6_*) δ 25.2, 26.0, 29.2, 36.8, 71.3, 115.4, 118.0, 120.5, 127.5, 128.3, 128.6, 130.3, 131.3, 132.0, 132.7, 132.9, 135.2, 136.2, 139.2, 152.7, 155.6, 160.6, 165.1 (C=O), 166.5 (C=O); LCMS (ES^+^) *m/z* 546.3 [M+H]^+^; λ_max_ (EtOH) 315.0, 229.5 nm. After acidolysis according to the general procedure, the title compound was isolated as a beige solid was isolated and was used without further purification (81 mg, 100%): mp 260-262°C; IR (cm^−1^) 3300, 3139, 2920, 2845, 1634 υ(amide C=O), 1553, 1506; ^1^H NMR (300 MHz, DMSO-*d_6_*) δ 0.99-1.37 (5H, m, cyclohexyl), 1.58-1.96 (6H, m, cyclohexyl), 4.27-4.40 (2H, d, *J =* 6.0 Hz, OCH_2_), 7.48-7.60 (3H, m, phenyl), 7.62-7.72 (2H, d, *J* = 9.0 Hz, H-2′ and H-6′), 7.73-7.83 (2H, d, *J* = 9.0 Hz, H-3′ and H-5′), 7.91-8.05 (3H, m, phenyl and H-8), 9.28 (1H, s, NH), 10.14 (1H, s, CONH), 12.68-12.93 (1H, br s, N9-H); ^13^C NMR (125 MHz, DMSO-*d_6_*) δ 25.2, 26.0, 29.2, 36.8, 71.0, 118.5, 120.8, 127.5, 128.3, 131.3, 132.5, 135.1, 137.1, 155.5, 165.0 (C=O); LCMS (ES^+^) *m/z* 443.5 [M+H]^+^; HRMS (ES^+^) calcd for C_25_H_26_N_6_O_2_ [M+H]^+^ 443.2190, found 443.2196; λ_max_ (EtOH) 315.0 nm.

#### *N*-[4-(6-Cyclohexylmethoxy-9*H*-purin-2-ylamino)phenyl]isonicotinamide (30)

The title compound was obtained from isonicotinoyl chloride (0.32 g, 1.77 mmol) with *N*-1-(6-(cyclohexylmethoxy)-9*H*-purin-2-yl)benzene-1,4-diamine (**21**, 0.2 g, 1.77 mmol), DMAP (70 mg, 0.59 mmol) and triethylamine (0.33 mL, 2.36 mmol). The crude mixture was washed with saturated NaHCO_3_ solution (20 mL) and beige solid collected by filtration and washed with MeOH (20 mL) (0.14 g, 43%): mp 248-250°C (dec.); IR (cm^−1^) 3339, 2927, 2851, 1664 υ(amide C=O), 1622, 1587, 1544, 1516; ^1^H NMR (300 MHz, DMSO-*d_6_*) δ 1.30-1.07 (5H, m, cyclohexyl), 1.83-1.71 (6 H, m, cyclohexyl), 4.32 (2H, d, *J =* 6.0 Hz, OCH_2_), 7.83 (2H, d, *J =* 8.5 Hz, H-2′ and H-6′), 7.87 (2H, d, *J =* 5.5 Hz, pyridyl), 7.64 (2H, d, *J =* 8.5 Hz, H-3′ and H-5′), 8.78 (2H, d, *J =* 5.5 Hz, pyridyl), 9.05 (1H, s, NH), 10.46-10.33 (1H, s, CONH); ^13^C NMR (125 MHz, DMSO-*d_6_*) δ 25.2, 26.0, 29.3, 36.9, 70.8, 118.1, 120.9, 121.5, 131.4, 138.1, 142.1, 150.2, 154.7, 157.4 (C=O), 159.3 (C=O), 163.3; LCMS (ES^+^) *m/z* 444.4 [M+H]^+^; HRMS (ES^+^) calcd for C_24_H_25_N_7_O 444.2142 [M+H]^+^, found 444.2143; λ_max_(EtOH) 315.5, 271.0, 206.0 nm.

#### Synthesis of urea-substituted 2-arylaminopurines (*e.g*. 31 – see ESI for further example)

To a stirred suspension of the required *N*-(6-cyclo hexylmethoxy-9*H*-purin-2-yl)benzene-diamine (**21** or **22**) (0.20 g, 0.59 mmol) and NaOCN (80 mg, 1.18 mmol) in a mixture of DCM (10 mL) and DMF (3 mL) was added TFA (9 μL, 1.18 mmol) over 2 min. The mixture was stirred under N_2_ for 18 h. Solvents were removed under reduced pressure and the residue was dissolved in DCM (25 ml) and extracted with 0.1 M HCl (3 × 20 mL). The organic extract was dried using (Na_2_SO_4_) and the filtrate was concentrated to give a beige solid as crude mixture of mono- and di-urea compounds. The mixture was dissolved in a 1:1 mixture of DCM (2 mL) and TFA (2 mL) and stirred at room temperature for 18 h. After this time, the solvent was removed and the trifluoroacetate salt of the purine was suspended in EtOAc (5 mL). The suspension was washed with saturated NaHCO_3_ solution (3 × 10 mL) and the organic phase was dried (Na_2_SO_4_). Solvents were removed under reduced pressure and the resulting solid was used without further purification.

#### 1-(3-(6-(Cyclohexylmethoxy)-9*H*-purin-2-ylamino)phenyl) urea (31)

Using *N*-(6-cyclohexylmethoxy-9*H*-purin-2-yl)benzene-1,3-diamine (**22**) the product was obtained as a off-white solid (0.14 g, 64%) was isolated without further purification: mp193-194°C; IR (cm^−1^) 3285 υ(NH_2_), 3159, 2919, 2849, 1674 υ(NN'C=O), 1620, 1589, 1541; ^1^H NMR (300 MHz, DMSO-*d_6_*) δ 1.17 (5H, m, cyclohexyl), 1.74 (6H, m, cyclohexyl), 4.33 (2H, d, *J =* 6.0 Hz, OCH_2_), 7.05 (2H, m, H-4′ and H-6′), 7.43 (1H, dd, *J =* 7.5, 8.0 Hz, H-5′), 7.67 (1H, s, H-2′), 7.95 (1H, s, H-8), 8.46 (1H, s, NH-4′), 9.21 (1H, s, NH), 12.77 (1H, s, NH-9); ^13^C NMR (75 MHz, DMSO-*d_6_*) δ 14.4, 25.6, 26.4, 29.6, 37.3, 71.4, 109.7, 128.5, 138.9, 140.9, 141.7, 156.1, 156.3; LCMS (ES^+^) *m/z* 382.3 [M+H]^+^; HRMS (ES^+^) calcd for C_19_H_23_N_7_O_2_ [M+H]^+^ 382.4311, found 382.4312; λ_max_ (EtOH) 295.0, 272.0, 229.0 nm.

#### Synthesis of amide derivatives (compounds 32-47)

To the appropriate carboxylic acid (**23-24**) (1 mol. equiv.) and DIPEA (2 mol. equiv.) in DMF (3 mL/mmol) was added carbonyldiimidazole (2 mol. equiv.) and the resulting mixture was stirred for 1.5 h at room temperature. The appropriate amine (4 mol. equiv.) was added and the reaction was stirred overnight. Solvents were removed and the residue was extracted into EtOAc or THF depending on solubility. The extract was washed with saturated aqueous NaHCO_3_ and dried (Na_2_SO_4_). The solvent was removed to afford the crude product, which was purified as indicated by either by chromatography on silica, or by using the Biotage SP4 purification system.

#### [3-(6-Cyclohexylmethoxy-9*H*-purin-2-ylamino)phenyl]piperidin-1-ylmethanone (32)

The title compound was prepared using **23** (50 mg, 0.14 mmol), carbonyldiimidazole (45 mg, 0.28 mmol), DIPEA (50 μL, 0.28 mmol), and piperidine (55 μL, 0.56 mmol) in DMF (2 mL). The crude product was purified by chromatography on silica using EtOAc-petrol (9:1) as eluent to give an off-white powder (33 mg, 54%): *R_f_* = 0.48 (EtOAc); mp 133-135°C; IR (cm^−1^) 2923, 2850, 1581, 1539, 1437, 1390, 1348, 1276, 1209, 1114, 975; ^1^H NMR (300 MHz, DMSO-*d*_6_) δ 1.1-1.9 (17H, m, cyclohexyl and piperidyl), 3.34 (br, overlap with H_2_O, NCH_2_), 3.58 (2H, s, NCH_2_), 4.34 (2H, d, *J* = 6.3 Hz, OCH_2_), 6.87 (1H, d, *J* = 7.5 Hz, Ar*H*), 7.31 (1H, dd, *J* = 7.8, 7.9 Hz, Ar*H*), 7.79 (1H, d, *J* = 8.3 Hz, Ar*H*), 7.94 (1H, s, Ar*H*), 8.03 (1H, s, H-8), 9.46 (1H, s, ArNHAr), 12.87 (1H, s br, N9-H); LCMS (ES^+^) *m/z* 435.42 [M+H]^+^; Anal. calcd for C_24_H_30_N_6_O_2_: C, 66.34; H, 6.96; N, 19.34%; found: C, 66.41; H, 7.07; N, 19.04; λ_max_ (EtOH) 273, 293 nm.

#### 3-(6-Cyclohexylmethoxy-9*H*-purin-2-ylamino)-*N*-(4-dimethylaminobutyl)benzamide (33)

The title compound was prepared using **23** (80 mg, 0.22 mmol), carbonyldiimidazole (71 mg, 0.44 mmol), DIPEA (79 μL, 0.44 mmol), and *N,N*-dimethyl-1,4-butanediamine (128 mg, 1.1 mmol) in DMF (3 mL). The crude product was purified using a Biotage SP4 purification system (12 + M KP-NH Si cartridge; MeOH-EtOAc; 1:4) to give a colourless powder (53 mg, 51%): *R_f_* = 0.67 (NH_2_-modified silica – MeOH-EtOAc; 1:4); mp 125-127°C; IR (cm^−1^) 3076, 2920, 2849, 2363, 2337, 1597, 1537, 1483, 1438, 1391, 1352, 1283, 1117, 973; ^1^H NMR (300 MHz, DMSO-*d*_6_) δ 1.00-1.90 (15H, m, cyclohexyl and CH_2_C*H*_2_C*H*_2_CH_2_), 2.11 (6H, s, 2 × CH_3_), 2.21 (2H, t, *J* = 6.9 Hz, Me_2_NC*H*_2_), 3.25 (2H overlap with H_2_O, m, CH_2_C*H*_2_NHCO), 4.35 (2H, d, *J* = 6.1 Hz, OCH_2_), 7.29-7.34 (2H, m, 2 × Ar*H*), 7.85 (1H, m, Ar*H*), 8.01 (1H, s, H-8), 8.31 (1H, s, Ar*H*), 8.38 (1H, t, *J* = 5.3 Hz, CONH), 9.40 (1H, s, ArNHAr); LCMS (ES^+^) *m/z* 466.50 [M+H]^+^; HRMS (ES^+^) calcd for C_25_H_36_N_7_O_2_ [M+H]^+^ 466.2925, found 466.2920; λ_max_ (EtOH) 273, 293 nm.

#### 2-[3-(6-Cyclohexylmethoxy-9*H*-purin-2-anilino)]-*N*-isobutylacetamide (34)

The title compound was prepared using **24** (60 mg, 0.16 mmol), carbonyldiimidazole (50 mg, 0.31 mmol), DIPEA (56 μL, 0.31 mmol), and isobutylamine (64 μL, 0.63 mmol) in DMF (2 mL). The crude product was purified by chromatography (silica; EtOAc-petrol; 9:1) to give a white powder (35 mg, 52%): *R_f_* = 0.59 (EtOAc); mp 144-145°C; IR (cm^−1^) 3280, 3091, 2925, 2853, 1643, 1586, 1437, 1344, 1256, 1161, 974; ^1^H NMR (300 MHz, DMSO-*d*_6_) δ 0.82 (6H, d, *J* = 6.7 Hz, CH(C*H*_3_)_2_), 1.0–2.0 (11H, m, cyclohexyl), 1.69 (1H, m, CH_2_C*H*(CH_3_)_2_), 2.88 (2H, t, *J* = 6.5 Hz, C*H*_2_NHCO), 3.37 (2H, s, ArCH_2_), 4.34 (2H, d, *J* = 6.3 Hz, OCH_2_), 6.82 (1H, d, *J* = 7.6 Hz, Ar*H*), 7.17 (1H, dd, *J* = 7.8, 7.9 Hz, Ar*H*), 7.64 (1H, s, Ar*H*), 7.71 (1H, d, *J* = 8.2 Hz, Ar*H*), 7.97 (1H, t, *J* = 5.6 Hz, CONH), 8.15 (1H, s, H-8), 9.26 (1H, s, ArNHAr), 12.76 (1H, br, N9-H); LCMS (ES^+^) *m/z* 437 [M+H]^+^; HRMS (ES^+^) calcd for C_24_H_33_N_6_O_2_ [M+H]^+^ 437.2660, found 437.2659; λ_max_ (EtOH) 214, 272, 291 nm.

#### 2-[3-(6-Cyclohexylmethoxy-9*H*-purin-2-ylanilino]-*N*-cyclopentylacetamide (35)

The title compound was prepared using **24** (60 mg, 0.16 mmol), carbonyldiimidazole (50 mg, 0.31 mmol), DIPEA (56 μL, 0.31 mmol), and cyclopentylamine (62 μL, 0.63 mmol) in DMF (2 mL). The crude product was purified by chromatography (silica; EtOAc-petrol; 9:1) to give a white powder (14 mg, 20%): *R_f_* = 0.57 (EtOAc); mp 228-229°C; IR (cm^−1^) 3277, 2925, 2851, 1641, 1587, 1537, 1441, 1394, 1251; ^1^H NMR (300 MHz, DMSO-*d*_6_) δ 1.0–1.9 (19H, m, cyclohexyl and cyclopentyl), 3.17 (2H, s, ArC*H*_2_), 3.97 (1H, m, cyclopentyl), 4.34 (2H, d, *J* = 6.7 Hz, OCH_2_), 6.80 (1H, d, *J* = 7.3 Hz, Ar*H*), 7.18 (1H, dd, *J* = 7.4, 7.6 Hz, Ar*H*), 7.60 (1H, s, Ar*H*), 7.71 (1H, d, *J* = 7.7 Hz, Ar*H*), 8.01 (1H, d, *J* = 7.6 Hz, CONH), 9.27 (1H, s, ArNHAr), 12.79 (1H, br, N9-H); LCMS (ES^+^) *m/z* 449 [M+H]^+^; HRMS (ES^+^) calcd for C_25_H_33_N_6_O_2_ [M+H]^+^ 449.2660, found 449.2664; λ_max_ (EtOH) 216, 272 nm.

#### 2-[3-(6-Cyclohexylmethoxy-9*H*-purin-2-ylanilino]-*N*-cyclohexylacetamide (36)

The title compound was prepared using **24** (60 mg, 0.16 mmol), carbonyldiimidazole (50 mg, 0.31 mmol), DIPEA (56 μL, 0.31 mmol), and cyclohexylamine (72 μL, 0.63 mmol) in DMF (2 mL). The crude product was purified by chromatography (silica; EtOAc-petrol; 9:1) to give a white powder (17 mg, 24%): *R_f_* = 0.64 (EtOAc); mp 226-227°C; IR (cm^−1^) 3272, 2922, 2850, 2159, 1639, 1585, 1537, 1442, 1346, 1253, 1126; ^1^H NMR (300 MHz, DMSO-*d*_6_) δ 1.0–1.9 (21H, m, cyclohexyl), 3.35 (2H, s, ArCH_2_ overlap with H_2_O), 3.50 (1H, m, cyclohexyl 3′ CH), 4.34 (2H, d, *J* = 6.2 Hz, OCH_2_), 6.81 (1H, d, *J* = 7.4 Hz, Ar*H*), 7.17 (1H, dd, *J* = 7.6, 8.1 Hz, Ar*H*), 7.59 (1H, s, Ar*H*), 7.71 (1H, d, *J* = 8.5 Ar*H*), 7.89 (1H, d, *J* = 7.7 Hz, CONH), 7.96 (1H, s, H-8), 9.26 (1H, s, ArNHAr), 12.76 (1H, br, N9-H); LCMS (ES^+^) *m/z* 463 [M+H]^+^; HRMS (ES^+^) calcd for C_26_H_35_N_6_O_2_ [M+H]^+^ 463.2816, found 463.2815; λ_max_ (EtOH) 215, 272 nm.

#### [3-(6-Cyclohexylmethoxy-9*H*-purin-2-ylanilino]-*N*-methyl-*N*-cyclohexylacetamide (37)

The title compound was prepared using **24** (50 mg, 0.13 mmol), carbonyldiimidazole (42 mg, 0.26 mmol), DIPEA (43 μL, 0.26 mmol), and *N*-methylcyclohexylamine (69 μL, 0.52 mmol) in DMF (2 mL). The crude product was purified by chromatography (silica; EtOAc-petrol; 9:1) to give a white powder (14 mg, 23%): *R_f_* = 0.67 (EtOAc); mp 134-135°C; IR (cm^−1^) 2922, 2850, 2157, 1583, 1539, 1489, 1437, 1390, 1205, 1123; ^1^H NMR (300 MHz, DMSO-*d*_6_) δ 1.0–1.9 (21H, m, cyclohexyl), 2.72 (3H, s, NCH_3_), 3.65 (2H, s, ArCH_2_), 4.27 (1H, m, cyclohexyl 3′ C*H*), 4.33 (2H, d, *J* = 5.9 Hz, OCH_2_), 6.76 (1H, d, *J* = 7.8 Hz, Ar*H*), 7.18 (1H, dd, *J* = 7.8, 8.0 Hz, Ar*H*), 7.62 (1H, s, Ar*H*), 7.67-7.72 (2H, m, Ar*H* and H-8), 9.27 (1H, s, ArNHAr), 12.79 (1H, br, N9-H); LCMS (ES^+^) *m/z* 477 [M+H]^+^; HRMS (ES^+^) calcd for C_18_H_22_N_5_O_2_ [M+H]^+^ 477.2973, found 477.2972; λ_max_ (EtOH) 216, 272, 291 nm.

#### [3-(6-Cyclohexylmethoxy-9*H*-purin-2-ylanilino]-*N, N*-diisopropylacetamide (38)

The title compound was prepared using **24** (60 mg, 0.16 mmol), carbonyldiimidazole (50 mg, 0.31 mmol), DIPEA (56 μL, 0.31 mmol), and diisopropylamine (89 μL, 0.63 mmol) in DMF (2 mL). The crude product was purified by chromatography (silica; EtOAc-petrol; 9:1) to give a white powder (35 mg, 49%): *R_f_* = 0.71 (EtOAc); mp 126-127°C; IR (cm^−1^) 2925, 2851, 2160, 1586, 1441, 1342, 1211, 1118; ^1^H NMR (300 MHz, DMSO-*d*_6_) δ 1.0–1.9 (11H, m, cyclohexyl), 1.05 (6H, d, *J* = 6.5 Hz, CH(C*H*_3_)_2_), 1.40 (6H, d, *J* = 6.7 Hz, CH(C*H*_3_)_2_), 3.41 (1H, m, 3′ *i*-propyl CH), 3.66 (2H, s, ArCH_2_), 4.00 (1H, m, 3′ *i*-propyl CH), 4.30 (2H, d, *J* = 6.2 Hz, OCH_2_), 6.82 (1H, d, *J* = 7.8 Hz, Ar*H*), 7.18 (1H, dd, *J* = 7.9, 8.3 Hz, Ar*H*), 7.47 (1H, m, Ar*H*), 7.59 (1H, s, Ar*H*), 7.64 (1H, s, H-8), 11.65 (1H, br, N9-H); LCMS (ES^+^) *m/z* 465 [M+H]^+^; HRMS (ES^+^) calcd for C_26_H_37_N_6_O_2_ [M+H]^+^ 465.2973, found 465.2976; λ_max_ (EtOH) 214, 272, 292 nm.

#### [3-(6-Cyclohexylmethoxy-9*H*-purin-2-ylanilino]-*N*-propylacetamide (39)

The title compound was prepared using **24** (50 mg, 0.13 mmol), carbonyldiimidazole (42 mg, 0.26 mmol), DIPEA (43 μL, 0.26 mmol), and *n*-propylamine (42 μL, 0.52 mmol) in DMF (2 mL). The crude product was purified by chromatography (silica; MeOH-EtOAc; 0.5:9.5) to give a white powder (13 mg, 24%): *R_f_* = 0.23 (MeOH-EtOAc; 0.5:9.5); mp 141-142°C (dec.); IR (cm^−1^) 3275, 3081, 2929, 2853, 1648, 1450, 1358, 1256; ^1^H NMR (300 MHz, DMSO-*d*_6_) δ 0.81 (3H, t*, J* = 7.8 Hz, CH_2_C*H*_3_), 1.0–1.81 (13H, m, cyclohexyl and CH_3_C*H*_2_), 2.99 (2H, m, CH_2_C*H*_2_NH), 3.49 (2H, s, ArCH_2_), 4.32 (2H, d, *J* = 6.2 Hz, OCH_2_), 6.79 (1H, d, *J* = 7.3 Hz, Ar*H*), 7.16 (1H, dd, *J* = 7.6, 7.8 Hz, Ar*H*), 7.62 (1H, s, Ar*H*), 7.97 (1H, d, *J* = 8.26 Hz, Ar*H*), 7.97 (2H, br, CONH and H-8), 9.24 (1H, s, ArNHAr), 12.81 (1H, s br, N9-H); LCMS (ES^+^) *m/z* 423 [M+H]^+^; λ_max_ (EtOH) 272 nm.

#### 2-[3-(6-Cyclohexylmethoxy-9*H*-purin-2-ylamino]-*N*-(3-hydroxy-propyl)phenylacetamide (40)

The title compound was prepared using **24** (60 mg, 0.16 mmol), carbonyldiimidazole (50 mg, 0.31 mmol), DIPEA (56 μL, 0.31 mmol), and 3-amino-1-propanol (48 μL, 0.63 mmol) in DMF (2 mL). The crude product was purified by chromatography (silica; MeOH-EtOAc; 0.5:9.5) to give a white powder (24 mg, 34%): *R_f_* = 0.08 (EtOAc); mp 132-133°C; IR (cm^−1^) 3267, 2920, 2845, 1641, 1587, 1494, 1436, 1256; ^1^H NMR (300 MHz, DMSO-*d*_6_) δ 1.0–1.9 (11H, m, cyclohexyl), 1.55 (2H, m, CH_2_C*H*_2_CH_2_), 3.11 (2H, m, C*H*_2_NH), 3.36-3.42 (4H, m, ArC*H*_2_, CH_2_OH), 4.34 (2H, d, *J* = 6.5 Hz, OCH_2_), 6.81 (1H, d, *J* = 7.3 Hz, Ar*H*), 7.18 (1H, dd, *J* = 7.7, 8.3 Hz, Ar*H*), 7.63 (1H, s, Ar*H*), 7.71 (1H, d, *J* = 8.9 Hz, Ar*H*), 8.01 (2H, br m, H-8 and CONH), 9.27 (1H, s, ArNHAr), 12.89 (1H, br, N9-H); LCMS (ES^+^) *m/z* 439 [M+H]^+^; HRMS (ES^+^) calcd for C_23_H_31_N_6_O_3_ [M+H]^+^ 439.2452, found 439.2451; λ_max_ (EtOH) 272, 291 nm.

#### 2-[3-(6-Cyclohexylmethoxy-9*H*-purin-2-ylamino)phenyl]-*N*-(3-imidazol-1-yl-propyl)acetamide (41)

The title compound was prepared using **24** (50 mg, 0.13 mmol), carbonyldiimidazole (42 mg, 0.26 mmol), DIPEA (43 μL, 0.26 mmol), and *N*-(3-aminopropyl)imidazole (62 μL, 0.52 mmol) in DMF (2 mL). The crude product was purified using a Biotage SP4 purification system (12 + M KP-NH silica cartridge; MeOH-EtOAc; 1.5:8.5) to give a white powder (15 mg, 24%): *R_f_* = 0.03 (MeOH-EtOAc; 1:4); mp 192-193°C; IR (cm^−1^) 3272, 3199, 2923, 2843, 1616, 1548, 1434, 1381, 1349, 1220, 1122; ^1^H NMR (300 MHz, DMSO-*d*_6_) δ 1.0–1.9 (13H, m, cyclohexyl and CH_2_C*H*_2_CH_2_), 3.02 (2H, m, C*H*_2_NH), 3.40 (2H, s, ArC*H*_2_CONH), 3.92 (2H, t, *J* = 6.9, CH_2_), 4.32 (2H, d, *J* = 6.5 Hz, OC*H*_2_), 6.81 (1H, d, *J* = 7.6 Hz, Ar*H*), 6.85 (1H, s, imidazole-*H*), 7.13 (1H, s, imidazole-*H*), 7.17 (1H, dd, *J* = 7.8, 8.0 Hz, Ar*H*), 7.57 (1H, s, imidazole-*H*), 7.63 (1H, s. Ar*H*), 7.71 (1H, d, *J* = 7.8 Hz, Ar*H*), 8.1 (1H, s, H-8), 9.27 (1H, s, ArNHAr); LCMS (ES^+^) *m/z* 489 [M+H]^+^; HRMS (ES^+^) calcd for C_26_H_33_N_8_O_2_ [M+H]^+^ 489.2721, found 489.2724; λ_max_ (EtOH) 215, 273, 292 nm.

#### 2-[3-(6-Cyclohexylmethoxy-9*H*-purin-2-ylamino)phenyl]-*N*-(3-dimethylamino-propyl)acetamide (42)

The title compound was prepared using **24** (50 mg, 0.13 mmol), carbonyldiimidazole (42 mg, 0.26 mmol), DIPEA (43 μL, 0.26 mmol), and *N,N*-dimethyl-1,3-propanediamine (67 μL, 0.52 mmol) in DMF (2 mL). The crude product was purified using a Biotage SP4 purification system (12 + M KP-NH silica cartridge; MeOH-EtOAc; 1.5:8.5) to give a white powder (15 mg, 24%): *R_f_* = 0.03 (MeOH-EtOAc; 1:4); mp 96-97°C; IR (cm^−1^) 2924, 2850, 2157, 1587, 1537, 1442, 1352, 1249, 1119; ^1^H NMR (300 MHz, DMSO-*d*_6_) δ 1.03 (2H, d, *J* = 6.6 Hz, CH_2_C*H*_2_CH_2_), 1.0–1.9 (11H, m, cyclohexyl), 2.11 (6H, s, N(C*H*_3_)_2_), 2.19 (2H, m, C*H*_2_N(CH_3_)_2_), 3.25-3.40 (overlap with H_2_O, C*H*_2_NH and ArCH_2_), 4.34 (2H, d, *J* = 6.3 Hz, OCH_2_), 6.80 (1H, d, *J* = 7.6 Hz, Ar*H*), 7.16 (1H, dd, *J* = 7.8, 7.9 Hz, Ar*H*), 7.63 (1H, s, Ar*H*), 7.70 (1H, br, CONH), 7.81 (1H, d, *J* = 7.9 Hz, Ar*H*), 7.95 (1H, s, H-8), 9.17 (1H, s, ArNHAr); LCMS (ES^+^) *m/z* 466 [M+H]^+^; HRMS (ES^+^) calcd for C_25_H_36_N_7_O_2_ [M+H]^+^ 466.2925, found 466.2929; λ_max_ (EtOH) 215, 272, 292 nm.

#### 2-[3-(6-Cyclohexylmethoxy-9*H*-purin-2-ylamino)phenyl]-*N*-(2-dimethylaminoethyl)acetamide (43)

The title compound was prepared using **24** (75 mg, 0.20 mmol), carbonyldiimidazole (65 mg, 0.40 mmol), DIPEA (72 μL, 0.40 mmol), and *N,N*-dimethylethylenediamine (71 mg, 0.80 mmol) in DMF (3 mL). The crude product was purified using a Biotage SP4 purification system (12 + M KP-NH silica cartridge; MeOH-EtOAc; 1:9) to give a colourless powder (56 mg, 62%): *R_f_* = 0.10 (NH_2_-modified silica; MeOH-EtOAc; 1:9); mp 161-162°C; IR (cm^−1^) 3249, 2918, 2845, 1587, 1541, 1438, 1396, 1346, 1292, 1240, 1116; ^1^H NMR (300 MHz, DMSO-*d*_6_) δ 1.0–1.84 (11H, m, cyclohexyl), 2.12 (6H, s, N(C*H*_3_)_2_), 2.27 (2H, t, *J* = 6.7 Hz, C*H*_2_N(Me)_2_), 3.13 (2H, m, C*H*_2_NHCO), 3.34 (ArC*H*_2_, overlap with H_2_O), 4.33 (2H, d, *J* = 6.2 Hz, OCH_2_), 6.81 (1H, d, *J* = 7.4 Hz, Ar*H*), 7.17 (1H, t, *J* = 7.8, 7.8 Hz, Ar*H*), 7.64 (1H, s, Ar*H*), 7.69 (1H, d, *J* = 8.0 Hz, Ar*H*), 7.93 (1H, br t, *J* = 5.2 Hz, CONH), 7.98 (1H, s, H-8), 9.27 (1H, s, ArNHAr), 12.80 (1H, s, N9-H); ^13^C NMR (125 MHz, DMSO-*d_6_*) δ 25.2, 26.0, 29.2, 36.9, 42.6, 45.2, 58.2, 71.0, 116.6, 119.3, 121.4, 128.1, 136.5, 138.8, 141.0, 155.5, 170.0; LCMS (ES^+^) *m/z* 452.65 [M+H]^+^; HRMS (ES^+^) calcd for C_24_H_34_N_7_O_2_ [M+H]^+^ 452.2768, found 452.2766; λ_max_ (EtOH) 272 nm.

#### 2-[3-(6-Cyclohexylmethoxy-9*H*-purin-2-ylamino)phenyl]-*N*-(3-methylbutyl)acetamide (44)

The title compound was prepared using **24** (60 mg, 0.16 mmol), carbonyldiimidazole (52 mg, 0.32 mmol), DIPEA (57 μL, 0.32 mmol), and isopentylamine (74 μL, 0.64 mmol) in DMF (3 mL). The crude product was purified by chromatography (silica; MeOH-EtOAc; 0.5:9.5) to give an off-white solid (34 mg, 48%): *R_f_* = 0.45 (MeOH-EtOAc; 1:9); mp 204-205°C; IR (cm^−1^) 3326, 3232, 3137, 3061, 2920, 2851, 2162, 2023, 1643, 1593, 1530, 1487, 1440, 1390, 1354, 1282, 1240, 1170, 1127; ^1^H NMR (300 MHz, DMSO-*d*_6_) δ 0.84 (6H, d, *J* = 6.6 Hz, (C*H*_3_)_2_CH), 1.0–1.85 (11H, m, cyclohexyl), 1.28 (2H, m, CHC*H*_2_CH_2_), 1.56 (1H, m, (CH_3_)_2_C*H*CH_2_), 3.05 (2H, m, CH_2_C*H*_2_NH), 3.35 (ArCH_2_, overlap with H_2_O), 4.33 (2H, d, *J* = 6.2 Hz, OCH_2_), 6.80 (1H, d, *J* = 7.6 Hz, Ar*H*), 7.17 (1H, dd, *J* = 7.8, 7.8 Hz, Ar*H*), 7.63 (1H, s, Ar*H*), 7.68 (1H, d, *J* = 8.1 Hz, Ar*H*), 7.95 (1H, br t, *J* = 5.3 Hz, CONH), 8.00 (1H, s, H-8), 9.26 (1H, s, ArNHAr), 12.83 (1H, s, N9-H); LCMS (ES^+^) *m/z* 451 [M+H]^+^; HRMS (ES^+^) calcd for C_25_H_35_N_6_O_2_ [M+H]^+^ 451.2816, found 451.2820; Anal. calcd for C_25_H_34_N_6_O_2_: C, 66.64; H, 7.61; N, 18.66%; found: C, 66.44; H, 7.68; N, 18.60; λ_max_ (EtOH) 267 nm.

#### 2-[3-(6-Cyclohexylmethoxy-9*H*-purin-2-ylamino)phenyl]-*N*-(4-dimethylaminobutyl)acetamide (45)

The title compound was prepared using **24** (75 mg, 0.20 mmol), carbonyldiimidazole (65 mg, 0.40 mmol), DIPEA (72 μL, 0.40 mmol), and *N,N*-dimethyl-1,4-butanediamine (93 mg, 0.80 mmol) in DMF (3 mL). The crude product was purified using a Biotage SP4 purification system (12 + M KP-NH silica cartridge; MeOH-EtOAc; 1.5:8.5) to give a colourless powder (61 mg, 64%): *R_f_* = 0.12 (NH_2_-modified silica; MeOH-EtOAc: 1:9); mp 125-127°C; IR (cm^−1^) 3490, 3276, 3052, 2927, 2852, 2779, 1606, 1558, 1495, 1448, 1400, 1354, 1309, 1246, 1217; ^1^H NMR (300 MHz, DMSO-*d*_6_) δ 1.0–1.84 (15H, m, cyclohexyl and CH_2_C*H*_2_C*H*_2_CH_2_), 2.14 (2H, t, *J* = 5.7 Hz, C*H*_2_N(Me)_2_), 3.04 (2H, m, C*H*_2_NHCO), 3.34 (ArCH_2_, overlap with H_2_O), 4.33 (2H, d, *J* = 5.8 Hz, OCH_2_), 6.81 (1H, d, *J* = 7.4 Hz, Ar*H*), 7.17 (1H, dd, *J* = 7.8, 7.9 Hz, Ar*H*), 7.64 (1H, s, Ar*H*), 7.69 (1H, d, *J* = 8.0 Hz, Ar*H*), 8.00 (2H, br, CONH and H-8), 9.27 (1H, s, ArNHAr), 12.82 (1H, s, N9-H); ^13^C NMR (125 MHz, DMSO-*d*_6_) δ 24.4, 25.2, 26.0, 27.0, 29.2, 36.8, 38.5, 42.8, 45.1, 58.7, 71.0, 116.5, 119.2, 121.3, 128.1, 136.6, 141.0, 155.4, 169.8; LCMS (ES^+^) *m/z* 480.71 [M+H]^+^; HRMS (ES^+^) calcd for C_26_H_38_N_7_O_2_ [M+H]^+^ 480.3081, found 480.3084; λ_max_ (EtOH) 272 nm.

#### 2-[3-(6-Cyclohexylmethoxy-9*H*-purin-2-ylamino)phenyl]-*N*-[3-(4-methylpiperazin-1- yl)propyl]acetamide (46)

The title compound was prepared using **24** (75 mg, 0.20 mmol), carbonyldiimidazole (65 mg, 0.40 mmol), DIPEA (72 μL, 0.40 mmol), and 1-(3-aminopropyl)-4-methylpiperazine (126 mg, 0.80 mmol) in DMF (3 mL). The crude product was purified using a Biotage SP4 purification system (12 + M KP-NH silica cartridge; MeOH-EtOAc; 1:4) to give a colourless powder (72 mg, 69%): *R_f_* = 0.09 (NH_2_-modified silica; MeOH-EtOAc; 1:9); mp 157-158°C; IR (cm^−1^) 3614, 3273, 3203, 3108, 3039, 2923, 2848, 2802, 1639, 1606, 1585, 1495, 1451, 1394, 1354, 1131, 1216, 1127; ^1^H NMR (300 MHz, DMSO-*d*_6_) δ 1.0–1.85 (13H, m, cyclohexyl and NCH_2_C*H*_2_CH_2_N), 2.11 (3H, s, NC*H*_3_), 2.22 (8H, br t, *J* = 7.2 Hz, 2 × NC*H*_2_C*H*_2_N), 3.05 (2H, m, CH_2_C*H*_2_NHCO), 3.34 (ArCH_2_, overlap with H_2_O), 4.33 (2H, d, *J* = 6.1 Hz, OCH_2_), 6.80 (1H, d, *J* = 7.5 Hz, Ar*H*), 7.17 (1H, dd, *J* = 7.8, 7.8 Hz, Ar*H*), 7.64 (1H, s, Ar*H*), 7.68 (1H, d, *J* = 8.0 Hz, Ar*H*), 7.97 (2H, br, CONH and H-8), 9.28 (1H, s, ArNHAr), 12.79 (1H, s, N9-H); ^13^C NMR (125 MHz, DMSO-*d*_6_) δ 25.2, 26.0, 26.3, 29.2, 36.8, 37.1, 42.8, 45.7, 52.6, 54.7, 55.4, 71.0, 116.6, 119.2, 121.4, 128.1, 136.5, 141.0, 155.4, 169.9; HRMS (ES^+^) calcd for C_28_H_41_N_8_O_2_ [M+H]^+^ 521.3347, found 521.3352; λ_max_ (EtOH) 272 nm.

#### 2-[3-(6-Cyclohexylmethoxy-9*H*-purin-2-ylamino)phenyl]-*N*-(3-phenylpropyl)acetamide (47)

The title compound was prepared using **24** (75 mg, 0.20 mmol), carbonyldiimidazole (65 mg, 0.40 mmol), DIPEA (72 μL, 0.40 mmol), and 3-phenylpropylamine (108 mg, 0.80 mmol) in DMF (3 mL). The crude product was purified by chromatography (silica; EtOAc) to give an off-white solid (53 mg, 53%): *R_f_* = 0.46 (MeOH-EtOAc; 1:9); mp 154-155°C; IR (cm^−1^) 3273, 3207, 3126, 3080, 2921, 2850, 1616, 1585, 1547, 1489, 1444, 1382, 1350, 1283, 1218, 1178, 1119, 976; ^1^H NMR (300 MHz, DMSO-*d*_6_) δ 1.0-1.85 (13H, m, cyclohexyland CH_2_C*H*_2_CH_2_), 2.55 (2H, t, *J* = 7.6 Hz, C*H*_2_Ph), 3.06 (2H, m, C*H*_2_NHCO), 3.36 (2H, s, ArC*H*_2_CONH), 4.32 (2H, d, *J* = 6.2 Hz, OCH_2_), 6.81 (1H, d, *J* = 7.5 Hz, Ar*H*), 7.13-7.29 (6H, m, Ar*H* and Ph*H*), 7.67 (1H, s, Ar*H*), 7.70 (1H, d, *J* = 8.3 Hz, Ar*H*), 7.95 (1H, s, H-8), 8.06 (1H, t br, *J =* 5.5 Hz, CONH), 9.18 (1H, s, ArNHAr); ^13^C NMR (125 MHz, DMSO-*d*_6_) δ 25.2, 26.0, 29.2, 30.9, 32.5, 36.8, 38.2, 68.3, 71.0, 116.6, 121.4, 125.7, 128.1, 128.2, 128.2, 136.6, 141.7, 155.5, 170.0; LCMS (ES^+^) *m/z* 499.54 [M+H]^+^; HRMS (ES^+^) calcd for C_29_H_35_N_6_O_2_ [M+H]^+^ 499.2816, found 499.2820; λ_max_ (EtOH) 272 nm.

#### Synthesis of 2-arylaminopurine derivatives (48-53)

2,2,2-Trifluoroethyl-3-(6-cyclohexylmethoxy-9*H*-purin-2-ylamino)phenylmethanesulfonate (**26**, 1 mol. equiv.),1,8-diazabicyclo[5.4.0]undec-7-ene (3 mol. equiv.), and the appropriate amine (2.5 mol. equiv.) were heated under microwave conditions in anhydrous THF (2 mL) for 15 min at 160°C. After removal of the solvent, the white solid was extracted with EtOAc or THF and washed with saturated aqueous NaHCO_3_ solution and brine. The filtrate was concentrated to give a residue that was purified by medium pressure chromatography or using the Biotage SP4 purification system.

#### [3-(6-Cyclohexylmethoxy-9*H*-purin-2-ylamino)-phenyl]-*N,N*-dimethylmethanesulfonamide (48)

The product from the reaction of 2,2,2-trifluoroethyl-3-(6-cyclohexylmethoxy-9H-purin-2-ylamino) phenylmethanesulfonate (**26**) (46 mg, 0.09 mmol), 1,8-diazabicyclo[5.4.0]undec-7-ene (42 μl, 0.18 mmol), and dimethylamine (12 μL, 0.18 mmol), was purified by chromatography (silica; EtOAc-petrol; 8:2) to give the title compound as an off-white solid (16 mg, 40%): *R_f_* = 0.14 (EtOAc); mp 132-133°C; IR (cm^−1^) 2922, 2849, 2158, 2027, 1968, 1588, 1440, 1309, 1118; ^1^H NMR (300 MHz, DMSO-*d*_6_) δ 1.0–1.8 (11H, m, cyclohexyl), 2.75 (6H, s, N(CH_3_)_2_), 4.34 (2H, s, SO_2_CH_2_), 4.36 (2H, d, *J* = 6.2 Hz, OCH_2_), 6.96 (1H, d, *J* = 7.6 Hz, Ar*H*), 7.27 (1H, dd, *J* = 7.8, 7.9 Hz, Ar*H*), 7.82 (1H, d, *J* = 7.6 Hz, Ar*H*), 7.86 (1H, s, Ar*H*), 8.02 (1H, s, H8), 9.40 (1H, s, ArNHAr), 12.78 (1H, br, N9-H); ^13^C NMR (75 MHz, DMSO-*d*_6_) δ 25.57, 26.37, 29.62, 37.74, 39.36, 49.55, 54.77, 71.52, 118.68, 121.09, 123.60, 128.62, 130.05, 140.26, 141.74, 155.77; LCMS (ES^+^) *m/z* 445 [M+H]^+^; Found: C, 56.79; H, 6.31; N, 18.61. C_21_H_28_N_6_O_3_S requires C, 56.74; H, 6.35; N, 18.91%; λmax (EtOH) 212.5, 272.5, 294 nm.

#### 1-(3-(6-Cyclohexylmethoxy-9*H*-purin-2-ylamino)phenylmethanesulfonyl)piperidine (49)

The product from the reaction of 2,2,2-trifluoroethyl-3-(6-cyclohexylmethoxy-9*H*-purin-2-ylamino) phenylmethanesulfonate (**26**) (75 mg, 0.15 mmol),1,8-diazabicyclo[5.4.0]undec-7-ene (68 μL, 0.45 mmol), and piperidine (37 μL, 0.38 mmol) was purified by chromatography (silica; EtOAc-MeOH; 9.5:0.5) to give the title compound as a white solid (54 mg, 75%): *R_f_* = 0.26 (EtOAc); mp 217-218°C; IR (cm^−1^) 2922, 2853, 2362, 2338, 1589, 1541, 1437, 1396, 1350, 1310, 1242, 1152, 1121, 1067, 1045; ^1^H NMR (300 MHz, DMSO-*d*_6_) δ 1.0–1.8 (11H, m, cyclohexyl), 1.47 (6H, m, piperidyl C*H*), 3.12 (4H, br m, 2 × piperidyl CH_2_), 4.28 (2H, s, ArCH_2_), 4.35 (2H, d, *J* = 6.2 Hz, OCH_2_), 6.94 (1H, d, *J* = 7.5 Hz, Ar*H*), 7.25 (1H, dd, *J =* 7.9, 8.0 Hz, Ar*H*), 7.81 (1H, d, *J* = 8.5 Hz, Ar*H*), 7.88 (1H, s, Ar*H*), 8.02 (1H, s, H-8), 9.42 (1H, s, ArNHAr), 12.76 (1H, br, N9-H); LCMS (ES^+^) *m/z* 485.18 [M+H]^+^; Anal. calcd for C_24_H_32_N_6_O_3_S: C, 59.48; H, 6.66; N, 17.34%; found: C, 59.22; H, 6.56; N, 17.16; λ_max_ (EtOH) 272, 293 nm.

#### 1-(3-(6-Cyclohexylmethoxy-9*H*-purin-2-ylamino)phenylmethanesulfonyl)piperazine (50)

The product from the reaction of 2,2,2-trifluoroethyl-3-(6-cyclohexylmethoxy-9*H*-purin-2-ylamino)phenylmethanesulfonate (**26**) (75 mg, 0.15 mmol), 1,8-diazabicyclo[5.4.0]undec-7-ene (68 μL, 0.45 mmol), and piperazine (45 mg, 0.53 mmol) was purified using Biotage SP4 (12 + M KP-NH silica cartridge; EtOAc-MeOH; 9.5:0.5) to give the title compound as a colourless solid (66 mg, 91%): *R_f_* = 0.73 (NH_2_-modified silica; EtOAc-MeOH; 8:2); mp 127-128°C; IR (cm^−1^) 2922, 2853, 2362, 2338, 1589, 1541, 1437, 1396, 1350, 1310, 1242, 1152, 1121, 1067, 1045; ^1^H NMR (300 MHz, DMSO-*d*_6_) δ 1.0–1.9 (11H, m, cyclohexyl), 3.11 (4H, br, 2 × piperazinyl CH_2_), 3.16 (4H, br, 2 × piperazinyl CH_2_), 4.33 (2H, s, ArCH_2_), 4.35 (2H, d, *J* = 6.7 Hz, OCH_2_), 6.95 (1H, d, *J* = 7.5 Hz, Ar*H*), 7.26 (1H, dd, *J =* 7.8, 7.9 Hz, Ar*H*), 7.62 (1H, d, *J* = 8.0 Hz, Ar*H*), 7.91 (1H, s, Ar*H*), 7.97 (1H, s, H-8), 9.34 (1H, s, ArNHAr), 12.77 (1H, br, N9-H); LCMS (ES^+^) *m/z* 486 [M+H]^+^; HRMS (ES^+^) calcd for C_23_H_32_N_7_O_3_S [M+H]^+^ 484.2136, found 484.2145; λ_max_ (EtOH) 272, 293 nm.

#### 1-[3-(6-Cyclohexylmethoxy-9*H*-purin-2-ylamino)phenyl]-*N*-(3-dimethylaminopropyl)methanesulfonamide (51)

The product from the reaction of 2,2,2-trifluoroethyl-3-(6-cyclohexylmethoxy-9*H*-purin-2-ylamino)phenylmethanesulfonate (**26**) (60 mg, 0.12 mmol),1,8-diazabicyclo[5.4.0]undec-7-ene (55 μL, 0.36 mmol), and *N,N*-dimethylpropane-1,3-diamine (38 μL, 0.30 mmol) was purified using the Biotage SP4 (12 + M KP-NH silica cartridge; EtOAc-MeOH; 8.5:1.5) to give the title compound as a white solid (55 mg, 92%): *R_f_* = 0.13 (NH_2_-modified silica; EtOAc-MeOH; 9:1); mp 167-168°C; IR (cm^−1^) 2924, 2850, 1589, 1541, 1489, 1441, 1390, 1356, 1308, 1243, 1214, 1146, 1121; ^1^H NMR (300 MHz, DMSO-*d*_6_) δ 1.00-1.90 (13H, m, cyclohexyl and NCH_2_C*H*_2_CH_2_NH), 2.07 (6H, s, 2 × CH_3_), 2.19 (2H, t, *J* = 6.9 Hz, Me_2_NC*H*_2_), 2.95 (2H, t, *J* = 6.7 Hz, CH_2_C*H*_2_NH), 4.23 (2H, s, ArCH_2_), 4.35 (2H, d, *J* = 5.8 Hz, OCH_2_), 6.92 (1H, d, *J* = 5.8 Hz, Ar*H*), 7.26 (1H, dd, *J* = 7.8, 7.8 Hz, Ar*H*), 7.83 (1H, d, *J* = 7.7 Hz, Ar*H*), 7.84 (1H, s, Ar*H*), 8.00 (1H, s, H-8), 9.35 (1H, s, ArNHAr); LCMS (ES^+^) *m/z* 502.36 [M+H]^+^; HRMS (ES^+^) calcd for C_24_H_36_N_7_O_3_S [M+H]^+^ 502.2595, found 502.2600; Anal. calcd for C_24_H_35_N_7_O_3_S: C, 57.46; H, 7.03; N, 19.55%; found: C, 57.76; H, 7.15; N, 19.67%; λ_max_ (EtOH) 272, 293 nm.

#### 1-[3-(6-Cyclohexylmethoxy-9*H*-purin-2-ylamino)phenyl]-*N*-(4-methoxybenzyl)methanesulfonamide (52)

The product from the reaction of2,2,2-trifluoroethyl-3-(6-cyclohexylmethoxy-9*H*-purin-2-ylamino)phenylmethanesulfonate (**26**) (150 mg, 0.3 mmol),1,8-diazabicyclo[5.4.0]undec-7-ene (136 μL, 0.9 mmol), and 4-methoxybenzylamine (97 μL, 0.75 mmol) was purified by chromatography (silica; EtOAc-petrol; 9:1) to give the title compound as a white solid (143 mg, 89%): *R_f_* = 0.37 (EtOAc); mp 118-119°C; IR (cm^−1^) 2922, 2850, 2361, 2338, 2026, 1589, 1541, 1506, 1444, 1395, 1354, 1303, 1244, 1121, 1030; ^1^H NMR (300 MHz, DMSO-*d*_6_) δ 1.00-1.90 (11H, m, cyclohexyl), 3.70 (3H, s, OCH_3_), 4.05 (2H, d, *J* = 9.0 Hz, ArC*H*_2_NH), 4.22 (2H, s, ArC*H*_2_SO_2_NH), 4.35 (2H, d, *J* = 6.1 Hz, OCH_2_), 6.86–6.90 (3H, m, 3 × Ar*H*), 7.23 (2H, d, *J* = 7.0 Hz, Ar*H*), 7.26 (1H, dd, *J* = 7.6, 7.9 Hz, Ar*H*), 7.57 (1H, t, *J* = 6.0 Hz, SO_2_NH), 7.81-7.85 (2H, m, 2 × Ar*H*), 7.99 (1H, s, H-8), 9.37 (1H, s, ArNHAr), 12.77 (1H, s br, N9-H); LCMS (ES^+^) *m/z* 537.40 [M+H]^+^; HRMS (ES^−^) calcd for C_27_H_31_N_6_O_4_S [M-H]^−^ 535.2133, found 535.2143; λ_max_ (EtOH) 272 nm.

#### [3-(6-Cyclohexylmethoxy-9*H*-purin-2-ylamino)phenyl]methanesulfonamide (53)

[3-(6-Cyclohexylmethoxy-9*H*-purin-2-ylamino)phenyl]-*N*-(4-methoxybenzyl)methanesulfonamide (**52**) (80 mg, 0.15 mmol) was stirred in neat TFA (2 mL) for 6 h. Upon completion of the reaction, the TFA was removed *in vacuo* and the residual solid was extracted into EtOAc (50 mL). The organic layer was washed with aqueous NaHCO_3_ (50 mL), dried (Na_2_SO_4_) and concentrated to give a white solid. Purification by chromatography (silica; EtOAc) afforded the title compound as a white powder (53 mg, 85%): *R_f_* = 0.50 (EtOAc); mp 134-135°C; IR (cm^−1^) 3344, 2924, 2852, 2361, 2338, 1719, 1599, 1542, 1488, 1449, 1393, 1336, 1256, 1156, 1123, 1046; ^1^H NMR (300 MHz, DMSO-*d*_6_) δ 1.0–1.9 (11H, m, cyclohexyl), 4.20 (2H, s, ArC*H*_2_SO_2_NH_2_), 4.34 (2H, d, *J* = 6.0 Hz, OCH_2_), 6.85 (2H, s br, SO_2_NH_2_), 6.93 (1H, d, *J* = 7.3 Hz, Ar*H*), 7.26 (1H, dd, *J* = 7.8, 7.9 Hz, Ar*H*), 7.75 (1H, s, Ar*H*), 7.85 (1H, d, *J* = 8.3 Hz, Ar*H*), 7.97 (1H, s, H-8), 9.37 (1H, s, ArNH), 12.77 (1H, br, N9-H); LCMS (ES^+^) *m/z* 417 [M+H]^+^; HRMS (ES^−^) calcd for C_19_H_23_N_6_O_3_S [M-H]^−^ 417.1703, found 417.1702; λ_max_ (EtOH) 272, 292 nm.

#### Synthesis of 2-amino-6-alkoxypurines (55 and 56)

Sodium (5 mol. equiv.) was cautiously dissolved in the appropriate alcohol (3.4 mL/mmol), and to the resulting solution was added 2-amino-6-chloropurine (**54**, 1 mol. equiv). The mixture was stirred at reflux for 18 h and cooled to room temperature. The alcohol was removed *in vacuo* and water (100 mL) was added to the residual solid. The resulting solution was neutralised with AcOH. Purification was achieved either by cooling the mixture to ~ 3°C and collecting the product by filtration under vacuum, or by extracting the product into EtOAc (3 × 100 mL). The combined extracts were dried (Na_2_SO_4_) and the solvent was removed to give the 6-alkoxy-2-aminopurine that was used directly in subsequent steps.

#### *O*^6^-Ethylguanine (55)

The title compound was prepared using 2-amino-6-chloropurine (3.0 g, 18 mmol) and sodium (1.2 g, 53 mmol) in EtOH (80 mL) to give a colourless solid (2.9 g, 93%): mp 280-281°C (dec); ^1^H NMR (300 MHz, DMSO-*d_6_*) δ 1.35 (3H, t*, J* = 7.0 Hz, CH_2_C*H*_3_), 4.43 (2H, q, *J* = 7.0 Hz, OCH_2_), 6.20 (2H, s, NH_2_), 7.81 (1H, s, H-8), 12.41 (1H, br, N9-H).

#### (±)*-O*^6^-sec-Butylguanine (56)

The title compound was prepared using 2-amino-6-chloropurine (1.5 g, 8.9 mmol), sodium (0.60 g, 27 mmol), butan-2-ol (25 mL) and THF (15 mL) to give a colourless powder (1.46 g, 80%): mp 84-85°C; ^1^H NMR (300 MHz, DMSO-*d_6_*) δ 0.91 (3H, t*, J* = 7.4 Hz, C*H*_3_CH_2_), 1.30 (3H, d*, J* = 6.3 Hz, CHC*H*_3_), 1.62 (2H, m, C*H*_2_CH_3_), 5.32 (1H, m, OCH), 6.20 (2H, s, 2-NH_2_), 7.79 (1H, s, H-8), 12.29 (1H, br, N9-H).

#### Synthesis of 6-alkoxy-2-fluoropurines (57 and 58)

To a stirred solution of aqueous HBF_4_ (48%, 20 mol. equiv.) at 0°C was added the appropriate 2-amino-6-alkoxypurine (1 mol. equiv.). To the resulting solution was added NaNO_2_ (2 mol. equiv.) in water (1 mL/mmol of NaNO_2_) dropwise, ensuring that the reaction temperature did not exceed 10°C. The reaction mixture was allowed to warm to room temperature and was stirred for 24 h before being neutralised slowly with saturated Na_2_CO_3_ solution. The resulting precipitate was collected *via* filtration and washed with water. The solid was stirred for 1 h in EtOAc (4 × 150 mL) and filtered. The combined filtrates were concentrated under reduced pressure to yield the product, which was used directly.

#### 6-Ethoxy-2-fluoropurine (57)

The title compound was synthesised using *O*^6^-ethylguanine (**55**) (2.5 g, 14 mmol), 48% aqueous HBF_4_ (25 mL, 280 mmol) and NaNO_2_ (1.9 g, 28 mmol) to give a colourless solid (1.26 g, 50%): mp 220-222°C; ^1^H NMR (300 MHz, DMSO-*d_6_*) δ 1.41 (3H, t*, J* = 7.0 Hz, CH_2_C*H*_3_), 4.57 (2H, q, *J* = 7.0 Hz, OCH_2_), 8.39 (1H, s, H-8), 13.40 (1H, br, N9-H).

#### (±)-6-*sec*-Butoxy-2-fluoropurine (58)

The title compound was synthesised using *O*^6^-*sec*-butylguanine (**56**) (1.0 g, 4.8 mmol), 48% aqueous HBF_4_ (10 mL, 45 mmol) and NaNO_2_ (0.67 g, 9.7 mmol) to give a colourless solid (369 mg, 36%): mp 189-190°C (dec); ^1^H NMR (300 MHz, DMSO-*d_6_*) δ 0.92 (3H, t*, J* = 7.4 Hz, C*H*_3_CH_2_), 1.35 (3H, d*, J* = 6.3 Hz, CHC*H_3_*), 1.71 (2H, m, C*H*_2_CH_3_), 5.32 (1H, m, OCH), 8.38 (1H, s, H-8), 13.34 (1H, br, N9-H); ^19^F NMR (470 MHz, DMSO-*d_6_*) δ -51.94 (s, ArF).

#### *N*-(3-Dimethylaminopropyl)-2-[3-(6-ethoxy-9*H*-purin-2-ylamino)phenyl]acetamide (59)

The title compound was synthesised according to Method I using 6-ethoxy-2-fluoropurine (**57**) (100 mg, 0.55 mmol), 2-(3-aminophenyl)-*N*-(3-dimethylaminopropyl)acetamide (see ESI **S49**; 291 mg, 1.2 mmol), TFE (4 mL), and TFA (0.20 mL, 2.8 mmol). The crude product was purified using the Biotage SP4 purification system (25 + M KP-NH Si cartridge; MeOH-EtOAc; 1:9 to 3:7) to give a semi-pure compound that was further purified by semi-prep HPLC (5 → 100% *v/v* acetonitrile: water: NH_4_OH over 25 min; flow-rate 12.75 mL/min, wavelength 280 nm) to give the title compound as a brown gum (43 mg, 20%):*R_f_* = 0.06 (NH_2_–modified silica; MeOH-EtOAcl 1:9); mp 178-179°C; IR (cm^−1^) 3290, 2937, 2762, 1641, 1602, 1571, 1534, 1493, 1433, 1381, 1336, 1317, 1251, 1163, 1122, 1019;^1^H NMR (300 MHz, DMSO-*d_6_*) δ 1.42 (3H, t, *J* = 7.1 Hz, C*H*_3_CH_2_O), 1.52 (2H, qn, *J* = 6.9 Hz, NCH_2_C*H*_2_CH_2_NH), 2.07 (6H, s, 2 × CH_3_), 2.18 (2H, t, *J* = 7.1 Hz, Me_2_NC*H*_2_), 3.06 (2H, m, CH_2_C*H*_2_NHCO), 3.34 (ArCH_2_, overlap with H_2_O), 4.57 (2H, q, *J* = 7.0 Hz, OCH_2_), 6.81 (1H, d, *J* = 7.6 Hz, Ar*H*), 7.18 (1H, dd, *J* = 7.7, 7.8 Hz, Ar*H*), 7.65 (2H, m, 2 × Ar*H*), 8.00 (2H, br, CONH and H-8), 9.28 (1H, s, ArNHAr), 12.81 (1H, br, N9-H); LCMS (ES^+^) *m/z* 398.54 [M+H]^+^;λ_max_ (EtOH) 272 nm.

#### (±)-2-[3-(6-*sec*-Butoxy-9*H*-purin-2-ylamino)phenyl]-*N*-(3-dimethylaminopropyl)acetamide (60)

The title compound was synthesised according to Method I using 6-*sec*-butoxy-2-fluoropurine (**58**) (75 mg, 0.36 mmol), 2-(3-aminophenyl)-*N*-(3-dimethylaminopropyl)acetamide (see ESI **S49**; 190 mg, 0.81 mmol), TFE (3 mL), and TFA (0.13 mL, 1.8 mmol). The crude product was purified using the Biotage SP4 purification system (25 + M KP-NH Si cartridge; EtOAc → MeOH-EtOAc; 1.5:8.5) to give a semi-pure compound that was further purified by semi-prep HPLC (5 → 100% *v/v* acetonitrile: water: NH_4_OH over 25 min; flow-rate 12.75 mL/min, wavelength 280 nm) to give the title compound as a white solid (43 mg, 28%): *R_f_* = 0.08 (NH_2_-modified silica; MeOH-EtOAc; 1:9); mp 97-98°C; IR (cm^−1^) 3283, 2970, 2935, 2777, 2164, 1587, 1538, 1493, 1438, 1373, 1313, 1245, 1215, 1165, 1114; ^1^H NMR (300 MHz, DMSO-*d_6_*) δ 0.95 (3H, t, *J* = 7.4 Hz, C*H*_3_CH_2_CH(CH_3_)O), 1.37 (3H, d, *J* = 6.2 Hz, OCH(C*H*_3_)), 1.51 (2H, qn, *J* = 7.0 Hz, NCH_2_C*H*_2_CH_2_N), 1.65-1.84 (2H, m, CH_3_C*H*_2_CH), 2.05 (6H, s, 2 × NC*H*_3_), 2.16 (2H, t, *J* = 7.1 Hz, Me_2_NC*H*_2_), 3.06 (2H, m, CH_2_C*H*_2_NHCO), 3.34 (ArC*H*_2_, overlap with H_2_O), 5.42 (1H, m, OCH), 6.80 (1H, d, *J* = 7.5 Hz, Ar*H*), 7.18 (1H, dd, *J* = 7.8, 7.8 Hz, Ar*H*), 7.62 (1H, s, Ar*H*), 7.67 (1H, d, *J* = 7.7 Hz, Ar*H*), 7.98 (1H, s, H-8), 9.24 (1H, s, ArNHAr), 12.78 (1H, br, N9-H);LCMS (ES^+^) *m/z* 426.46 [M+H]^+^; HRMS (ES^+^) calcd for C_22_H_32_N_7_O_2_ [M+H]^+^ 426.2612, found 426.2613; λ_max_ (EtOH) 272 nm.

#### [3-(6-Ethoxy-9*H*-purin-2-ylamino)phenyl]acetic acid (61)

6-Ethoxy-2-fluoropurine (**57**) (0.90 g, 5.0 mmol), 3-aminophenylacetic acid (1.87 g, 12.4 mmol) were combined in TFE (7 mL), to which TFA (1.8 mL, 25 mmol) was added and the reaction mixture was heated to reflux for 24 h. After concentration *in vacuo*, THF (20 mL) and NaOH aqueous solution (1 M, 15 mL) were added to the residue and the resulting mixture was stirred overnight. The pH was adjusted to 1 with conc. HCl and the product was extracted with EtOAc (250 mL). The organic phase was separated, washed with 10 % HCl solution and dried (NaSO_4_). Removal of the solvent gave crude product, to which Et_2_O (100 mL) was added. After allowing the mixture to stand for 3 h the resulting precipitate was collected by filtration under vacuum and washed with Et_2_O (30 mL). Recrystallisation from MeOH gave the pure product as a light brown powder (729 mg, 47%): mp 205-206°C; ^1^H NMR (300 MHz, DMSO-*d*_6_) δ 1.42 (3H, t, *J* = 7.0 Hz, C*H*_3_CH_2_O), 3.50 (2H, s, ArCH_2_,), 4.57 (2H, q, *J* = 6.9 Hz, OCH_2_), 6.81 (1H, d, *J* = 7.2 Hz, Ar*H*), 7.20 (1H, dd, *J* = 7.7, 7.8 Hz, Ar*H*), 7.65 (1H, d, *J* = 8.1 Hz, ArH), 7.75 (1H, s, Ar*H*), 7.90 (1H, s, H-8), 9.29 (1H, s, ArNHAr), 12.78 (1H, br, N9-H); LCMS (ES^+^) *m/z* 314.21 [M+H]^+^.

#### 2-[3-(6-Ethoxy-9*H*-purin-2-ylamino)-phenyl]-*N*-(3-imidazol-1-ylpropyl)acetamide (62)

Carboxylic acid **61** (80 mg, 0.26 mmol) was treated with carbonyldiimidazole (84 mg, 0.52 mmol) and DIPEA (93 μL, 0.52 mmol) in DMF (3 mL) and stirred for 1.5 h at room temperature. *N*-(3-aminopropyl)imidazole (130 mg, 0.80 mmol) was added in one portion and stirring was continued overnight. Solvents were removed and the residue was extracted into EtOAc. The extract was washed with saturated aqueous NaHCO_3_ and dried (Na_2_SO_4_). The solvent was removed to afford the crude product, which was purified using a Biotage SP4 purification system (12 + M KP-NH silica cartridge; EtOAc → 15% MeOH/ EtOAc), followed by purification by semi-prep HPLC (5 → 100% *v/v* acetonitrile: water: NH_4_OH over 25 min; flow-rate 12.75 mL/min, wavelength 280 nm) to give an off-white solid (57 mg, 52%): *R_f_* = 0.55 (NH_2_-modified silica; MeOH-EtOAc; 3:7); mp 176-177°C; IR (cm^−1^) 3268, 2953, 2926, 2362, 2337, 1636, 1578, 1539, 1493, 1436, 1379, 1315, 1251, 1165, 1110, 1079; ^1^H NMR (300 MHz, DMSO-*d*_6_) δ 1.42 (3H, t, *J* = 7.0 Hz, C*H*_3_CH_2_O), 1.83 (2H, m, CH_2_C*H*_2_CH_2_NH), 3.01 (2H, m, CH_2_C*H*_2_NHCO), 3.38 (ArCH_2_, overlap with H_2_O), 3.94 (2H, t, *J* = 6.8 Hz, ImC*H*_2_), 4.57 (2H, q, *J* = 7.1 Hz, OCH_2_), 6.83 (1H, d, *J* = 7.6 Hz, Ar*H*), 6.86 (1H, s, imidazole-*H*), 7.13 (1H, s, imidazole-*H*), 7.19 (1H, dd. *J* = 7.7, 7.7 Hz, Ar*H*), 7.58 (1H, s, imidazole-*H*), 7.65-7.71 (2H, m, 2 × Ar*H*), 8.00 (1H, s, H-8), 8.07 (1H, t, *J* = 5.1 Hz, CONH), 9.27 (1H, s, ArNHAr); LCMS (ES^+^) *m/z* 421.34 [M+H]^+^; HRMS (ES^+^) calcd for C_21_H_25_N_8_O_2_ [M+H]^+^ 421.2095, found 421.2099; λ_max_ (EtOH) 272, 292 nm.

#### 6-Chloro-2-fluoro-9*H*-purine (63) [36]

To a stirred solution of HBF_4_ (48% aqueous, 120 mL) at 0°C, was added 2-amino-6-chloropurine (6.0 g, 35.0 mmol). Over 20 min, a solution of NaNO_2_ (4.9 g, 70.0 mmol) in water (200 mL) was added dropwise, ensuring the temperature remained close to 0°C. The pale yellow solution was raised to room temperature and stirred for 18 h. The resulting solution was neutralised to pH 7 in an ice bath at 0°C, by addition of Na_2_CO_3_ (6.00 g) in water (200 mL). Solvents were removed *in vacuo* and the residual solid was redissolved in MeOH (100 mL) and adsorbed onto silica (250 mL). The crude material was purified by chromatography (silica; MeOH-DCM; 1:9) to afford the title compound as a white crystalline solid (4.52 g, 75%): mp 158-159°C (Lit.,^32^ mp 161-162°C); IR (cm^−1^) 2964, 2785, 1735, 1581;^1^H NMR (300 MHz, DMSO-*d_6_*) δ 8.72 (1H, s, H-8), 14.12 (1H, br s, N9-H); LCMS (ES^+^) *m/z* 172.6 [M+H]^+^; λ_max_ (EtOH) 393 nm.

#### 2-Fluoro-9*H*-purine (64) [37, 38]

To a stirred suspension of 6-chloro-2-fluoropurine (**63**) (0.30 g, 1.74 mmol) and palladium hydroxide on carbon (0.30 g) in MeOH (15 mL) was added ammonium formate (0.34 g, 5.35 mmol). The suspension was heated under reflux for 1h before filtering through a pad of Celite, eluting with MeOH (20 mL). Removal of volatiles under reduced pressure yielded the desired compound as a white solid (240 mg, 100%): mp 219°C (dec.) (lit. [37], decomposed at 216°C); ^1^H NMR (300 MHz, DMSO-*d_6_*) δ 8.60 (1H, s, H-8), 9.01 (1H, s, H-6), 13.9 (1H, s, NH-9); LCMS (ES^+^) *m/z* 139.2 [M+H]^+^.

#### 1-(4-((9*H*-purin-2-yl)amino)phenyl)-3-(2-(piperidin-1-yl)ethyl)urea (65)

Synthesis of the title compound was achieved according to Method I using 2-fluoro-9*H*-purine (**64**) (79 mg, 0.57 mmol) and 1-(4-aminophenyl)-3-(2-(piperidin-1-yl)ethyl)urea (see ESI; **S20**) (0.30 g, 1.15 mmol) with TFA (220 μL, 2.88 mmol) in TFE (4 mL). The product was isolated using the Biotage SP4 system (12 + M KP-NH; MeOH-EtOAc; 1:9) as a pale orange solid (95 mg, 45%): mp 96-98°C; IR (cm^−1^) 3296, 3151, 2920, 2850, 1704, 1629 υ(NN'C=O), 1581, 1547, 1512; ^1^H NMR (300 MHz, DMSO-*d_6_*) δ 1.45 (6H, m, CH_2_), 2.33 (6H, m, N(CH_2_)_3_), 3.17 (2H, m, CH_2_), 6.02 (1H, m, NH), 7.28 (2H, d, *J* = 8.5 Hz, H-2′ and H-6′), 7.63 (2H, d, *J =* 8.5 Hz, H-3′ and H-5′), 8.18 (1H, s, H-8), 8.52 (1H, s, H-6), 8.76 (1 H, s, NH-2), 9.28 (1H, s, NH-4´); ^13^C NMR (125 MHz, DMSO-*d_6_*) δ 24.1, 25.5, 36.4, 54.0, 58.2, 118.1, 119.2, 134.3, 134.9, 155.3, 156.8; LCMS (ES^+^) *m/z* 381.4 [M+H]^+^; HRMS (ES^+^) calcd for C_19_H_24_N_8_O [M+H]^+^ 381.2146, found 381.2142; λ_max_ (EtOH) 334.0, 280.0, 244.0 nm.

#### [3-(9*H*-Purin-2-ylamino)phenyl]acetic acid (66)

To a stirred mixture of 2-fluoro-9*H*-purine (**64**) (700 mg, 5.1 mmol), 3-aminophenylacetic acid (1.72 g, 11.4 mmol) in TFE (10 mL) was added TFA (1.9 mL, 25 mmol) and the reaction mixture was heated to reflux for 24 h. The reaction mixture was cooled to room temperature and then concentrated *in vacuo* to give a brown oil. The oil was redissolved in THF (10 mL) and a solution of KOH (2 g) in water (10 mL) was added, and the resulting solution was stirred overnight. THF was removed and the aqueous layer was adjusted to pH 6 with 2 M HCl. The resulting precipitate was collected by filtration under vacuum and washed with water (30 mL), Et_2_O (15 mL) and MeOH (15 mL). The resulting solid was dried to give the title compound as a light brown powder (462 mg, 34%): mp > 250°C; ^1^H NMR (DMSO-*d_6_*) δ 3.51 (2H, s, ArCH_2_), 6.82 (1H, d, *J* = 7.5 Hz, Ar*H*), 7.21 (1H, dd, *J* = 7.8, 7.9 Hz, Ar*H*), 7.64 (1H, s, Ar*H*), 7.79 (1H, d, *J* = 8.2 Hz, Ar*H*), 8.24 (1H, s, H-8), 8.81 (1H, s, H-6), 9.51 (1H, s, ArNH); LCMS (ES^+^) *m/z* 270.20 [M+H]^+^.

#### *N*-(3-Imidazol-1-yl-propyl)-2-[3-(9*H*-purin-2-ylamino)phenyl]acetamide (67)

Carboxylic acid **66** (100 mg, 0.37 mmol) was stirred with carbonyldiimidazole (120 mg, 0.74 mmol) and DIPEA (133 μL, 0.74 mmol) in DMF (3 mL) at room temperature for 1.5 h. Following this, *N*-(3-aminopropyl)imidazole (185 mg, 1.48 mmol) was added in one portion and stirring was continued overnight. Solvents were removed and the residue was extracted into EtOAc. The extract was washed with saturated aqueous NaHCO_3_ and dried (Na_2_SO_4_). The solvent was removed to afford the crude product, which was purified using a Biotage SP4 purification system (12 + M KP-NH silica cartridge; EtOAc → MeOH-EtOAc; 1:4) to give a light brown solid (55 mg, 40%): *R_f_* = 0.14 (NH_2_-modified silica; MeOH-EtOAc; 1:4); mp 121-122°C; IR (cm^−1^) 3252, 2923, 2362, 2337, 1580, 1537, 1394, 1333, 1282, 1214, 1180, 1105, 1080; ^1^H NMR (300 MHz, DMSO-*d*_6_) δ 1.84 (2H, m, CH_2_C*H*_2_CH_2_), 3.02 (2H, m, C*H*_2_NH), 3.38 (2H, s, ArC*H*_2_CONH), 3.94 (2H, t, *J* = 6.8 Hz, C*H*_2_CH_2_CH_2_NHCO), 6.81-6.88 (2H, m, Ar*H* and imidazole-H), 7.14 (1H, s, imidazole-H), 7.20 (1H, dd, *J* = 7.8, 7.9 Hz, Ar*H*), 7.59 (2H, s and d, *J* = 7.4 Hz, imidazole-H and Ar*H*), 7.78 (1H, d, *J* = 7.9 Hz, Ar*H*), 8.10 (1H, t br, *J* = 5.3 Hz, CONH), 8.23 (1H, s, H-8), 8.79 (1H, s, H-6), 9.46 (1H, s, ArNHAr); LCMS (ES^+^) *m/z* 375.39 [M+H]^+^; HRMS (ES^+^) calcd for C_19_H_21_N_8_O [M+H]^+^ 375.1687, found 375.1689; λ_max_ (EtOH) 272, 330 nm.

#### Synthesis of 2-arylamino-6-(dialkylaminovinyl)-purines (69-73)

A solution of 6-ethynyl-2-phenylaminopurine (**68**) (50 mg, 0.21 mmol) and the required secondary amine (20.0 eq.) in anhydrous THF (2 mL) was subjected to microwave heating at 100°C for 10 minutes in a sealed nitrogen flushed Biotage microwave vial. The cooled solution was partitioned between EtOAc (20 mL) and saturated NaHCO_3_ solution (20 mL). The organic extract was concentrated *in vacuo* to a yellow/orange syrup which was isolated using the Biotage SP4 purification system (12 + M KP-NH; MeOH-DCM; 0.5:9.5).

#### (*E*)-*N*-Phenyl-6-(2-(pyrrolidin-1-yl)vinyl)-9*H*-purin-2-amine (69)

The title compound was prepared by reaction of pyrrolidine (355 μL, 4.23 mmol). Yellow solid (60 mg, 93%): mp 138-140°C; IR (cm^−1^) 3040, 2957, 1358, 2921, 2852, 1630, 1559; ^1^H NMR (500 MHz, DMSO-*d_6_*) δ 1.94 (4H, m, CH_2_), 3.36 (4H, m, CH_2_), 5.32-5.35 (1H, d, *J* = 15.0 Hz, alkene CH), 6.85-6.87 (1H, t, *J* = 10.1 Hz, H-4′), 7.22-7.26 (2H, dd, *J* = 9.9, 10.1 Hz, H-3′ and H-5′), 8.82-8.84 (2H, d, *J* = 9.9 Hz, H-2′ and H-6′), 7.91 (1H, s, H-8), 8.54-8.57 (1H, d, *J* = 15.0 Hz, alkene CH), 8.97(1H, br s, NH), 12.52 (1H, br s, NH-9); LCMS (ES^+^) *m/z* 307.3 [M+H]^+^; HRMS (ES^+^) calcd for C_17_H_18_N_6_ [M+H]^+^ 307.1671, found 307.1666; λ_max_ (EtOH) 362.5, 282.5, 254.5 nm.

#### (*E*)-6-(2-(Dimethylamino)vinyl)-*N*-phenyl-9*H*-purin-2-amine (70)

The title compound was prepared by reaction dimethylamine solution 2.0 M in THF (2.13 mL, 4.23 mmol). Yellow solid (63%): mp 129-131°C; IR (cm^−1^) 2946, 2922, 2853, 1562, 1525; ^1^H NMR (500 MHz, DMSO-*d_6_*) δ 2.99 (6H, s, NCH_3_), 5.15-5.18 (1H, d, *J* = 15.0 Hz, alkene CH), 6.63-6.65 (1H, t, *J* = 10.0 Hz, H-4′), 7.00-7.04 (2H, dd, *J* = 9.9, 10.0 Hz, H-3′ and H-5′), 7.60-7.62 (2H, d, *J* = 9.9 Hz, H-2′ and H-6′), 7.70 (1H, s, H-8), 8.08-8.11 (1H, d, *J* = 15.0 Hz, alkene CH), 8.75(1H, br s, NH), 12.30 (1H, br s, NH-9); ^13^C NMR (125 MHz, DMSO-*d_6_*) δ 84.5, 91.0, 117.9, 119.8, 122.0, 128.2, 138.1, 141.9, 149.6, 151.5, 156.0, 156.6; LCMS (ES^+^) *m/z* 281.2 [M+H]^+^; HRMS (ES^+^) calcd for C_15_H_16_N_6_ [M+H]^+^ 281.1508, found 281.1509; λ_max_ (EtOH) 358.0, 281.0, 253.0 nm.

#### (*E*)-6-(2-(Diethylamino)vinyl)-*N*-phenyl-9*H*-purin-2-amine (71)

The title compound was prepared by reaction of diethylamine (442 μL, 4.23 mmol). Yellow solid (39 mg, 60%): mp 127-129°C; IR (cm^−1^) 2970, 2911, 1630, 1559; ^1^H NMR (500 MHz, DMSO-*d_6_*) δ 1.11-1.13 (6H, t, *J* = 5.0 Hz, CH_3_), 3.21-3.24 (4H, q, *J* = 5.0 Hz, NCH_2_), 5.52-5.53 (1H, d, *J* = 15.0 Hz, alkene CH), 6.96-7.00 (1H, dd, *J* = 10.1 Hz, H-4′), 7.19 (1H, s, H-8), 7.21-7.25 (2H, dd, *J* = 9.8, 10.1 Hz, H-3′ and H-5′), 7.40 (1H, br s, NH-2), 7.44-7.46 (2H, d, *J* = 9.8 Hz, H-2′ and H-6′), 8.18-8.21 (1H, d, *J* = 15.0 Hz, alkene CH), 13.05 (1H, br s, NH-9); ^13^C NMR (125 MHz, DMSO-*d_6_*) δ 29.7, 22.2, 121.3, 122.8, 123.0, 129.3, 137.7, 140.16, 148.3, 151.1, 156.0, 158.3; LCMS (ES^+^) *m/z* 309.3 [M+H]^+^; HRMS (ES^+^) calcd for C_17_H_20_N_6_ [M+H]^+^ 309.1824, found 309.1822; λ_max_ (EtOH) 359.0, 279.5, 253.5 nm.

#### (*E*)-6-(2-(Azepan-1-yl)vinyl)-*N*-phenyl-9*H*-purin-2-amine (72)

The title compound was prepared by reaction of homopiperadine (480 μL, 4.23 mmol). Yellow solid (69 mg, 98%): mp 135-137°C; IR (cm^−1^) 3030, 2921, 2850, 1629, 1559; ^1^H NMR (500 MHz, CDCl_3_) δ 1.49 (4H, m, CH_2_), 1.64-1.71 (4H, m, CH_2_), 3.28-3.38 (4H, m, CH_2_), 5.49-5.52 (1H, d, *J* = 15.0 Hz, alkene CH), 6.95-6.99 (1H, t, *J* = 10.5 Hz, H-4′), 7.19 (1H, s, H-8), 7.22-7.26 (2H, dd, *J* = 9.5, 10.5 Hz, H-3′ and H-5′), 7.32 (1H, br s, NH), 7.44-7.46 (2H, d, *J* = 9.5 Hz, H-2′ and H-6′), 8.23-8.26 (1H, d, *J* = 15.0 Hz, alkene CH), 12.89 (1H, br s, NH-9); ^13^C NMR (125 MHz, CDCl_3_) δ 25.9, 26.9, 28.2, 30.5, 48.1, 56.0, 121.2, 122.8, 123.0, 129.3, 137.6, 140.1, 149.8, 151.0, 156.0, 158.3; LCMS (ES^+^) *m/z* 335.3 [M+H]^+^; HRMS (ES^+^) calcd for C_19_H_22_N_6_ [M+H]^+^ 335.1975, found 335.1979;λ_max_ (EtOH) 360.0, 238.0, 254.5 nm.

#### (*R, E*)-6-(2-(3-(Dimethylamino)pyrrolidin-1-yl)vinyl)-*N*-phenyl-9*H*-purin-2-amine (73)

The title compound was prepared by reaction of (*R*)-3-dimethylaminopyrrolidine (540 μL, 4.23 mmol). Yellow solid (68 mg, 93%): mp 136-138°C; IR (cm^−1^) 2954, 2778, 2118, 1625, 1561, 1528; ^1^H NMR (500 MHz, CDCl_3_) δ 1.75-1.83 (1H, m, pyrrolidine H-3′'), 2.08 (1H, m, pyrrolidine CH), 2.20 (6H, s, N(CH_3_)_2_), 2.70 (1H, m, pyrollidine CH), 3.49-3.50 (4H, m, CH_2_), 5.40 (1H, d, *J* = 15.0 Hz, alkene CH), 6.79-7.00 (1H, t, *J* = 8.0 Hz, H-4′), 7.23-7.26 (2H, dd, *J* = 7.5, 8.0 Hz, H-3′ and H-5′), 7.45-7.46 (2H, d, *J* = 7.5 Hz, H-2′ and H-6′), 8.31 (1H, d, *J* = 15.0 Hz, alkene CH); ^13^C NMR (125 MHz, DMSO-*d_6_*) δ 14.2, 29.7, 30.2, 44.3, 65.2, 76.8, 77.1, 77.3, 91.8, 121.2, 122.7, 123.0, 129.3, 137.7, 140.0, 146.2, 151.2, 155.9, 155.8; LCMS (ES^+^) *m/z* 350.3 [M+H]^+^; HRMS (ES^+^) calcd for C_19_H_23_N_7_ [M+H]^+^ 350.2085, found 350.2088; λ_max_ (EtOH) 263.0, 282.0, 361.0 nm.

### Kinase inhibition conter-screening assays

Assays were conducted using ProfilerPro kinase selectivity assay kit 1 (Caliper Life Sciences). The ATP concentration used was that of the apparent ATP *K_M_* of each individual kinase and inhibitors were incubated at a single concentration of 2 μM, giving percentage inhibition values. Briefly, the protocol (provided with the kit on purchase) involved thawing of frozen enzyme and peptide/ATP plates before reconstitution of the enzymes in buffer solution. Compound solutions were added to the enzyme plate, mixed and pre-incubated at 28°C for 15 min before transferring the peptide/ATP solution to each well. The reaction mixture was incubated at 28°C for a further 90 min before addition of stop-buffer solution. The complete assay reactions were read using the Caliper EZ Reader II. For more information see: reference 16 and http://www.perkinelmer.co.uk/product/ez-reader-ship-level-122919 (accessed *via* internet on 05/07/2016).

### Cellular growth inhibition assays

CellTiter-Blue Assay for Growth Inhibition. U2OS human osteosarcoma cells (American Type Culture Collection, Manassas, Virginia, United States) were grown in McCoy's 5A medium supplemented with 1.5 mM L-glutamine, 25 mM HEPES, 2% penicillin/streptomycin (Invitrogen, Paisley, United Kingdom) and 10% (*v/v*) foetal calf serum (FCS) (Biosera, Ringmer, East Sussex, United Kingdom). MDA-MB-231 human breast cancer cells (American Type Culture Collection, Manassas, Virginia, United States) were grown in RPMI 1640 medium (Invitrogen) supplemented with 2 mM L-glutamine, 25 mM HEPES, 2% penicillin/streptomycin and 10% (*v/v*) FCS. HeLa cells were grown in Dulbecco's Modified Eagle Medium (D-MEM) (Invitrogen) supplemented with 2% penicillin/streptomycin and 10% (*v/v*) FCS. All three cell lines were maintained in a humidified atmosphere of 5% CO_2_ at 37°C. The medium was aspirated and the cells were washed with PBS (Invitrogen), trypsinized (Internal supply, 0.25% versene trypsin with EDTA), neutralized and counted. Cells were seeded into 384-well clear tissue culture treated microtiter plates (Corning B.V. Life Sciences, Amsterdam, The Netherlands) at 200 cells per well in a 45 μL volume of the respective media. Columns 1 and 24 had no cells added and were plated with 45 μL of media alone. Cells were incubated at 37°C / 5% CO_2_. At 24 hours after plating, compounds were three-fold serially diluted in large volume V-shape 384-well microplates (Greiner Bio-One, Stonehouse, Gloucestershire, United Kingdom) using an Evolution plate handling system (PerkinElmer Life Sciences, Waltham, Massachusetts, USA). Then 5 μL of diluted test compounds, etoposide as positive control (Sigma-Aldrich, Gillingham, Dorset, United Kingdom), or DMSO at 1% *v/v* final concentration (Fisher Scientific, Loughborough, Leicestershire, United Kingdom) were added to the wells using a MiniTrack V plate handling system (PerkinElmer Life Sciences). There were four replicates of each compound concentration, 32 replicates of DMSO wells, and 32 replicates of wells containing no cells. Test compounds were screened at final concentrations of 100 μM, 33.33 μM, 11.11 μM, 3.70 μM, 1.23 μM, 0.41 μM, 0.14 μM, and 0.05 μM. Etoposide was screened at final concentrations of 10 μM, 3.33 μM, 1.11 μM, 0.37 μM, 0.12 μM, 0.041 μM, 0.014 μM, and 0.005 μM. After 92 hours, 5 μL of CellTiter-Blue Reagent (Promega, Southampton, United Kingdom) was added to the cells using a Multidrop dispenser (Thermo Electron, Basingstoke, Hants, United Kingdom) and incubated for 4 hours in a humidified atmosphere of 5% CO_2_ at 37°C. After the incubation, the plates were placed at room temperature for 40 minutes before fluorescence was recorded (Ex = 560/ Em = 590) on an EnVision 2103 plate reader (PerkinElmer Life Sciences). Data were plotted as percentage of DMSO control against compound concentration using GraphPad Prism 5 Software. The 50% growth inhibition (GI_50_) was calculated as the compound concentration required to reduce the cell number by 50% compared with the DMSO control.

### Nek2 inhibition reversibility assay

The reversible nature of Nek2 inhibition by enamine **70** was measured using a kinase inhibition-reversibility assay. The assay mixture initially contained the ‘peptide 11′ substrate (5-FAM-KKLNRTLSVA-COOH; Caliper Life Sciences) and Nek2 protein (Invitrogen) at a concentration 100-fold that of the standard inhibitory activity assay. This solution in reaction buffer was pre-incubated with inhibitor **70** at a concentration ten-fold its IC50, calculated to afford approximately 91% inhibition of Nek2. After incubation of the enzyme with compound **70** for 30 min, the system was diluted 100- fold into a buffer solution containing both peptide-11 and ATP at the concentrations of the standard assay (details given in reference 12), affording a very low concentration of inhibitor (0.1 × IC50) with respect to other substrates. Following rapid 1:100 dilution, a reaction progress curve was generated by monitoring for levels of the phosphorylated substrate as described in reference 16 using the Caliper EZ Reader II instrument at different times throughout the duration of the reaction.

### HPLC analysis of enamine stability

Solutions of 10 μM **72** were prepared in100% MEM media, 100% RPMI media, 10% (v/v) FCS in MEM, 10% BSA in water (*v/v*), 10 % FCS (v/v) in water, or 10 % (v/v) boiled FCS in water, DMSO and phosphate buffered water at pH 2, pH 7 and pH 10 by adding 10 μL of the 1 mM working stock of **72** in MeCN to 990 μL of each medium. A 50 μL aliquot was removed directly and immediately extracted as described below. At 5, 10, 15, 30, 60, 90, 120, 180, 240, 360 and 1440 minutes after addition of the drug further aliquots of 50 μL were taken and extracted as below. At each time point the 50 μL of the test solution was added to 50 μL of acetonitrile (MeCN). Samples were mixed for 10 seconds on a vortex mixer and centrifuged at 15700 rcf for 5 minutes in a microcentrifuge. The supernatant solution from each sample was placed into an HPLC vial for analysis. Samples were analysed by reverse phase HPLC with PDA detection.

## SUPPLEMENTARY MATERIALS FIGURES AND TABLES


