# Supplementary material for: Structure-guided design of purine-based probes for selective Nek2 inhibition
Source: Oncotarget. 2016 Nov 9;8(12):19089–124. doi: 10.18632/oncotarget.13249 (PMC5386672; doi:10.18632/oncotarget.13249)
Supplement: Supplementary file 1 [file oncotarget-08-19089-s001.pdf]

Structure-guided design of purine-based probes for selective Nek2 inhibition

SUPPLEMENTARY MATERIALS

DETAILS OF SYNTHETIC PROCEDURES AND CHARACTERISATION OF ANILINE PRECURSORS AND FURTHER EXAMPLES OF PURINE-BASED COMPOUNDS OMITTED FROM THE MAIN ARTICLE

Precursors to carboxamides 10 and 11

_N_-Methyl-3-nitrobenzamide (S1)

To a stirred solution of 3-nitrobenzoyl chloride (2.0 g, 11 mmol) in DCM (15 mL) was added methylamine (33% in EtOH, 6.7 mL, 54 mmol) slowly over 15 min. After stirring at room temperature for 2 h the solvent was removed. The white residue was extracted into EtOAc (2 × 100 mL). The combined extracts were washed with aq. sodium bicarbonate (1 × 100 mL) and water (1 × 100 mL). The organic layer was dried (Na_2SO_4) and concentrated to give the title compound as a white solid (1.93 g, 99%): R_f =0.52 (petrol-EtOAc; 6:4); mp 173-174 °C (Lit., 356 mp 175 °C); ^{1}H NMR (300 MHz, DMSO-d_6) δ 2.83 (3H, d, *J* = 4.6 Hz, $CONHCH_3$), 7.78 (1H, dd, *J* = 8.0, 8.0 Hz, ArH), 8.28 (1H, d, *J* = 7.9 Hz, ArH), 8.38 (1H, ddd, *J* = 1.0, 2.3, 8.2 Hz, ArH), 8.60 (1H, dd, *J* = 1.9, 2.0 Hz, ArH), 8.84 (1H, br, CONH).

3-Nitro-_N,N_-dimethylbenzamide (S2)

To a stirred solution of 3-nitrobenzoyl chloride (0.50 g, 2.7 mmol) in THF (4 mL) was added dimethylamine (2.0 M in THF, 6.7 mL, 14 mmol) slowly over 15 min. After stirring at room temperature for 18 h, the solvent was removed. The brown residue was extracted into DCM (50 mL). The extract was washed with aq. $NaHCO_3$ (50 mL) and water (50 mL). The organic layer was dried (Na_2SO_4) and concentrated to give the title compound as a white solid (0.52 g, 99%): mp 80-82 °C (Lit., 357 mp 83-84 °C); ^{1}H NMR (300 MHz, DMSO-d_6) δ 2.92 (3H, s, CH_3), 3.02 (3H, s, CH_3), 7.74 (1H, dd, *J* = 7.8, 8.0 Hz, ArH), 7.87 (1H, ddd, *J* = 1.2, 1.4, 7.7 Hz, ArH), 8.21 (1H, dd, *J* = 1.8, 1.9 Hz, ArH), 8.29 (1H, ddd, *J* = 1.1, 1.1, 8.2 Hz, ArH).

Reduction of aryl-nitro derivatives to corresponding anilines (compounds S3 and S4)

To a stirred solution of compound **S1** or **S2** in anhydrous solvent as indicated was added 10% palladium on activated carbon. The resulting mixture was stirred under an atmosphere of H_2 at room temperature for 24 h. Alternatively, as indicated, hydrogen was replaced by ammonium formate (5 mol. equiv.), which was added to the suspension. The reaction mixture was filtered through a bed of Celite eluting with MeOH-DCM (1:9) to afford the title compound following removal of the solvent *in vacuo*.

3-Amino-_N_-methylbenzamide (S3)

The title compound was prepared using *N*-methyl-3-nitrobenzamide (**S1**, 1.0 g, 5.6 mmol) and 10% palladium on carbon (150 mg) under H_2 in EtOH (10 mL) and EtOAc (10 mL) to give 3-amino-*N*-methylbenzamide as a colourless powder (0.8 g, 99%): R_f = 0.29 (petrol-EtOAc: 6:4); mp 85-86 °C (Lit., 358 mp 88-90 °C); ^{1}H NMR (300 MHz, DMSO-d_6) δ 2.73 (3H, d, *J* = 4.6 Hz, $CONHCH_3$), 5.21 (2H, s, $ArNH_2$), 6.66 (1H, ddd, *J* = 0.8, 2.3, 7.9 Hz, ArH), 6.91 (1H, d, *J* = 7.7 Hz, ArH), 7.01 (1H, dd, *J* = 1.9, 2.0 Hz, ArH), 7.06 (1H, dd, *J* = 7.8, 7.8 Hz, ArH), 8.18 (1H, br, CONH).

3-Amino-_N,N_-dimethylbenzamide (S4)

The title compound was prepared using *N,N*-dimethyl-3-nitrobenzamide (**S2**, 0.40 g, 2.1 mmol), ammonium formate (0.65 g, 10 mmol) and 10% palladium

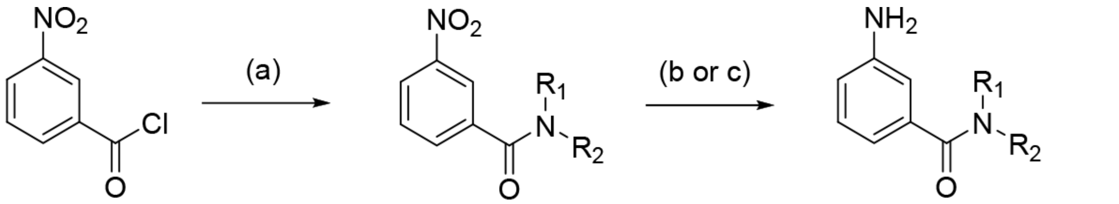

S1; R_1 = H, R_2 = Me, 99%
S2; R_1 = R_2 = Me, 99%

S3; R_1 = H, R_2 = Me, 99%
S4; R_1 = R_2 = Me, 99%

Reagents and conditions: (a) R_1R_2NH, THF; (b) Pd/C, HCO_2NH_4, MeOH; (c) Pd/C, H_2, EtOH/EtOAc.

on carbon (56 mg) in MeOH (5 mL). The crude mixture was extracted into EtOAc (50 mL), washed with H_2O (50 mL), dried (Na_2SO_4) and concentrated *in vacuo* to give a brown crystalline solid (322 mg, 95%): mp 82-83 °C (Lit., 357 mp 86-87 °C); ^{1}H NMR (300 MHz, DMSO-d_6) δ 2.91 (6H, s, $N(CH_3)_2$), 5.23 (2H, br s, $ArNH_2$), 6.47 (1H, ddd, *J* = 1.2, 1.2, 7.5 Hz, ArH), 6.55 (1H, dd, *J* = 1.6, 1.9 Hz, ArH), 6.60 (1H, ddd, *J* = 0.9, 2.3, 8.0 Hz, ArH), 7.04 (1H, dd, *J* = 7.7, 7.8 Hz, ArH).

Precursors to thiocarboxamides 12 and 13

Synthesis of 3-nitrobenzothioamides (compounds S5 and S6)

To a solution of amide **S2** or 3-nitrobenzamide (1 mol. equiv.) in anhydrous THF (3 mL/mmol), was added Lawesson's reagent (1.1 mol. equiv.). The mixture was heated at reflux overnight and allowed to cool to room temperature. The solvent was removed *in vacuo* and the residue was extracted into EtOAc (100 mL). The extract was washed with 1 M NaOH aqueous solution and dried ($MgSO_4$). The solvents were removed to furnish the crude product, which was purified by chromatography on silica.

N,N-Dimethyl-3-nitrothiobenzamide (S5)

The title compound was prepared using *N,N*-dimethyl-3-nitrobenzamide (0.50 g, 2.6 mmol) with Lawesson's reagent (1.15 g, 2.8 mmol) and was purified by chromatography on silica (petrol-EtOAc; 6:4) to give a yellow powder, *N,N*-dimethyl-3-nitrothiobenzamide (241 mg, 44%): mp 131-132 °C (Lit.,[360] mp 131-132 °C); ^{1}H NMR (300 MHz, $CDCl_3$) δ 3.21 (3H, s, NCH_3), 3.63 (3H, s, NCH_3), 7.49 (1H, dd, *J* = 7.9, 7.9 Hz, Ar*H*), 7.59 (1H, ddd, *J* = 1.4, 1.4, 7.5 Hz, Ar*H*), 8.09-8.15 (2H, m, Ar*H*).

3-Aminothiobenzamide (S6)

The title compound was prepared using 3-nitrobenzamide (5.0 g, 30 mmol) with Lawesson's reagent (13.4 g, 33 mmol), and was purified by chromatography on silica (petrol-EtOAc; 6:4) to give a yellow powder, 3-aminothiobenzamide(3.47 g, 64%): mp 125-126 °C (Lit.,[361] 126-127 °C); ^{1}H NMR (300 MHz, DMSO-d_6) δ 7.72 (1H, dd, *J* = 8.0, 8.0 Hz, Ar*H*), 8.28 (1H, ddd, *J* = 1.0, 1.7, 7.8 Hz, Ar*H*), 8.35 (1H, ddd, *J* = 0.9, 2.3, 8.2 Hz, Ar*H*), 8.70 (1H, dd, *J* = 2.0, 2.0 Hz, Ar*H*), 9.86 (1H, br, NH), 10.23 (1H, br, NH).

Reduction of nitro-aryl thioamides (compounds S7 and S8)

To a stirred solution of the nitroaryl thioamide **S5** or **S6** in glacial acetic acid (25 mL/g) was added zinc powder. The resulting mixture was stirred under nitrogen at room temperature for 18 h. The suspension was filtered through Celite eluting with MeOH. After removal of the MeOH, the residual acetate salt was disolved in water (20 mL) and neutralised by dropwise addition of conc. ammonia solution until pH 8. After cooling for 1 h, the precipitated aniline was isolated by filtration and washed with water (30 mL).

3-Amino-*N,N*-dimethylthiobenzamide (S7)

The title compound was prepared using *N,N*-dimethyl-3-nitrothiobenzamide (**S5**) (200 mg, 0.95 mmol) and zinc powder (311 mg, 4.8 mmol) to give a yellow powder (172 mg, 100%): R_f = 0.58 (EtOAc-petrol; 7:3); ^{1}H NMR (300 MHz, $CDCl_3$) δ 3.17 (3H, s, NCH_3), 3.58 (3H, s, NCH_3), 6.63-6.67 (3H, m, Ar*H*), 7.12 (1H, dd, *J* = 7.7, 8.5 Hz, Ar*H*).

3-Aminothiobenzamide (S8)

The title compound was prepared using 3-nitrothiobenzamide (**S6**) (350 mg, 1.9 mmol) and zinc powder (628 mg, 9.6 mmol) to give a yellow powder (287 mg, 100%): R_f = 0.33 (EtOAc-petrol; 7:3); mp 135-136 °C (Lit.,[361] mp 133-135 °C); ^{1}H NMR (300 MHz, DMSO-d_6) δ 5.25 (2H, s, ArNH_2), 6.66 (1H, ddd, *J* = 1.0, 2.3, 7.9 Hz, Ar*H*), 6.92 (1H, ddd, *J* = 1.2, 1.6, 7.7 Hz, Ar*H*), 7.02 (1H, dd, *J* = 7.7, 7.8 Hz, Ar*H*), 7.11 (1H, dd, *J* = 2.0, 2.0 Hz, Ar*H*), 9.28 (1H, br, SCNH), 9.67 (1H, br, SCNH).

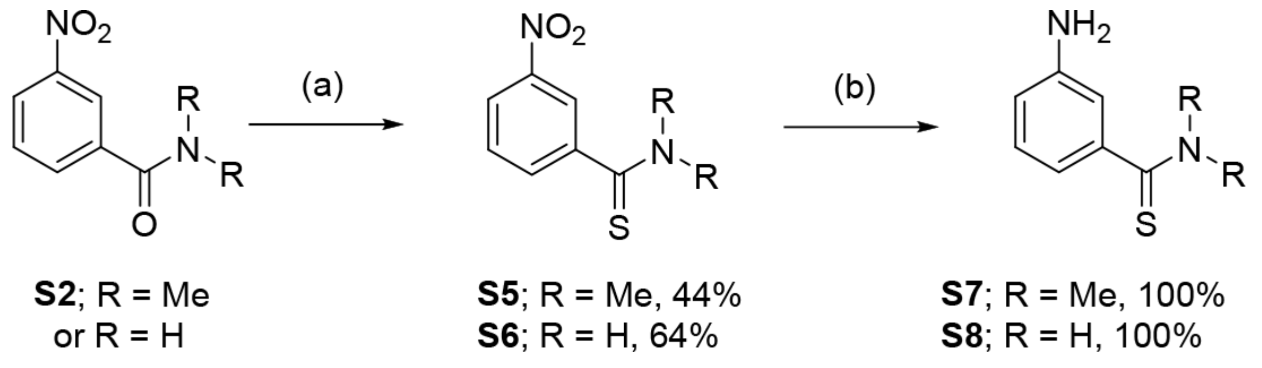

Reagents and conditions: (a) Lawesson's reagent, THF, reflux, 18 h; (b) Zn, AcOH, RT, 18 h; (c) 13, trifluoroacetic acid, 2,2,2-trifluoroethanol, 90 °C, 18 h.

Precursor to sulfonamide 14

N,N-Dimethyl-3-nitrobenzenesulfonamide (S9)

To a stirred solution of dimethylamine hydrochloride (1.1 g, 14 mmol) and DIPEA (3.6 mL, 20 mmol) in DCM (10 mL) was added 3-nitrobenzenesulfonyl chloride (1.0 g, 4.5 mmol) over 15 min, and the reaction mixture was stirred at room temperature for 18 h. The mixture was concentrated to give a viscous oil that was extracted into EtOAc (100 mL) and washed with aqueous sodium bicarbonate solution (100 mL). The aqueous layer was re-extracted with EtOAc (100 mL) and the organic layers were combined, dried (Na_2SO_4) and concentrated to give the title compound as an off-white solid (1.0 g, 97%): mp 119-120 °C (Lit.,[359] mp 120-121 °C); ^{1}H NMR (300 MHz, DMSO-d_6) δ 2.68 (6H, s, N(CH_3)$_2$), 7.96 (1H, dd, *J* = 8.0, 8.1 Hz, Ar*H*), 8.20 (1H, ddd, *J* = 1.1, 1.6, 7.8 Hz, Ar*H*), 8.39 (1H, dd, *J* = 1.9, 1.9 Hz, Ar*H*), 8.56 (1H, ddd, *J* = 1.0, 2.3, 8.2 Hz, Ar*H*).

3-Amino-*N,N*-dimethylbenzenesulfonamide (S10)

The title compound was prepared by treatment of *N,N*-dimethyl-3-nitrobenzenesulfonamide (**S9**, 0.80 g, 3.5 mmol) with 10% palladium on carbon (0.15 g) in a mixture of EtOAc-EtOH (1:1; 10 mL) under H_2. The mixture was stirred at room temperature for 18 h and then filtered through a bed of Celite and concentrated *in vacuo* to give the title compound as a colourless solid (0.67 g, 96%): mp 151-152 °C (Lit.,[359] mp 154-156 °C); ^{1}H NMR (300 MHz, DMSO-d_6) δ 2.58 (6H, s, N(CH_3)$_2$), 5.64 (2H, s, ArNH_2), 6.77-6.84 (2H, m, 2 × Ar*H*), 6.91 (1H, dd, *J* = 1.9, 2.0 Hz, Ar*H*), 7.24 (1H, dd, *J* = 7.8, 7.9 Hz, Ar*H*).

Precursor to homocarboxamide 15

2-(3-Nitrophenyl)-*N*-methylacetamide (S11)

To a solution of 3-nitrophenylacetic acid (0.50 g, 2.8 mmol) in THF (5 mL) was added $SOCl_2$ (0.22 mL, 3.1 mmol) and the reaction mixture was stirred at room temperature for 2 h. Solvents were removed *in vacuo* and the brown residue was re-dissolved in THF (4 mL), which was added dropwise to a solution of methylamine (40% aq., 0.70 mL, 12.6 mmol) over 15 min, and the reaction mixture was stirred for 18 h at room temperature. Solvents were removed, and the resulting brown residue was extracted into DCM (50 mL). The extract was washed with aq. $NaHCO_3$ (50 mL) and water (50 mL). The organic layer was dried (Na_2SO_4) and concentrated to give the title compound as a brown solid (0.35 g, 64%): mp 100-102 °C (Lit.,[363] 101-103 °C); ^{1}H NMR (300 MHz, DMSO-d_6) δ 2.59 (3H, d, *J* = 4.6 Hz, CH_3), 3.58 (2H, s, ArCH_2), 7.61 (1H, dd, *J* = 7.8, 7.9 Hz, Ar*H*), 7.71 (1H, d, *J* = 7.8 Hz, Ar*H*), 8.11 (1H, d, *J* = 8.1 Hz, Ar*H*), 8.15 (1H, dd, *J* = 1.7, 1.8 Hz, Ar*H*).

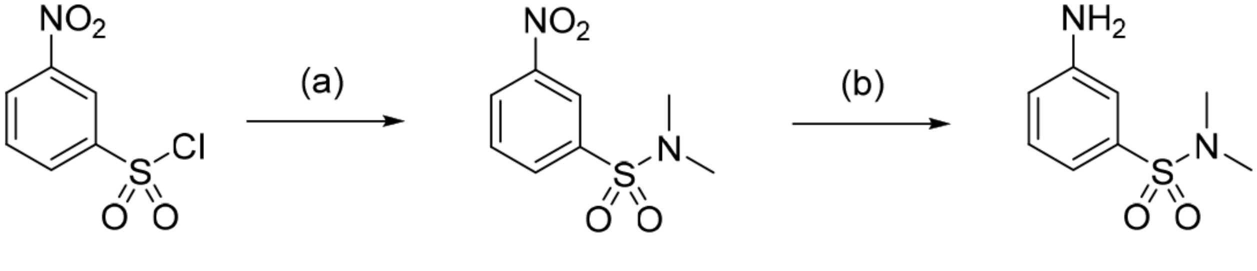

Reagents and conditions: (a) Me2NH, DIPEA, DCM, RT, 18 h, 97%; (b) Pd/C, H_2, EtOH, RT, 18 h 96%.

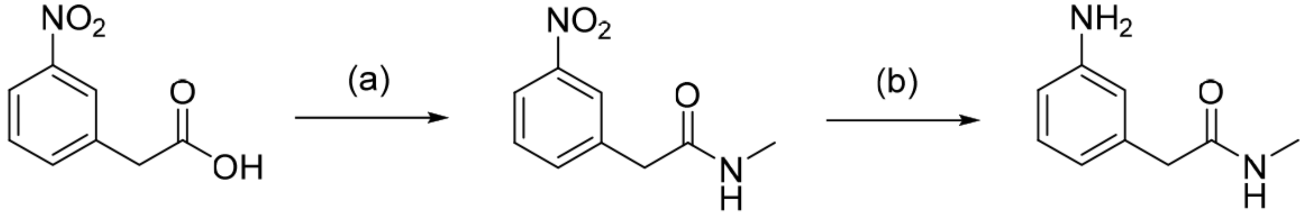

a Reagents and conditions: (a) (i) $SOCl_2$, THF, RT, 2 h (ii) $MeNH_2$, RT, 18 h 64%; (b) Pd/C, HCO_2NH_4, MeOH, RT, 18 h, 98%; (c) 13, trifluoroacetic acid, 2,2,2-trifluoroethanol, 90 °C, 18 h,45%.

2-(3-Aminophenyl)-*N*-methylacetamide (S12)

2-(3-Nitrophenyl)-*N*-methylacetamide (**S11**) (300 mg, 1.6 mmol), ammonium formate (489 mg, 7.8 mmol) and 10% palladium on carbon (50 mg) were added to MeOH (5 mL) and stirred at room temperature for 18 h. The crude mixture was filtered through a pad of Celite, washing with MeOH (50 mL) and concentrated *in vacuo*. Crude material was extracted into EtOAc (50 mL), washed with H_2O (50 mL), dried (Na_2SO_4) and concentrated *in vacuo* to give a brown crystalline solid, 2-(3-aminophenyl)-*N*-methylacetamide(250 mg, 98%): mp 45-46 °C (Lit.,[363] mp 44-46 °C); ^{1}H NMR (300 MHz, DMSO-d_6) δ 2.56 (3H, d, *J* = 4.6 Hz, $NHCH_3$), 3.20 (2H, s, $ArCH_2$), 5.00 (2H, br, $ArNH_2$), 6.34-6.42 (2H, m, Ar*H*), 6.44 (1H, dd, *J* = 1.6, 1.7 Hz, Ar*H*), 6.90 (1H, dd, *J* = 7.7, 7.7 Hz, Ar*H*), 7.88 (1H, br, CONH); ^{13}C NMR (75 MHz, DMSO-d_6) δ 26.0, 43.2, 112.6, 115.1, 117.1, 129.0, 137.2, 148.8, 171.0.

Precursors to secondary ureas 16-18; and further examples

Preparation of nitrophenyl-ureas (S13-S17)

To a stirred solution of the required 3- or 4-nitrophenyl isocyanate (1.00 g, 6.1 mmol) in anhydrous THF (50 mL) was added the required amine (7.3 mmol) and the resulting mixture was stirred at ambient temperature under an atmosphere of nitrogen for 3 h. The mixture was concentrated *in vacuo* and purified as indicated below.

1-(2-Morpholinoethyl)-3-(4-nitrophenyl)urea (S13)

The title compound was prepared using 4-nitrophenyl isocyanate (1.00 g, 6.1 mmol) and 4-(2-aminoethyl)morpholine (0.95 mL, 7.3 mmol) in anhydrous THF (50 mL). The crude mixture was concentrated *in vacuo* and DCM (50 mL) was added. The resulting suspension was filtered under suction and washed with DCM (50 mL) to yield the title compound as a yellow solid (1.43 g, 80%): R_f = 0.63 (MeOH-DCM; 1:9); mp 199-201 ºC; IR (cm^{-1}) 3332, 3210, 3159, 3100, 2949, 2812, 1662 υ(NN'C=O), 1557, 1492 υ (strong, br, NO_2 asymmetric stretch), 1413 υ (strong, sharp, NO_2 symmetric stretch); ^{1}H NMR (300 MHz, DMSO-d_6) δ 2.48-2.51 (6H, m, $N(CH_2)_3$), 3.24 (2H, dt, *J* = 6.0, 12.0 Hz, CH_2), 3.59 (4H, t, *J* = 5.0 Hz, OCH_2), 6.39 (1H, t, *J* = 6.0 Hz, NH), 7.62 (2H, d, *J* = 9.0 Hz, H-2' and H-6'), 8.14 (2H, d, *J* = 9.0 Hz, H-3' and H-5'), 9.46 (1H, s, NH); ^{13}C NMR (75 MHz, DMSO-d_6) δ 37.3, 53.9, 58.4, 67.2, 118.2, 125.6, 142.6, 146, 155; LCMS (ES$^+$) *m/z* 295.1 $[M+H]^+$; λ_{max} (EtOH) 331.0, 221.0 nm.

1-(2-Morpholinoethyl)-3-(3-nitrophenyl)urea (S14)

The title compound was synthesised using 3-nitrophenyl isocyanate (1.00 g, 6.1 mmol) and 4-(2-aminoethyl)morpholine (0.95 mL, 7.3 mmol) in anhydrous THF (50 mL). The crude mixture was concentrated *in vacuo* and subsequently purified by chromatography (silica; MeOH-DCM; 1:9) to afford the title compound as a viscous yellow oil, which crystallised to give a yellow solid after trituration under Et_2O (1.7 g, 95%): R_f = 0.44 (MeOH-DCM; 1:9); mp 125-127 °C; IR (cm^{-1}) 3355, 3186, 3136, 3079, 2955, 2819, 1657 υ(NN'C=O), 1547, 1509 υ (strong, br, NO_2 asymmetric stretch), 1341 υ (strong, sharp, NO_2 symmetric stretch); ^{1}H NMR (300 MHz, $CDCl_3$) δ 2.48 (6H, m, $N(CH_2)_3$), 3.34 (2H, dd, *J* = 5.5, 11.0 Hz, CH_2), 3.61-3.71 (4H, t, *J* = 4.5 Hz, OCH_2 morpholine), 5.61 (1H, br s, NH), 7.34 (1H, dd = 7.5, 8.0 Hz, H-5'), 7.75 (2H, m, H-4' and H-6'), 8.11 (1H, dd, *J* = 2.0, 2.5 Hz, H-2'); ^{13}C NMR (75 MHz, $CDCl_3$) δ 54.0, 67.2, 114.4, 117.7, 125.5, 130.1, 140.9, 149.3, 155.7; LCMS (ES$^+$) *m/z* 295.1 $[M+H]^+$; λ_{max} (EtOH) 339.5, 241.5 nm.

1-(4-Nitrophenyl)-3-(2-(piperidin-1-yl)ethyl)urea (S15)

The title compound was prepared using 4-nitrophenyl isocyanate (1.00 g, 6.1 mmol) and 4-(2-aminoethyl)piperidine (0.96 mL, 7.3 mmol) in anhydrous THF (50 mL). The mixture was concentrated *in vacuo* and DCM (50 mL) was added. The resulting suspension was filtered, and washed with DCM (50 mL). The title compound was isolated as an off-white solid (1.17 g, 67%): R_f = 0.38 (MeOH-DCM; 1:9); mp 177-

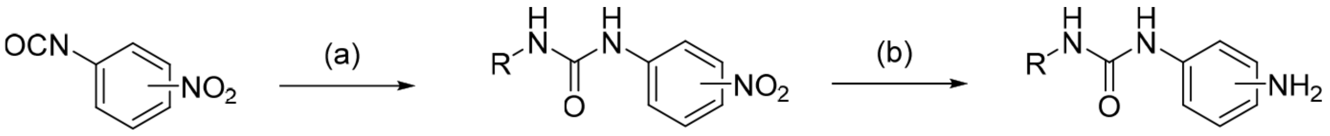

S13-S17 **S18-S22**

Reagents and conditions: (a) amine, THF, RT, 3h; (b) Pd/C, H_2, MeOH, RT, 18 h, 85-94%;

179 ºC; IR (cm^{-1}) 3323, 3275, 3211, 3156, 3090, 2935, 2803, 1667 υ(NN'C=O), 1554, 1491 υ(strong, br, NO_2 asymmetric stretch), 1321 υ(strong, sharp, NO_2 symmetric stretch); ^{1}H NMR (300 MHz, $CDCl_3$) δ 1.47-1.58 (2H, m, CH_2), 1.59-1.70 (4H, m, CH_2), 1.92-1.96 (2H, m, CH_2), 2.56 (4H, m, CH_2), 3.37 (2H, dt, J = 5.0, 10.0 Hz, CH_2), 5.55 (1H, br m, NH), 7.59 (2H, d, J = 9.0 Hz, H-2' and H-6'), 8.19 (2H, d, J = 9.0 Hz, H-3' and H-5'); ^{13}C NMR (75 MHz, $CDCl_3$) δ 24.4, 26.1, 38.7, 55.1, 59.4, 118.6, 125.2, 125.6, 146.7; LCMS (ES$^+$) *m/z* 293.3 [M+H]$^+$; λ_{max} (EtOH) 332.5, 205.0 nm.

1-(3-(Imidazol-1-yl)propyl)-3-(4-nitrophenyl)urea (S16)

The title compound was prepared using 4-nitrophenyl isocyanate (1.00 g, 6.1 mmol) and 1-(3-aminopropyl) imidazole (0.87 mL, 7.3 mmol) in anhydrous THF (50 mL). The mixture was concentrated *in vacuo*, DCM (50 mL) was added and the resulting suspension was filtered. After washing the residue with DCM (50 mL), the title compound was isolated as a pale yellow solid (1.50 g, 85%): mp 195-197 ºC; IR (cm^{-1}) 3303, 3113, 2784, 1664 υ(NN'C=O), 1599, 1570 υ(strong, br, NO_2 asymmetric stretch), 1479 υ(strong, sharp, NO_2 symmetric stretch); ^{1}H NMR (300 MHz, DMSO-d_6) δ 1.82-1.95 (2H, quin., J = 6.5 Hz, CH_2), 3.08 (2H, dt, J = 6.5, 7.0 Hz, CH_2NH), 4.00 (2H, t, J = 7.0 Hz, CH_2), 6.54-6.61 (1H, br t, J = 5.5 Hz, NH), 6.88-6.91 (1H, m, CH imidazole), 7.19-7.22 (1H, d, J = 8.0 Hz, CH imidazole), 7.60-7.66 (2H, d, J = 9.0 Hz, H-2' and H-6'), 7.69-7.70 (1H, d, J = 8.0 Hz, CH imidazole), 8.11-8.17 (2H, d, J = 9.0 Hz, H-3' and H-5'), 9.28-9.38 (1H, br s, NH-4'); ^{13}C NMR (75 MHz, DMSO-d_6) δ 31.5, 37.0, 117.4, 119.6, 125.3, 128.8, 137.6, 148.3, 154.9; LCMS (ES$^+$) *m/z* 290.3 [M+H]$^+$; λ_{max} (EtOH) 329.5, 218.0 nm.

1-(3-Dimethylaminopropyl)-3-(4-nitrophenyl)urea (S17) [19]

The title compound was prepared using 4-nitrophenyl isocyanate (1.00 g, 6.1 mmol) and 3-dimethylamino-1-propylamine (0.91 mL, 7.3 mmol) in anhydrous THF (50 mL). The mixture was concentrated *in vacuo* and purified by chromatography (silica; MeOH-DCM; 1:9) to afford the title compound as a pale yellow solid (1.43 g, 88%): mp 80-82 ºC; IR (cm^{-1}) 3411, 3319, 2939, 1687 υ(NN'C=O), 1558 υ(strong, br, NO_2 asymmetric stretch), 1494 υ(strong, sharp, NO_2 symmetric stretch), 1453; ^{1}H NMR (300 MHz, DMSO-d_6) δ 1.52-1.64 (2H, quin., J = 7.0, Hz, CH_2), 2.16 (6H, s, $N(CH_3)_2$), 2.23-2.32 (2H, t, J = 7.0 Hz, CH_2), 3.13 (2H, dt, J = 5.5, 7.0 Hz, CH_2), 6.55 (1H, br t, J = 5.5 Hz, NH), 7.62 (2H, d, J = 9.0 Hz, H-2' and H-6'), 8.13 (2H, d, J = 9.0 Hz, H-3' and H-5'), 9.36 (1H, s, NH-4'); ^{13}C NMR (75 MHz, DMSO-d_6) δ 28.1, 38.3, 45.0, 57.5, 117.4, 125.9, 141.0, 148.1, 155.2; LCMS (ES$^+$) *m/z* 267.3 [M+H]$^+$; λ_{max} (EtOH) 358.0, 348.5 341.0, 255.5 nm.

Reduction of aryl-nitro derivatives (compounds S18-S22)

To a stirred solution of compound **S13-S17** in MeOH (10 mL) was added 10% palladium on activated carbon. The resulting mixture was stirred under an atmosphere of H_2 at room temperature for 24 h. Alternatively, as indicated, hydrogen was replaced by ammonium formate (5 mol. equiv.), which was added to the suspension. The product was purified by filtration through a bed of Celite eluting with 10% MeOH in DCM, to afford the title compound following removal of the solvent *in vacuo*.

1-(4-Aminophenyl)-3-(2-morpholinoethyl)urea (S18)

The title compound was prepared using 1-(2-morpholinoethyl)-3-(4-nitrophenyl)urea (**S13**) (1.00 g, 3.97 mmol) and 10% palladium on activated carbon (0.30 g) in MeOH (10 mL). The analytically pure title compound was obtained as an off-white solid following removal of solvent *in vacuo* (0.78 g, 87%): R_f = 0.20 (MeOH-DCM; 1:9); mp 179-181 ºC; IR (cm^{-1}) 3332 υ(NH_2), 2870, 1632 υ(NN'C=O), 1594, 1569, 1513; ^{1}H NMR (300 MHz, DMSO-d_6) δ 2.18-2.45 (6H, m, $N(CH_2)_3$), 3.08-3.24 (2H, dt, J = 6.0, 12.0 Hz, CH_2), 3.48-3.79 (4H, m, OCH_2), 4.68 (2H, br s, NH_2), 5.68-5.90 (1H, t, J = 6.0 Hz, NH), 6.48-6.56 (2H, d, J = 8.5 Hz, H-2' and H-6'), 6.96-7.05 (2H, d, J = 8.5 Hz, H-3' and H-5'), 8.07 (1H, s, NH-4'); ^{13}C NMR (75 MHz, $CDCl_3$) δ 37.3, 53.7, 58.4, 67.2, 116.2, 120.6, 142.6, 146, 155.1; LCMS (ES$^+$) *mz* 265.1 [M+H]$^+$; λ_{max} (EtOH) 304.0, 252.5 nm.

1-(3-Aminophenyl)-3-(2-morpholinoethyl)urea (S19) [20]

The title compound was prepared using 1-(2-morpholinoethyl)-3-(3-nitrophenyl)urea (**S14**) (1.90 g, 6.46 mmol) and 10% palladium on activated carbon (0.57 g) in MeOH (10 mL). Following filtration through Celite and concentration of the filtrate *in vacuo*, the title compound was obtained as an off-white solid (1.54 g, 90%): mp 123-125 ºC; ^{1}H NMR (300 MHz, $CDCl_3$) δ 2.41-2.62 (6H, m, $N(CH_2)_3$), 3.30-3.36 (2H, dt, J = 5.5, 6.0 Hz, CH_2), 3.51 (1H, br s, NH), 3.64-3.82 (4H, m, OCH_2), 5.43 (1H, br t, J = 5.5 Hz, NH), 7.20 (1H, m, H-2'), 7.30-7.38 (1H, dd, J = 2.0, 8.5 Hz, H-6'), 7.73-7.79 (1H, dd, J = 8.0, 8.5 Hz, H-5'), 6.81 (1H, dd, J = 2.0, 8.0 Hz, H-4'); ^{13}C NMR (300 MHz, $CDCl_3$) δ 37.4, 53.8, 58.2, 67.2, 108.6, 111.3, 111.8, 130.3; LCMS (ES$^+$) *m/z* 265.2 [M+H]$^+$.

1-(4-Aminophenyl)-3-(2-(piperidin-1-yl)ethyl)urea (S20)

The title compound was prepared using 1-(4-nitrophenyl)-3-(2-(piperidinyl)ethyl)urea (**S15**) (0.50 g, 1.71 mmol) and 10% palladium on activated carbon (0.57 g) in MeOH (20 mL). Following filtration through Celite and concentration of the filtrate *in vacuo*, the compound was isolated as a pale off-white solid (0.46

g, 94%): mp 141-143 °C; IR (cm^{-1}) 3385 υ(NH_2), 3211, 2931, 2844, 2786, 1637 υ(NN'C=O), 1596, 1557, 1507; ^{1}H NMR (300 MHz, DMSO-d_6) δ 1.45 (6H, m, N(CH_2)$_3$), 3.14 (2H, m, CH_2), 3.43 (4H, m, CH_2), 4.65 (2H, m, CH_2), 5.81 (1H, br t, *J* = 7.0 Hz, NH), 6.46 (2H, d, *J* = 7.5 Hz, H-2' and H-6'), 7.00 (2H, d, *J* = 7.5 Hz, H-3' and H-5'), 8.09 (1H, s, NH-4'); ^{13}C NMR (300 MHz, DMSO-d_6) δ 59.6, 24.4, 26.1, 38.8, 55.4, 118.7, 125.5, 146.1; LCMS (ES$^+$) *m/z* 263.3 [M+H]$^+$; λ_{max} (EtOH) 302.5, 252.0 nm.

1-(4-Aminophenyl)-3-(3-imidazol-1-ylpropyl)urea (S21)

The title compound was prepared using 1-(3-imidazol-1-ylpropyl)-3-(4-nitrophenyl)urea (**S16**) (1.00 g, 3.46 mmol) and 10% palladium on activated carbon (0.30 g) in MeOH (20 mL). Following filtration through Celite and concentration of the filtrate *in vacuo*, the title compound was obtained as a pale pink solid following trituration under Et_2O (0.83 g, 93%): mp 140-142 ºC; IR (cm^{-1}) 3302 υ(NH_2), 3198, 3107, 2931, 2875, 1624 υ(NN'C=O), 1566, 1508; ^{1}H NMR (300 MHz, DMSO-d_6) δ 1.80-1.85 (2H, quin., *J* = 7.0 Hz, CH_2), 2.99-3.01 (2H, dt, *J* = 5.5, 7.0 Hz, CH_2), 3.97 (2H, t, *J* = 7.0 Hz, CH_2), 4.69 (2H, br s, NH_2), 6.06 (1H, t, *J* = 5.5 Hz, NH), 6.46 (2H, d, *J* = 8.5 Hz, H-2' and H-6'), 6.89 (1H, m, CH imidazole), 7.01 (2H, d, *J* = 8.5 Hz, H-3' and H-5'), 7.19 (1H, m, CH imidazole), 7.64 (1H, m, CH imidazole), 7.99 (1H, s, NH-4'); ^{13}C NMR (75 MHz, DMSO-d_6) δ 32.0, 36.9, 44.1, 114.6, 119.6, 121.0, 128.8, 137.63, 143.8, 156.3, 201.7; LCMS (ES$^+$) *m/z* 260.2 [M+H]$^+$; λ_{max} (EtOH) 299.5, 252.0 nm.

1-(4-Aminophenyl)-3-(3-dimethylaminopropyl)urea (S22) [19]

The title compound was prepared using 1-(3-dimethylaminopropyl)-3-(4-nitrophenyl)urea **(S17)** (1.00 g, 3.76 mmol) and 10% palladium on activated carbon (0.30 g) in MeOH (20 mL). Following filtration through Celite and concentration of the filtrate *in vacuo*, the compound was obtained as a red solid after trituration under Et_2O (0.76 g, 85%): mp 122-124 ºC; IR (cm^{-1}) 3304 υ(NH_2), 3177, 2932, 2858, 2818, 2772, 1625 υ(NN'C=O), 1591, 1558, 1510; ^{1}H NMR (300 MHz, DMSO-d_6) δ 1.45-1.59 (2H, quin., *J* = 7.0 Hz, CH_2), 2.14 (6H, s, N(CH_3)$_2$), 2.18-2.29 (2H, t, *J* = 7.0 Hz, CH_2), 2.99-3.10 (2H, dt, *J* = 5.5, 7.0 Hz, CH_2), 4.70 (2H, br s, NH_2), 5.97 (1H, t, *J* = 5.5 Hz, NH), 6.45 (2H, d, *J* = 8.5 Hz, H-2' and H-6'), 6.99 (2H, d, *J* = 8.5 Hz, H-3' and H-5'), 7.92 (1H, br s, NH-4'); ^{13}C NMR (75 MHz, DMSO-d_6) δ 28.2, 38.2, 45.1, 57.8, 114.4, 121.3, 130.0, 144.3, 156.2; LCMS (ES$^+$) *m/z* 237.3 [M+H]$^+$; λ_{max} (EtOH) = 300.0, 252.0 nm.

(Refer to method I from main article)

1-(3-(6-(Cyclohexylmethoxy)-9*H*-purin-2-ylamino)phenyl)-3-(2-morpholinoethyl)urea (S23)

Following Method I, the title compound was prepared using 6-(cyclohexylmethoxy)-2-fluoro-9*H*-purine (0.15 g, 0.59 mmol) and 1-(4-aminophenyl)-3-(3-morpholinoethyl)urea (S19) (0.31 g, 1.17 mmol) and TFA (0.27 mL, 3.5 mmol) in TFE (4 mL). The compound was purified using the Biotage SP4 system (KP-NH; MeOH-EtOAc; 1:9) and the compound was isolated as an off-white solid (87 mg, 30%): mp 145-147 ºC; IR (cm^{-1}) 3294, 2922, 2851, 1660 υ(NN'C=O), 1590, 1538; ^{1}H NMR (300 MHz, DMSO-d_6) δ 1.11 (5H, m, cyclohexyl), 1.73 (6H, s, cyclohexyl), 2.39 (6H, m, N(CH_2)$_3$), 3.17 (2H, m, CH_2), 3.57 (4H, m, OCH_2 morpholine), 3.99 (1H, m, NH), 4.30 (2H, d, *J* = 5.5 Hz, OCH_2), 6.12 (1H, s, NH-4'), 6.91 (1H, d, *J* = 7.5 Hz, H-6'), 7.08 (1H, dd, *J* = 7.5, 7.6 Hz, H-5'), 7.28 (1H, d, *J* = 7.6 Hz, H-4'), 7.86 (1H, s, H-2'), 7.94 (1H, s, H-8), 8.55 (1H, s, NH-4'), 9.14 (1H, s, NH); ^{13}C NMR (125 MHz, DMSO-d_6) δ 25.2, 26.0, 29.2, 35.9, 36.9, 53.2, 57.9, 66.1, 71.1, 108.5, 111.0, 112.0, 114.7, 128.3, 138.8, 140.6, 141.3, 154.2, 155.1, 155.5, 160.1; LCMS (ES$^+$) *m/z* 493.5 [M+H]$^+$; HRMS (ES$^+$) calcd for $C_{25}H_{34}N_8O_3$

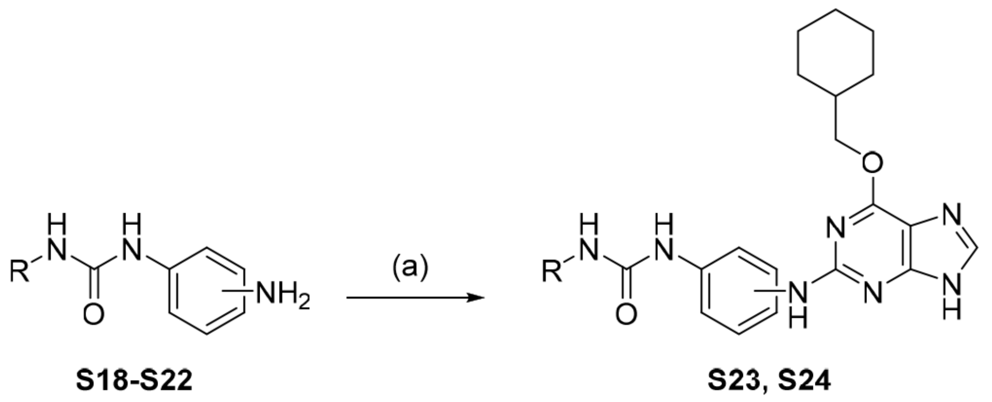

Reagents and conditions: (a) 9, trifluoroacetic acid, 2,2,2,-trifluoroethanol, 90 °C, 18 h.

$[M+H]^+$ 493.2681, found 493.2683; λ_{max} (EtOH) 293.0, 272.0, 231.0 nm.

1-(3-(1*H*-Imidazol-1-yl)propyl)-3-(4-(6-(cyclohexylmethoxy)-9*H*-purin-2-ylamino)phenyl)urea (S24)

Following Method I, the title compound was prepared using 6-(cyclohexylmethoxy)-2-fluoro-9*H*-purine (0.15 g, 0.59 mmol) with 1-(3-(1*H*-imidazol-1-yl)propyl)-3-(4-aminophenyl)urea (S21) (0.30 g, 1.17 mmol) and TFA (0.27 mL, 3.5 mmol) in TFE (4 mL). The compound was purified using the Biotage SP4 chromatography (KP-NH; MeOH-EtOAc; 1:4) to obtain title compound as an off-white solid (87 mg, 30%): mp 177-179 ºC;IR (cm^{-1}) 3105, 2922, 2850, 1621 υ(NN'C=O), 1506; ^{1}H NMR (300 MHz, DMSO-d_6) δ 1.05-1.18 (5H, m, cyclohexyl), 1.69-1.75 (6H, m, cyclohexyl), 1.85 (2H, m, CH_2), 3.04 (2H, dt, *J* = 5.0, 6.0 Hz, CH_2), 3.99 (2H, t, *J* = 7.0 Hz, CH_2), 4.31 (2H, d, *J* = 6.0 Hz, OCH_2), 6.15 (1H, t, *J* = 5.0 Hz, NH), 6.90 (1H, m, CH imidazole), 7.20 (1H, m, CH imidazole), 7.27 (2H, d, *J* = 8.5 Hz, H-2' and H-6'), 7.63 (2H, d, *J* = 8.5 Hz, H-3' and H-5'), 7.93 (1H, m, CH imidazole), 8.27 (1H, s, H-8), 9.10 (1H, s, NH), 12.68 (1H, s, NH-9); ^{13}C NMR (125 MHz, DMSO-d_6) δ 25.2, 26.0, 29.2, 31.5, 36.4, 36.8, 43.7, 70.9, 118.3, 119.2, 119.3, 128.4, 134.1, 135.0, 137.3, 138.5, 154.3, 155.5, 155.7, 160.1; LCMS (ES^+) *m/z* 490.6 $[M+H]^+$; HRMS (ES^+) calcd for $C_{25}H_{32}N_9O_2$ $[M+H]^+$ 490.2673, found 490.2671; λ_{max} (EtOH) 280.5, 238.0, 205.0 nm.

Precursor to trifluoroethylsulfonate ester 26

2,2,2-Trifluoroethyl 3-nitrophenylmethanesulfonate (S25)

To a stirred solution of DMAP (155 mg, 1.3 mmol) and Et_3N (5.37 mL, 38 mmol) in 2,2,2-trifluoroethanol (30 mL) was added 3-nitromethanesulfonyl chloride (3.0 g, 13 mmol). After stirring at room temperature for 3 h the solvent was removed *in vacuo*. The brown residue was extracted into DCM (250 mL) and was washed with 0.05 M HCl (1 × 250 mL) and water (1 × 250 mL). The organic layer was dried through a phase separator and concentrated to give a colourless oil. This was sonicated in water (50 mL) to give the title compound (3.66 g, 96%) as a white crystalline solid that was collected by suction filtration: R_f = 0.52 (petrol-EtOAc; 6:4); mp 84-85 °C, ^{1}H NMR (300 MHz, DMSO-d_6) δ 5.00 (2H, q, *J* = 8.6 Hz, OCH_2CF_3), 5.18 (2H, s, $ArCH_2$), 7.76 (1H, dd, *J* = 7.8, 8.0 Hz, Ar*H*), 7.91 (1H, d, *J* = 7.8 Hz, Ar*H*), 8.29 (1H, d, *J* = 8.2 Hz, Ar*H*), 8.37 (1H, s, Ar*H*); ^{13}C NMR (125 MHz, DMSO-d_6) δ 53.9, 64.9 (q, $^2J_{C\text{-}F}$ = 36 Hz), 122.5 (q, $^1J_{C\text{-}F}$ = 276 Hz), 123.8, 125.5, 130.0, 130.3, 137.4, 147.8; ^{19}F NMR (470 MHz, DMSO-d_6) δ -72.83 (t, *J* = 8.6 Hz, CF_3); Anal. calcd for $C_9H_8F_3NO_5S$: C, 36.13; H, 2.69; N, 4.68%; found: C, 36.19; H, 2.60; N, 4.51%.

2,2,2-Trifluoroethyl 3-aminophenylmethanesulfonate (S26)

The title compound was prepared by stirring 2,2,2-trifluoroethyl 3-nitrophenylmethanesulfonate (**S25**) (3.5 g, 12 mmol) with 10% palladium on carbon (1.1 g) in a mixture of TFE (35 mL) and EtOAc (10 mL) under an atmosphere of H_2 at room temperature for 18 h. The crude reaction product was filtered through Celite, washing with further TFE-EtOAc mixture (3.5:1) and concentrated *in vacuo* to afford a pale yellow powder (2.92 g, 93%): R_f = 0.81 (EtOAc-petrol; 6:4); mp 77-78 °C; ^{1}H NMR (300 MHz, DMSO-d_6) δ 4.71 (2H, s, $ArNH_2$), 4.89 (2H, q, *J* = 8.6 Hz, OCH_2CF_3), 5.22 (2H, s, $ArCH_2$), 6.52-6.62 (3H, m, 3 × Ar*H*), 7.03 (1H, dd, *J* = 7.6, 7.8 Hz, Ar*H*); ^{13}C NMR (125 MHz, DMSO-d_6) δ 55.5, 64.8 (q, $^2J_{C\text{-}F}$ = 36 Hz), 114.2, 115.9, 118.0, 122.6 (q, $^1J_{C\text{-}F}$ = 276 Hz), 128.0, 129.1, 148.9; ^{19}F NMR (470 MHz, DMSO-d_6) δ -72.84 (t, *J* = 8.6 Hz, CF_3); Anal. calcd for $C_9H_{10}F_3NO_3S$: C, 40.15; H, 3.74; N, 5.20%; found: C, 40.24; H, 3.71; N, 5.15%.

Reversed amides; further examples

***N*-(4-(9-Acetyl-6-(cyclohexylmethoxy)-9*H*-purin-2-ylamino)phenyl)acetamide (S27)**

The title compound was obtained using acetyl chloride (0.13 mL, 1.77 mmol) and *N*-1-(6-(cyclohexylmethoxy)-9*H*-purin-2-yl)benzene-1,4-diamine. The crude mixture was purified using chromatography (silica; EtOAc-petrol; 6:4) to obtain the title compound as a beige solid (0.20 g, 79%): mp 200-202 ºC; IR (cm^{-1}) 3257, 2922, 2851, 2259, 2166, 1714 υ(amide C=O), 1660 υ(amide C=O), 1597, 1563, 1503; ^{1}H NMR (300 MHz, DMSO-d_6) δ 1.25 (5H, m, cyclohexyl), 1.78 (6H, m, cyclohexyl), 2.02 (3H, s, CH_3), 2.88 (3H, s, CH_3), 4.34

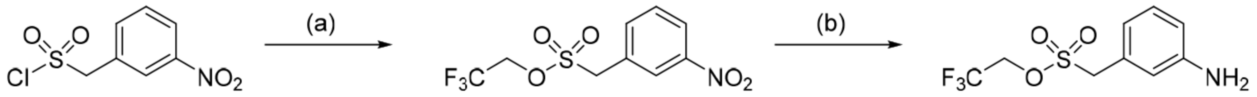

Reagents and conditions: (a) DMAP, Et3N, 2,2,2-trifluoroethanol, RT, 3 h; (b) Pd/C, H_2, MeOH, RT, 18 h,

(2H, d, J = 6.0 Hz, OCH_2), 7.49 (2H, d, J = 9.0 Hz, H-2' and H-6'), 7.66 (2H, d, J = 9.0 Hz, H-3' and H-5'), 8.44 (1H, s, H-8), 9.57 (1H, s, NH), 9.83 (1H, br s, CONH); ^{13}C NMR (125 MHz, DMSO-d_6) δ 7.6, 9.7, 23.9, 25.1, 26.0, 29.2, 36.8, 68.3, 71.3, 118.8, 119.2, 119.4, 119.5, 133.7, 135.5, 137.5, 156.1, 167.8, 168.7; LCMS (ES$^+$) m/z 423.4 [M+H]$^+$; λ_{max} (EtOH) 293.5, 204.5 nm. After acidolysis, the title compound was isolated as yellow solid and was used without further purification (90 mg, 100%): mp 171-173 ºC; IR (cm^{-1}) 2922, 2849, 2360, 2166, 2019, 1640 υ(amide C=O), 1509; ^{1}H NMR (300 MHz, DMSO-d_6) δ 1.1-1.35 (5H, m, cyclohexyl), 1.71-1.91 (6H, m, cyclohexyl), 2.07 (3H, s, CH_3), 4.38 (2H, d, J = 6.0 Hz, OCH_2) 7.51 (2H, d, J = 9.0 Hz, H-2' and H-6'), 7.76 (2H, d, J = 9.0 Hz, H-3' and H-5'), 8.01 (1H, s, H-8), 9.27 (1H, s, NH), 9.85 (1H, s, CONH); ^{13}C NMR (75 MHz, DMSO-d_6) δ 20.7, 25.1, 26.1, 29.2, 36.8, 70.9, 118.7, 119.4, 131.2, 137.5, 155.5, 167.7 (C=O); LCMS (ES$^+$) m/z 381.9 [M+H]$^+$; HRMS (ES$^+$) calcd for $C_{20}H_{24}N_6O_2$ [M+H]$^+$ 380.1961, found 380.1962; λ_{max} (EtOH) 290.0 nm.

N-[4-(6-Cyclohexylmethoxy-9-propionyl-9H-purin-2-ylamino)phenyl]propionamide (S28)

The title compound was obtained from propionyl chloride (0.15 mL, 1.77 mmol) with N-1-(6-(cyclohexylmethoxy)-9H-purin-2-yl)benzene-1,4-diamine(0.20 g, 0.59 mmol), DMAP (70 mg, 0.59 mmol) and triethylamine (0.33 mL, 2.36 mmol). The crude mixture was purified using chromatography (silica; EtOAc-petrol; 6:4) to isolate the title compound as a beige solid (0.20 g, 75%): mp 232-234 ºC; IR (cm^{-1}) 3408, 3300, 3111, 2920, 2852, 1734 υ(amide C=O), 1650 υ(amide C=O), 1587, 1554, 1525; ^{1}H NMR (300 MHz, DMSO-d_6) δ 1.08 (4H, t, J = 7.5 Hz, CH_3), 1.16 (5H, m, cyclohexyl), 1.21 (4H, t, J = 7.03 Hz, CH_3), 1.74 (6H, m, cyclohexyl), 2.30 (1H, m, CH_2), 3.37 (1H, m, CH_2), 4.34 (2H, d, J = 6.0 Hz, OCH_2), 7.52 (2H, d, J = 9.0 Hz, H-2' and H-6'), 7.67 (2H, d, J = 9.0 Hz, H-3' and H-5'), 8.44 (1H, s, H-8), 9.58 (1H, s, NH), 9.76 (1H, s, CONH); ^{13}C NMR (125 MHz, DMSO-d_6) δ 7.9, 9.7, 25.1, 26.0, 29.1, 29.4, 30.2, 36.7, 71.3, 115.7, 119.2, 119.4, 133.7, 135.4, 137.5, 152.3, 156.0, 160.6, 171.5 (C=O), 171.8 (C=O); LCMS (ES$^+$) m/z 451.5 [M+H]$^+$; λ_{max} (EtOH) 315.0, 286.5, 210.5 nm. After acidolysis, the title compound was isolated as beige solid and was used without further purification (88 mg, 100%): mp 193-195ºC; IR (cm^{-1}) 3306, 2920, 2850, 2041, 1976, 1676 υ(amide C=O), 1620, 1587, 1527; ^{1}H NMR (300 MHz, DMSO-d_6) δ 1.08 (3H, t, J = 7.5, Hz, CH_3), 1.20 (5H, m, cyclohexyl), 1.73 (6H, m, cyclohexyl), 2.29 (2H, q, J = 7.5, Hz, CH_2), 4.32 (2H, d, J = 6.0 Hz, OCH_2), 7.47 (2H, d, J = 9.0 Hz, H-2' and H-6'), 7.69 (2H, d, J = 9.0 Hz, H-3' and H-5'), 7.95 (1H, s, H-8), 9.21 (1H, br s, NH), 9.72 (1H, br s, CONH); ^{13}C NMR (75 MHz, DMSO-d_6) δ 9.8, 25.2, 26.0, 29.2, 29.4, 36.8, 70.9, 114.6, 118.8, 119.4, 132.9, 136.4, 138.6, 154.2, 155.5, 160.1, 171.4 (C=O); LCMS (ES$^+$) m/z 395.5 [M+H]$^+$; HRMS (ES$^+$) calcd for $C_{21}H_{26}N_6O_2$ [M+H]$^+$ 394.4710, found 394.4712; λ_{max} (EtOH) 205.5, 234.5, 284.5, 300.5 nm.

N-[4-(6-Cyclohexylmethoxy-9-isobutyryl-9H-purin-2-ylamino)phenyl]isobutyramide (S29)

The title compound was obtained from isobutyryl chloride (0.19 mL, 1.77 mmol) with N-1-(6-(cyclohexylmethoxy)-9H-purin-2-yl)benzene-1,4-diamine (0.20 g, 0.59 mmol), DMAP (70 mg, 0.59 mmol) and triethylamine (0.33 mL, 2.36 mmol). The crude mixture was purified using chromatography (silica; EtOAc-petrol; 6:4) to yield the title compound as a beige solid (0.17 g, 62%): mp 200-202 ºC; IR (cm^{-1}) 3429, 3332, 3136, 2968, 2920, 2849, 1732 υ(amide C=O), 1657 υ(amide C=O), 1583, 1548; ^{1}H NMR (300 MHz, DMSO-d_6) δ 1.10 (6H, d, J = 7.0 Hz, CH_3), 1.16 (5H, m, cyclohexyl), 1.27 (6H, d, J = 7.0 Hz, CH_3), 1.71 (6H, m, cyclohexyl), 2.58 (1H, m, CH), 4.24 (1H, m, CH), 4.34 (1H, d, J = 6.0 Hz, OCH_2), 7.53 (2H, d, J = 9.0 Hz, H-2' and H-6'), 7.65 (2H, d, J = 9.0 Hz, H-3' and H-5'), 8.45 (1H, s, H-8), 9.59 (1H, s, NH), 9.73 (1H, s, CONH); ^{13}C NMR (125 MHz, DMSO-d_6) δ 18.5, 19.5, 25.1, 26.0, 29.1, 33.7, 34.8, 36.7, 71.3, 115.9, 119.3, 119.5, 133.8, 135.4, 137.9, 151.9, 156.2, 160.7, 174.7 (C=O), 174.9 (C=O); LCMS (ES$^+$) m/z 479.6 [M+H]$^+$; λ_{max} (EtOH) 314.5, 270.5, 205.0 nm. After acidolysis, the title compound was isolated and used without further purification (85 mg, 100%): mp 214-216ºC (dec.); IR (cm^{-1}) 3294, 3135, 2923, 2850, 1982, 1632 υ(amide C=O), 1554, 1508; ^{1}H NMR (300 MHz, DMSO-d_6) δ 0.80-0.93 (1H, m, CH), 1.08-1.10 (6H, d, J = 6.0Hz, CH_3), 1.14-1.38 (5H, m, cyclohexyl), 1.58-1.93 (6H, m, cyclohexyl), 4.32 (2H, d, J = 6.0 Hz, OCH_2), 7.48 (2H, d, J = 8.5 Hz, H-2' and H-6'), 7.70 (2H, d, J = 8.5 Hz, H-3' and H-5'), 7.97 (1H, s, H-8), 9.23-9.11 (1H, br s, NH), 9.74-9.62 (1H, br s, CONH); ^{13}C NMR (125 MHz, DMSO-d_6) δ 18.5, 19.6, 25.2, 26.0, 29.2, 34.8, 36.8, 56.0, 70.9, 118.6, 119.5, 132.9, 136.5, 155.4, 156.2, 174.6 (C=O); LCMS (ES$^+$) m/z 409.4 [M+H]$^+$; HRMS (ES$^+$) calcd for $C_{22}H_{28}N_6O_2$ [M+H]$^+$ 409.2347, found 409.2350; λ_{max} (EtOH) 300.5, 285.0, 204.0 nm.

Primary urea; further example

1-(4-(6-(Cyclohexylmethoxy)-9H-purin-2-ylamino)phenyl)urea (S30)

Using N-(6-cyclohexylmethoxy-9H-purin-2-yl)benzene-1,4-diamine the product was obtained as a off-white solid (0.16 g, 72%) was isolated without further purification: mp 187-188 ºC; IR (cm^{-1}) 3279 υ(NH_2), 3129,

2921, 2851, 2361, 1663 υ(NN'C=O), 1619, 1513; ^{1}H NMR (300 MHz, DMSO-d_6) δ 1.05 (5H, m, cyclohexyl), 1.71 (6H, m, cyclohexyl), 4.31 (2H, d, *J* = 6.0 Hz, OCH_2), 5.73 (2H, br s, NH_2), 7.27 (2H, d, *J* = 9.0 Hz, H-2' and H-6'), 7.62 (2H, d, *J* = 9.0 Hz, H-3' and H-5'), 7.97 (1H, s, H-8), 8.33 (1H, br s, CONH), 9.09 (1H, br s, NH-4'), 12.73 (1H, br s, N9-H); ^{13}C NMR (75 MHz, DMSO-d_6) δ 25.5, 26.4, 29.6, 37.2, 71.2, 114.8, 118.6, 119.6, 134.5, 135.4, 138.9, 154.6, 156.1, 156.6, 160.3; LCMS (ES^+) *m/z* 382.0 $[M+H]^+$; HRMS (ES^+) calcd for $C_{19}H_{23}N_7O_2$ $[M+H]^+$ 382.1986, found 382.1985; λ_{max} (EtOH) 280 nm.

Homocarboxamides series; further examples

3-(6-Cyclohexylmethoxy-9*H*-purin-2-ylamino)-*N*-(3-dimethylaminopropyl)benzamide (S31)

The title compound was prepared using acid **23** (80 mg, 0.22 mmol), carbonyldiimidazole (71 mg, 0.44 mmol), DIPEA (79 µL, 0.44 mmol), and *N,N*-dimethyl-1,3-propanediamine (138 µL, 1.1 mmol) in DMF (3 mL). The crude product was purified using a Biotage SP4 purification system (12 + M KP-NH Si cartridge; MeOH-EtOAc; 1:4) to give a colourless powder (65 mg, 63%): R_f = 0.63 (NH_2-modified silica – MeOH-EtOAc; 1:4); mp 128-129 °C; IR (cm^{-1}) 3271, 2922, 2851, 2160, 1583, 1542, 1475, 1438, 1387, 1354, 1306, 1212, 1119, 1038, 975; ^{1}H NMR (300 MHz, DMSO-d_6) δ 1.00-1.90 (13H, m, cyclohexyl and $NCH_2CH_2CH_2NH$), 2.13 (6H, s, 2 × CH_3), 2.26 (2H, t, *J* = 7.0 Hz, Me_2NCH_2), 3.27 (2H overlap with H_2O, m, CH_2CH_2NHCO), 4.35 (2H, d, *J* = 6.1 Hz, OCH_2), 7.29-7.34 (2H, m, 2 × Ar*H*), 7.86 (1H, m, Ar*H*), 8.01 (1H, s, H-8), 8.29 (1H, s, Ar*H*), 8.39 (1H, t, *J* = 5.1 Hz, CONH), 9.41 (1H, s, ArNHAr); ^{13}C NMR (125 MHz, DMSO-d_6) δ 25.2, 26.0, 27.1, 29.2, 36.8, 37.8, 45.2, 57.0, 71.1, 117.8, 119.0, 120.8, 128.1, 135.4, 141.2, 155.3, 166.6; LCMS (ES^+) *m/z* 478.47 $[M+H]^+$; HRMS (ES^+) calcd for $C_{24}H_{34}N_7O_2$ $[M+H]^+$ 452.2768, found 452.2772; λ_{max} (EtOH) 273, 294 nm.

[3-(6-Cyclohexylmethoxy-9*H*-purin-2-ylanilino]-*N, N*-diethylacetamide (S32)

The title compound was prepared using acid **24** (60 mg, 0.16 mmol), carbonyldiimidazole (50 mg, 0.31 mmol), DIPEA (56 µL, 0.31 mmol), and diethylamine (66 µL, 0.63 mmol) in DMF (2 mL). The crude product was purified by chromatography (silica; EtOAc-petrol; 9.5:0.5) to give a white powder (40 mg, 58%): R_f = 0.51 (EtOAc); mp 89-90 °C; IR (cm^{-1}) 2925, 2850, 1587, 1539, 1440, 1390, 1311, 1120; ^{1}H NMR (300 MHz, DMSO-d_6) δ 1.0–1.9 (17H, m, cyclohexyland 2 × CH_2CH_3), 3.26-3.39 (4H, m, 2 × CH_2CH_3), 3.61 (2H, s, $ArCH_2$), 4.29 (2H, d, *J* = 6.2 Hz, OCH_2), 6.80 (1H, d, *J* = 7.7 Hz, Ar*H*), 7.16 (1H, dd, *J* = 7.7, 7.9 Hz, Ar*H*), 7.50–7.54 (2H, m, 2 × Ar*H*), 7.44 (1H, s, H-8), 12.01 (1H, br, N9-H); LCMS (ES^+) *m/z* 437 $[M+H]^+$; HRMS (ES^+) calcd for $C_{24}H_{33}N_6O_2$ $[M+H]^+$ 437.2666, found 437.2656; λ_{max} (EtOH) 213, 272, 292 nm.

3-(6-Cyclohexylmethoxy-9*H*-purin-2-ylamino)phenyl]-1-pyrrolidin-1-ylethanone (S33)

The title compound was prepared using acid **24** (50 mg, 0.13 mmol), carbonyldiimidazole (42 mg, 0.26 mmol), DIPEA (43 µL, 0.26 mmol), and pyrrolidine (43 µL, 0.52 mmol) in DMF (2 mL). The crude product was purified by chromatography (silica; EtOAc) to give a white powder (19 mg, 34%): R_f = 0.20 (EtOAc); mp 114-115 °C; IR (cm^{-1}) 2923, 2849, 2160, 1586, 1539, 1440, 1390, 1350, 1116; ^{1}H NMR (300 MHz, DMSO-d_6) δ 1.0–2.2 (15H, m, cyclohexyl and 2 × pyrrolidine-CH_2), 3.37 (4H, 2 × pyrrolidine-CH_2 overlap with H_2O), 3.52 (2H, s, $ArCH_2$), 4.27 (2H, d, *J* = 6.2 Hz, OCH_2), 6.79 (1H, d, *J* = 7.6 Hz, Ar*H*), 7.12 (1H, dd, *J* = 7.6, 8.1 Hz, Ar*H*), 7.53 (1H, s, Ar*H*), 7.60 (1H, d, *J* = 8.1 Hz, Ar*H*), 8.35 (1H, s, H-8), 12.17 (1H, br, N9-H); LCMS (ES^+) *m/z* 435 $[M+H]^+$; HRMS (ES^+) calcd for $C_{24}H_{31}N_6O_2$ $[M+H]^+$ 435.2503, found 435.2500; λ_{max} (EtOH) 215, 272, 292 nm.

1-(3-(6-Cyclohexylmethoxy-9*H*-purin-2-ylamino) phenylacetyl)azepane (S34)

The title compound was prepared using acid **24** (60 mg, 0.16 mmol), carbonyldiimidazole (50 mg, 0.31 mmol), DIPEA (56 µL, 0.31 mmol), and hexamethyleneimine (71 µL, 0.63 mmol) in DMF (2 mL). The crude product was purified by chromatography (silica; EtOAc-petrol; 9.5:0.5) to give a white powder (34 mg, 47%): R_f = 0.63 (EtOAc); mp 114-115 °C; IR (cm^{-1}) 2921, 2850, 1585, 1538, 1439, 1390, 1352, 1134; ^{1}H NMR (300 MHz, DMSO-d_6) δ 1.0–2.1 (19H, m, cycloalkyl ring CH), 3.42 (4H, m, 2 × RCH_2N), 3.61 (2H, s, $ArCH_2$), 4.28 (2H, d, *J* = 6.2 Hz, CH_2), 6.80 (1H, d, *J* = 7.6 Hz, Ar*H*), 7.14 (1H, dd, *J* = 7.8, 8.0 Hz, Ar*H*), 7.37 (1H, s, Ar*H*), 7.54 (2H, m, 2 × Ar*H*), 8.15 (1H, s, H-8), 12.05 (1H, br, N9-H); LCMS (ES^+) *m/z* 463 $[M+H]^+$; HRMS (ES^+) calcd for $C_{26}H_{35}N_6O_2$ $[M+H]^+$ 463.2816, found 463.2817; λ_{max} (EtOH) 214, 272, 292 nm.

(±)-*N*-(3-Aminocyclohexyl)-2-[3-(6-cyclohexylmethoxy-9*H*-purin-2-ylamino)phenyl]acetamide (S35)

The title compound was prepared using acid **24** (120 mg, 0.32 mmol), carbonyldiimidazole (102 mg, 0.63 mmol), DIPEA (113 µL, 0.63 mmol), and 1,3-diaminocyclohexane (144 mg, 1.26 mmol) in DMF (3 mL). The crude product was purified using a Biotage SP4 purification system (12 + M KP-NH silica cartridge; MeOH-EtOAc; 1:3) to give a colourless powder (95 mg, 63%): R_f = 0.09 (NH_2-modified silica; MeOH-EtOAc; 1:4); mp 188-189 °C; IR (cm^{-1}) 2922, 2850, 1587, 1535, 1489, 1442, 1371, 1312, 1243, 1123, 1043; ^{1}H NMR (300 MHz, DMSO-d_6) δ 0.8–1.9 (19H, m, 2 × cyclohexyl), 3.32 ($ArCH_2$, overlap with H_2O), 3.53 (2H, br m, C*H*NH and C*H*NH_2), 4.34 (2H, d, *J* = 6.0 Hz, OCH_2), 6.80 (1H, d, *J* = 7.4 Hz, Ar*H*), 7.17 (1H, dd, *J* = 7.6, 7.9 Hz, Ar*H*),

7.62 (1H, s, Ar*H*), 7.69 (1H, d, *J* = 8.4 Hz, Ar*H*), 7.95 (1H, br, CONH), 7.98 (1H, s, H-8), 9.21 (1H, s, ArNHAr); LCMS (ES^+) *m/z* 478.47 $[M+H]^+$; HRMS (ES^+) calcd for $C_{26}H_{36}N_7O_2$ $[M+H]^+$ 478.2925, found 478.2925; λ_{max} (EtOH) 272 nm.

2-[3-(6-Cyclohexylmethoxy-9*H*-purin-2-ylamino) phenyl]-*N*-(3-morpholin-4-ylpropyl)acetamide (S36)

The title compound was prepared using acid **24** (75 mg, 0.20 mmol), carbonyldiimidazole (65 mg, 0.40 mmol), DIPEA (72 µL, 0.40 mmol), and 3-morpholinopropylamine (115 mg, 0.80 mmol) in DMF (3 mL). The crude product was purified using a Biotage SP4 purification system (12 + M KP-NH silica cartridge; MeOH-EtOAc; 1:9) to give a colourless powder (82 mg, 81%): R_f = 0.24 (NH_2-modified silica; MeOH-EtOAc; 1:9); mp 127-128 °C; IR (cm^{-1}) 3273, 3103, 3043, 2923, 2851, 1606, 1585, 1559 1538, 1495, 1448, 1394, 1354, 1311, 1217, 1112; ^1H NMR (300 MHz, DMSO-d_6) δ 1.0–1.85 (13H, m, cyclohexyl and $CH_2CH_2CH_2$), 2.20-2.27 (6H, br m, CH_2N and 2 × NCH_2CH_2O), 3.05 (2H, m, CH_2CH_2NHCO), 3.34 (ArCH_2, overlap with H_2O), 3.51 (4H, t, *J* = 4.5 Hz, 2 × NCH_2CH_2O), 4.33 (2H, d, *J* = 6.2 Hz, OCH_2), 6.81 (1H, d, *J* = 7.5 Hz, Ar*H*), 7.17 (1H, dd, *J* = 7.8, 7.8 Hz, Ar*H*), 7.64 (1H, s, Ar*H*), 7.68 (1H, d, *J* = 8.1 Hz, Ar*H*), 7.98 (1H, br t, *J* = 5.4 Hz, CONH), 8.00 (1H, s, H-8), 9.27 (1H, s, ArNHAr), 11.41 (1H, s, N9-H); ^{13}C NMR (125 MHz, DMSO-d_6) δ 25.2, 26.0, 29.2, 36.9, 37.0, 42.8, 53.3, 53.4, 55.8, 66.2, 71.0, 116.6, 119.2, 121.4, 128.1, 136.6, 141.0, 155.4, 169.9; HRMS (ES^+) calcd for $C_{27}H_{38}N_7O_3$ $[M+H]^+$ 508.3031, found 508.3209; λ_{max} (EtOH) 272 nm.

2-[3-(6-Cyclohexylmethoxy-9*H*-purin-2-ylamino) phenyl]-*N*-(2-morpholin-4-ylethyl)acetamide (S37)

The title compound was prepared using acid **24** (75 mg, 0.20 mmol), CDI (65 mg, 0.40 mmol), DIPEA (72 µL, 0.40 mmol), and 4-(2-aminoethyl)morpholine (104 mg, 0.80 mmol) in DMF (3 mL). The crude product was purified using a Biotage SP4 purification system (12 + M KP-NH silica cartridge; MeOH-EtOAc; 1:9) to give an off-white powder (48 mg, 49%): R_f = 0.33 (NH_2-modified silica; MeOH-EtOAc; 1:9); mp 135-137 °C; IR (cm^{-1}) 3276, 2922, 2850, 2161, 1587, 1538, 1492, 1442, 1390, 1352, 1307, 1243, 1212, 1113; ^1H NMR (300 MHz, DMSO-d_6) δ 1.0–1.86 (11H, m, cyclohexyl), 2.33 (6H, br m, 2 × NCH_2CH_2O and CH_2CH_2NHCO), 3.17 (2H, m, CH_2CH_2NHCO), 3.37 (2H, s, ArCH_2), 3.52 (4H, t, *J* = 4.5 Hz, 2 × NCH_2CH_2O), 4.33 (2H, d, *J* = 6.2 Hz, OCH_2), 6.83 (1H, d, *J* = 7.6 Hz, Ar*H*), 7.18 (1H, dd, *J* = 7.8, 7.9 Hz, Ar*H*), 7.65 (1H, s, Ar*H*), 7.69 (1H, d, *J* = 8.3 Hz, Ar*H*), 7.89 (1H, br t, *J* = 5.5 Hz, CONH), 8.00 (1H, s, H-8), 9.27 (1H, s, ArNHAr), 11.90 (1H, s, N9-H); LCMS (ES^+) *m/z* 494.65 $[M+H]^+$; HRMS (ES^+) calcd for $C_{26}H_{36}N_7O_3$ $[M+H]^+$ 494.2874, found 494.2873; λ_{max} (EtOH) 272 nm.

2-[3-(6-Cyclohexylmethoxy-9*H*-purin-2-ylamino) phenyl]-*N*-(3-pyrrolidin-1-ylpropyl)acetamide (S38)

The title compound was prepared using acid **24** (75 mg, 0.20 mmol), carbonyldiimidazole (65 mg, 0.40 mmol), DIPEA (72 µL, 0.40 mmol), and 1-(3-aminopropyl)-pyrrolidine (126 mg, 0.80 mmol) in DMF (3 mL). The crude product was purified using a Biotage SP4 purification system (12 + M KP-NH silica cartridge; MeOH-EtOAc; 1.5:8.5) to give a colourless powder (72 mg, 69%): R_f = 0.14 (NH_2-modified silica; MeOH-EtOAc; 1.5:8.5); mp 133-134 °C; IR (cm^{-1}) 3277, 3209, 3107, 3041, 2922, 2848, 2794, 1642, 1606, 1560, 1496, 1450, 1397, 1356, 1315, 1131, 1242, 1217, 1128; ^1H NMR (300 MHz, DMSO-d_6) δ 1.0–1.85 (17H, m, cyclohexyl, N$CH_2CH_2CH_2$N and 2 × pyrrolidinyl CH_2), 2.31-2.37 (6H, m, NCH_2 and 2 × pyrrolidinyl NCH_2), 3.08 (2H, m, CH_2CH_2NHCO), 3.34 (ArCH_2, overlap with H_2O), 4.33 (2H, d, *J* = 6.2 Hz, OCH_2), 6.80 (1H, d, *J* = 7.6 Hz, Ar*H*), 7.17 (1H, dd, *J* = 7.8, 7.8 Hz, Ar*H*), 7.65 (1H, s, Ar*H*), 7.68 (1H, d, *J* = 7.9 Hz, Ar*H*), 7.97 (1H, br s, CONH), 8.00 (1H, s, H-8), 9.27 (1H, s, ArNHAr), 12.79 (1H, s, N9-H);^{13}C NMR (125 MHz, DMSO-d_6) δ 23.0, 25.2, 26.0, 28.4, 29.2, 36.8, 37.2, 42.8, 53.31, 53.5, 71.0, 116.6, 119.2, 121.4, 128.1, 136.6, 141.0, 155.4, 169.9; LCMS (ES^+) *m/z* 492.67 $[M+H]^+$; HRMS (ES^+) calcd for $C_{27}H_{38}N_7O_2$ $[M+H]^+$ 492.3081, found 492.3076; λ_{max} (EtOH) 272, 292 nm.

***N*-(3-Aminopropyl)-2-[3-(6-cyclohexylmethoxy-9*H*-purin-2-ylamino)phenyl]acetamide (S39)**

The title compound was prepared using acid **24** (60 mg, 0.16 mmol), carbonyldiimidazole (52 mg, 0.32 mmol), DIPEA (57 µL, 0.32 mmol), and 1,3-diaminopropane (53 µL, 0.64 mmol) in DMF (3 mL). The crude product was purified using a Biotage SP4 purification system (12 + M KP-NH silica cartridge; MeOH-EtOAc; 1:3) to give an off-white powder (27 mg, 38%): R_f = 0.10 (NH_2-modified silica; MeOH-EtOAc; 1:3); mp 141-142 °C; IR (cm^{-1}) 3257, 2922, 2850, 2161, 2029, 1585, 1536, 1489, 1438, 1388, 1352, 1306, 1246, 1212, 1163, 1119, 1048, 974; ^1H NMR (300 MHz, DMSO-d_6) δ 1.0–1.85 (11H, m, cyclohexyl), 1.55 (2H, m, $CH_2CH_2CH_2$), 2.56 (2H, t, *J* = 6.7 Hz, $CH_2CH_2NH_2$), 3.11 (2H, m, CH_2CH_2NHCO), 3.35 (2H, s overlap with H_2O, ArCH_2), 3.89 (2H, br, NH_2), 4.33 (2H, d, *J* = 6.2 Hz, OCH_2), 6.79 (1H, d, *J* = 7.4 Hz, Ar*H*), 7.16 (1H, dd, *J* = 7.6, 8.1 Hz, Ar*H*), 7.60 (1H, d, *J* = 8.6 Hz, Ar*H*), 7.73 (1H, s, Ar*H*), 7.96 (1H, s, H-8), 8.03 (1H, t br, *J* = 5.5 Hz, CONH), 9.21 (1H, s, ArNHAr); HRMS (ES^+) calcd for $C_{23}H_{32}N_7O_2$ $[M+H]^+$ 438.2612, found 438.2616; λ_{max} (EtOH) 272 nm.

Homosulfonamides; further examples

4-(3-(6-Cyclohexylmethoxy-9*H*-purin-2-ylamino) phenylmethanesulfonyl)morpholine (S40)

The product from the reaction of 2,2,2-trifluoroethyl-3-(6-cyclohexylmethoxy-9*H*-purin-2-ylamino) phenylmethanesulfonate (**26**) (46 mg, 0.09 mmol),1,8-diazabicyclo[5.4.0]undec-7-ene (42 µl, 0.18 mmol), and morpholine (16 µL, 0.18 mmol), was purified by chromatography (silica; EtOAc-petrol; 8:2) to give the title compound as a white solid (32 mg, 72%): R_f = 0.64 (EtOAc); mp 140-141 °C; IR (cm^{-1}) 3302, 2924, 2854, 1587, 1552, 1494, 1396, 1307, 1242, 1141; ^{1}H NMR (300 MHz, DMSO-d_6) δ 1.0–1.9 (11H, m, cyclohexyl), 3.11 (4H, t, *J* = 4.5 Hz, NCH_2CH$_2$O), 3.57 (4H, t, *J* = 4.5 Hz, NCH$_2$CH_2O), 4.34 (2H, d, *J* = 7.3 Hz, OCH$_2$), 4.35 (2H, s, ArCH$_2$), 6.96 (1H, d, *J* = 7.6 Hz, Ar*H*), 7.27 (1H, dd, *J* = 7.8, 8.1 Hz, Ar*H*), 7.83–7.85 (2H, m, 2 × Ar*H*), 7.98 (1H, s, H-8), 9.43 (1H, s, ArNHAr), 12.77 (1H, br, N9-H); LCMS (ES$^+$) *m/z* 487 [M+H]$^+$; HRMS (ES$^+$) calcd for $C_{23}H_{31}N_6O_4S$ [M+H]$^+$ 487.2122, found 487.2116; λ_{max} (EtOH) 216, 272, 292 nm.

1-(3-(6-Cyclohexylmethoxy-9*H*-purin-2-ylamino) phenylmethanesulfonyl)pyrrolidine (S41)

The product from the reaction of2,2,2-trifluoroethyl-3-(6-cyclohexylmethoxy-9*H*-purin-2-ylamino) phenylmethanesulfonate (**26**) (75 mg, 0.15 mmol),1,8-diazabicyclo[5.4.0]undec-7-ene (68 µL, 0.45 mmol), and pyrrolidine (32 µL, 0.38 mmol), was purified by chromatography (silica; EtOAc-petrol; 8:2) to give the title compound as a white solid (53 mg, 75%): R_f = 0.74 (EtOAc); mp 120-121 °C; IR (cm^{-1}) 2926, 2852, 2159, 2031, 1590, 1540, 1492, 1445, 1394, 1317, 1122; ^{1}H NMR (300 MHz, DMSO-d_6) δ 1.0–1.9 (15H, m, cyclohexyl and 2 × pyrrolidine CH_2), 3.15 (4H, t, *J* = 6.5 Hz, 2 × pyrrolidine CH$_2$), 4.35 (4H, s and d, *J* = 5.8 Hz, OCH$_2$ and ArCH$_2$), 6.96 (1H, d, *J* = 7.5 Hz, Ar*H*), 7.26 (1H, dd, *J* = 7.8, 7.9 Hz, Ar*H*), 7.81 (1H, d, *J* = 8.4 Hz, Ar*H*), 7.89 (1H, s, Ar*H*) 7.98 (1H, s, H-8), 9.41 (1H, s, ArNHAr), 12.78 (1H, br, N9-H); LCMS (ES$^+$) *m/z* 471 [M+H]$^+$; HRMS (ES$^+$) calcd for $C_{23}H_{31}N_6O_3S$ [M+H]$^+$ 471.2173, found 471.2172; λ_{max} (EtOH) 216, 272, 292 nm.

1-[3-(6-Cyclohexylmethoxy-9*H*-purin-2-ylamino) phenyl]-*N*-isopropylmethanesulfonamide (S42)

The product from the reaction of2,2,2-trifluoroethyl-3-(6-cyclohexylmethoxy-9*H*-purin-2-ylamino) phenylmethanesulfonate (**26**) (75 mg, 0.15 mmol),1,8-diazabicyclo[5.4.0]undec-7-ene (68 µL, 0.45 mmol), and isopropylamine (32 µL, 0.38 mmol) was purified by chromatography (12 + M KP-NH silica; EtOAc-MeOH; 9.5:0.5) to give the title compound as a white solid (48 mg, 70%): R_f = 0.37 (NH$_2$-modified silica; EtOAc-MeOH; 9:1); mp 230-231 °C; IR (cm^{-1}) 3435, 3254, 3088, 2928, 2851, 1734, 1598, 1530, 1487, 1447, 1393, 1348, 1300, 1241, 1115, 1026; ^{1}H NMR (300 MHz, DMSO-d_6) δ 1.00-1.90 (11H, m, cyclohexyl), 1.08 (6H, d, *J* = 6.4 Hz, (CH_3)$_2$CH), 3.35 (1H, m, overlap with H$_2$O, C*H*Me$_2$), 4.21 (2H, s, ArCH$_2$), 4.34 (2H, d, *J* = 6.0 Hz, OCH$_2$), 6.92 (1H, d, *J* = 7.3 Hz, Ar*H*), 7.00 (1H, d, *J* = 7.3 Hz, Ar*H*), 7.25 (1H, dd, *J* = 7.8, 7.9 Hz, Ar*H*), 7.83 (1H, s, Ar*H*), 7.84 (1H, s, H-8), 7.98 (1H, br, SO$_2$NH), 9.38 (1H, s, ArNHAr), 12.77 (1H, s br, N9-H); LCMS (ES$^+$) *m/z* 459.42 [M+H]$^+$; HRMS (ES$^+$) calcd for $C_{22}H_{31}N_6O_3S$ [M+H]$^+$ 459.2173, found 459.2172; λ_{max} (EtOH) 272 nm.

1-[3-(6-Cyclohexylmethoxy-9*H*-purin-2-ylamino)phenyl]-*N*-(2-morpholin-4-ylethyl) methanesulfonamide (S43)

The product from the reaction of2,2,2-trifluoroethyl-3-(6-cyclohexylmethoxy-9*H*-purin-2-ylamino) phenylmethanesulfonate (**26**) (60 mg, 0.12 mmol),1,8-diazabicyclo[5.4.0]undec-7-ene (55 µL, 0.36 mmol), and 2-morpholinoethanamine (39 µL, 0.30 mmol) was purified using the Biotage SP4 (12 + M KP-NH silica cartridge; EtOAc-MeOH; 8.5:1.5) to give the title compound as a colourless solid (59 mg, 93%): R_f = 0.23 (NH$_2$-modified silica; EtOAc-MeOH; 9:1); mp 136-137 °C; IR (cm^{-1}) 2924, 2852, 2158, 2021, 1973, 1588, 1542, 1491, 1443, 1393, 1356, 1304, 1243, 1214, 1115; ^{1}H NMR (300 MHz, DMSO-d_6) δ 1.00-1.86 (11H, m, cyclohexyl), 2.34 (6H, br m, 2 × NCH_2CH$_2$O and CH_2CH$_2$SO$_2$NH), 3.03 (2H, t, *J* = 6.6 Hz, CH_2NH), 3.52 (4H, t, *J* = 4.6 Hz, 2 × NCH$_2$CH_2O), 4.31 (2H, s, ArCH$_2$), 4.34 (2H, d, *J* = 6.0 Hz, OCH$_2$), 6.93 (1H, d, *J* = 7.4 Hz, Ar*H*), 7.26 (1H, dd, *J* = 7.6, 7.7 Hz, Ar*H*), 7.81 (1H, s, Ar*H*), 7.83 (1H, d, *J* = 7.7 Hz, Ar*H*), 8.00 (1H, s, H-8), 9.34 (1H, s, ArNHAr); LCMS (ES$^+$) *m/z* 530.43 [M+H]$^+$; HRMS (ES$^+$) calcd for $C_{25}H_{36}N_7O_4S$ [M+H]$^+$ 530.2544, found 530.2549; λ_{max} (EtOH) 272, 293 nm.

1-[3-(6-Cyclohexylmethoxy-9*H*-purin-2-ylamino) phenyl]-*N*-(3-phenylpropyl)methanesulfonamide (S44)

The product from the reaction of 2,2,2-trifluoroethyl-3-(6-cyclohexylmethoxy-9*H*-purin-2-ylamino) phenylmethanesulfonate (**26**) (75 mg, 0.15 mmol),1,8-diazabicyclo[5.4.0]undec-7-ene (68 µL, 0.45 mmol), and 3-phenylpropan-1-amine (53 mg, 0.38 mmol) was purified by chromatography (silica; EtOAc-petrol; 9:1) to give the title compound as a white powder (57 mg, 71%): R_f = 0.41 (EtOAc); mp 131-132 °C; IR (cm^{-1}) 3277, 2923, 2850, 2444, 2361, 1586, 1551, 1510, 1447, 1395, 1352, 1310, 1126, 976; ^{1}H NMR (300 MHz, DMSO-d_6) δ 1.00-1.90 (11H, m, cyclohexyl), 1.70 (2H, m, CH$_2$CH_2CH$_2$NH), 2.56 (2H, t, *J* = 7.7 Hz, PhCH$_2$), 2.92 (2H, m, CH$_2$CH_2NH), 4.24 (2H, s, ArCH$_2$), 4.35 (2H, d, *J* = 6.0 Hz, OCH$_2$), 6.91 (1H, d, *J* = 7.5 Hz, Ar*H*), 7.10-7.28 (7H, m, 7 × Ar*H*), 7.80-7.85 (2H, m, Ar*H* and SO$_2$N*H*), 8.01 (1H, s, H-8), 9.36 (1H, s, ArNHAr), 12.69 (1H, s br, N9-H); ^{13}C NMR

(125 MHz, CD_3OD) δ 25.6, 26.3, 29.6, 32.0, 32.5, 37.5, 42.8, 58.3, 71.8, 118.6, 120.9, 123.6, 124.8, 125.5, 128.0, 128.0, 128.4, 137.8, 141.2, 141.5, 156.3; LCMS (ES^+) *m/z* 535.45 $[M+H]^+$; HRMS (ES^-) calcd for $C_{28}H_{33}N_6O_3S$ $[M-H]^-$ 533.2340, found 533.2350; λ_{max} (EtOH) 272 nm.

1-[3-(6-Cyclohexylmethoxy-9*H*-purin-2-ylamino) phenyl]-*N*-(4-methylbenzyl)methanesulfonamide (S45)

The product from the reaction of 2,2,2-trifluoroethyl-3-(6-cyclohexylmethoxy-9*H*-purin-2-ylamino) phenylmethanesulfonate (**26**) (75 mg, 0.15 mmol),1,8-diazabicyclo[5.4.0]undec-7-ene (68 µL, 0.45 mmol), and *p*-tolylmethanamine (48 µL, 0.38 mmol) was purified by chromatography (silica; EtOAc-petrol; 3:1) to give the title compound as a white solid (56 mg, 72%): R_f = 0.46 (EtOAc-petrol; 1:4); mp 147-148 °C; IR (cm^{-1}) 2922, 2850, 2361, 2338, 1589, 1540, 1491, 1443, 1393, 1352, 1306, 1121, 1059; ^{1}H NMR (300 MHz, DMSO-d_6) δ 1.00-1.86 (11H, m, cyclohexyl), 2.26 (3H, s, $ArCH_3$), 4.07 (2H, s, $ArCH_2NH$), 4.23 (2H, s, $ArCH_2SO_2NH$), 4.35 (2H, d, *J* = 6.1 Hz, OCH_2), 6.90 (1H, d, *J* = 7.5 Hz, Ar*H*), 7.12 (2H, d, *J* = 7.8 Hz, 2 × Ar*H*), 7.20 (2H, d, *J* = 7.9 Hz, 2 × Ar*H*), 7.26 (1H, dd, *J* = 7.8, 7.8 Hz, Ar*H*), 7.81-7.85 (2H, m, 2 × Ar*H*), 8.01 (1H, s, H-8), 9.35 (1H, s, ArNHAr), 12.79 (1H, s br, N9-H); LCMS (ES^+) *m/z* 521.39 $[M+H]^+$; HRMS (ES^+) calcd for $C_{27}H_{33}N_6O_3S$ $[M+H]^+$ 521.2329, found 521.2328; Anal. calcd for $C_{27}H_{32}N_6O_3S$: C, 62.29; H, 6.19; N, 16.15%; found: C, 62.18; H, 6.24; N, 16.04%; λ_{max} (EtOH) 272 nm.

1-[3-(6-Cyclohexylmethoxy-9*H*-purin-2-ylamino) phenyl]-*N*-(4-methoxyphenyl)methanesulfonamide (S46)

The product from the reaction of 2,2,2-trifluoroethyl-3-(6-cyclohexylmethoxy-9*H*-purin-2-ylamino) phenylmethanesulfonate (**26**) (75 mg, 0.15 mmol),1,8-diazabicyclo[5.4.0]undec-7-ene (68 µL, 0.45 mmol), and 4-aminoanisole (45 mg, 0.38 mmol) was purified by chromatography (silica; EtOAc-petrol; 4:1) to give the title compound (45 mg, 57%): R_f = 0.43 (EtOAc-petrol; 4:1); mp 220-221 °C; IR (cm^{-1}) 3341, 2924, 2850, 2361, 2337, 1632, 1608, 1579, 1553, 1499, 1438, 1404, 1362, 1331, 1296, 1220, 1150, 1120, 1030; ^{1}H NMR (300 MHz, DMSO-d_6) δ 1.00-1.83 (11H, m, cyclohexyl), 3.73 (3H, s, $ArOCH_3$), 4.24 (2H, s, $ArCH_2SO_2NH$), 4.27 (2H, d, *J* = 6.2 Hz, OCH_2), 6.80 (1H, d, *J* = 7.5 Hz, Ar*H*), 6.89 (2H, d, *J* = 8.9 Hz, 2 × Ar*H*), 7.16 (2H, d, *J* = 8.9 Hz, 2 × Ar*H*), 7.25 (1H, dd, *J* = 7.9, 7.9 Hz, Ar*H*), 7.75-7.82 (2H, m, 2 × Ar*H*), 8.01 (1H, s, H-8), 9.39 (1H, s, ArNHAr), 12.79 (1H, s br, N9-H); LCMS (ES^+) *m/z* 523.40 $[M+H]^+$; HRMS (ES^+) calcd for $C_{26}H_{31}N_6O_4S$ $[M+H]^+$ 523.2122, found 523.2122; λ_{max} (EtOH) 273 nm.

***N*-(4-*tert*-Butylphenyl)-1-[3-(6-cyclohexylmethoxy-9*H*-purin-2-ylamino)phenyl]methanesulfonamide (S47)**

The product from the reaction of2,2,2-trifluoroethyl-3-(6-cyclohexylmethoxy-9*H*-purin-2-ylamino) phenylmethanesulfonate (**26**) (75 mg, 0.15 mmol),1,8-diazabicyclo[5.4.0]undec-7-ene (68 µL, 0.45 mmol), and 4-*t*-butylaniline (60 µL, 0.38 mmol) was purified by chromatography (silica; EtOAc-petrol; 3:1) to give the title compound as a colourless powder (39 mg, 47%): R_f = 0.54 (EtOAc-petrol; 4:1); mp 132-133 °C; IR (cm^{-1}) 3356, 2923, 2851, 2361, 2338, 1707, 1591, 1499, 1442, 1398, 1356, 1300, 1147, 1125; ^{1}H NMR (300 MHz, DMSO-d_6) δ 1.00-1.83 (11H, m, cyclohexyl), 1.25 (9H, br s, $C(CH_3)_3$), 4.29 (2H, d, *J* = 6.2 Hz, OCH_2), 4.31 (2H, s, $ArCH_2SO_2NH$), 6.79 (1H, d, *J* = 7.1 Hz, Ar*H*), 7.14 (2H, d, *J* = 8.6 Hz, 2 × Ar*H*), 7.24 (1H, dd, *J* = 7.7, 8.0 Hz, Ar*H*), 7.32 (2H, d, *J* = 8.5 Hz, 2 × Ar*H*), 7.76 (1H, s, Ar*H*), 7.81 (1H, d, *J* = 7.9 Hz, Ar*H*), 8.00 (1H, s, H-8), 9.37 (1H, s, ArNHAr), 12.79 (1H, s br, N9-H); LCMS (ES^+) *m/z* 549.49 $[M+H]^+$; HRMS (ES^+) calcd for $C_{29}H_{37}N_6O_3S$ $[M+H]^+$ 549.2642, found 549.2647; λ_{max} (EtOH) 273 nm.

Precursor for 6-alkoxy derivatives compounds 59 and 60

2-(3-Nitrophenyl)-*N*-(3-dimethylaminopropyl) acetamide (S48)

To a stirred solution of 3-nitrophenylacetic acid (2.0 g, 11 mmol) in THF (15 mL) was added $SOCl_2$ (0.88 mL, 12 mmol) and the reaction mixture was stirred at room temperature for 2 h. Solvents were removed *in vacuo* and the residual oil was redissolved in THF (15 mL). *N,N*-dimethyl-1,3-propanediamine (6.9 mL, 55 mmol) was added dropwise over 15 min and the reaction mixture was stirred for an additional 18 h. The mixture was concentrated to give a viscous oil that was extracted

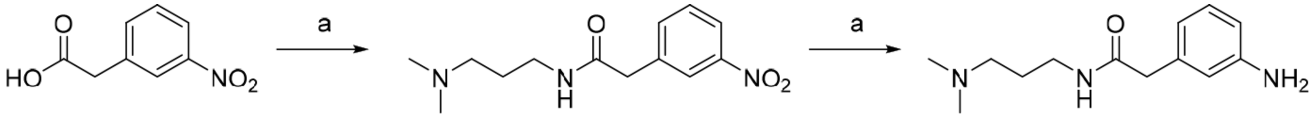

119 120 120

Reagents and conditions: (i) $SOCl_2$, THF, RT, 2 h, (ii) $(CH_3)_2N(CH_2)_3NH_2$,RT, 18 h, 72%; (b) Pd/C, H_2, EtOAc/MeOH, RT, 18 h, 99%;

into EtOAc (200 mL) and washed with aqueous $NaHCO_3$ solution (250 mL). The aqueous layer was reextracted with EtOAc (200 mL) and the organic layers were combined, dried (Na_2SO_4) and concentrated to give the title compound as a brown oil (2.1 g, 72%): ^{1}H NMR (300 MHz, $CDCl_3$) δ 1.58 (2H, m, $CH_2CH_2CH_2$), 2.02 (6H, s, $N(CH_3)_2$), 2.31 (2H, t, J = 5.9 Hz, $CH_2N(CH_3)_2$), 3.33 (2H, m CH_2NHCO), 3.61 (2H, s, $ArCH_2$), 7.50-7.57 (1H, m, Ar*H*), 7.64 (1H, d, J = 7.8 Hz, Ar*H*), 8.15-8.18 (2H, m, 2 × Ar*H*).

2-(3-Aminophenyl)-*N*-(3-dimethylaminopropyl) acetamide (S49)

2-(3-Nitrophenyl)-*N*-(3-dimethylaminopropyl) acetamide (**S48**) (2.0 g, 7.6 mmol) was treated with 10% palladium on carbon (250 mg) in EtOAc-MeOH (1:1; 25 mL) under H_2 and stirred at room temperature for 18 h. The crude suspension was filtered through a plug of Celite, washing with EtOAc-MeOH (1:1) and concentrated to give the title compound as a brown viscous oil (1.76 g, 99%): ^{1}H NMR (300 MHz, DMSO-d_6) δ 1.44-1.56 (2H, m, $CH_2CH_2CH_2$), 2.07 (6H, s, $N(CH_3)_2$), 2.16 (2H, d, J = 7.1 Hz, $CH_2N(CH_3)_2$), 3.04 (2H, m, CH_2NHCO), 3.20 (2H, s, $ArCH_2$), 4.99 (2H, br s, $ArNH_2$), 6.35-6.45 (1H, m, Ar*H*), 6.60-6.69 (1H, m, Ar*H*), 6.74 (1H, s, Ar*H*), 7.06 (1H, dd, J = 7.7, 7.7 Hz, Ar*H*), 7.92 (1H, t, J = 5.4 Hz, CONH).

Purine-6-enamine; further examples

(*E*)-6-(2-(4-Methylpiperazin-1-yl)vinyl)-*N*-phenyl-9*H*-purin-2-amine (S50)

The title compound was prepared by reaction 1-methylpiperazine (472 µL, 4.23 mmol). Yellow solid (45 mg, 64%): mp 137-139 °C; IR (cm^{-1}) 2928, 2832, 2794, 1631, 1564; ^{1}H NMR (500 MHz, DMSO-d_6) δ 2.33 (1H, s, CH_3), 2.51 (4H, m, CH_2), 3.42 (4H, m, CH_2), 5.68-5.71 (1H, d, J = 15.0 Hz, alkene CH), 6.93-6.97 (1H, t, J = 10.0 Hz, H-4'), 7.27-7.31 (2H, dd, J = 9.8, 10.0 Hz, H-3' and H-5'), 7.92-7.94 (2H, d, J = 9.8 Hz, H-2' and H-6'), 8.04 (1H, s, H-8), 8.31-8.33 (1H, d, J = 15.0 Hz, alkene CH), 9.09 (1H, s, NH), 12.67 (1H, br s, NH-9); ^{13}C NMR (125 MHz, DMSO-d_6) δ 45.7, 54.0, 92.1, 117.9, 119.9, 122.3, 128.3, 138.5, 141.8, 148.3, 151.6, 156.0, 156.5; LCMS (ES$^+$) m/z 336.3 [M+H]$^+$; HRMS (ES$^+$) calcd for $C_{18}H_{21}N_7$ [M+H]$^+$ 336.1934, found 336.1931; λ_{max} (EtOH) 357.5, 331.0, 280.0, 253.5 nm.

(*E*)-6-(2-Morpholinovinyl)-*N*-phenyl-9*H*-purin-2-amine (S51)

The title compound was prepared by reaction of morpholine (371 µL, 4.23 mmol). Yellow solid (51 mg, 75%): mp 230-232 °C; IR (cm^{-1}) 3298, 2960, 2923, 2845, 1634, 1603, 1576, 1525; ^{1}H NMR (500 MHz, DMSO-d_6) δ 3.51 (4H, m, CH_2), 3.88 (4H, m, CH_2), 5.80-5.83 (1H, d, J = 15.0 Hz, alkene CH), 7.02-7.06 (1H, t, J = 10.0 Hz, H-4'), 7.41-7.45 (2H, dd, J = 9.8, 10.0 Hz, H-3' and H-5'), 8.01-8.03 (2H, d, J = 9.8 Hz, H-2' and H-6'), 8.15 (1H, s, H-8), 8.40-8.43 (1H, d, J = 15 Hz, alkene CH), 9.21 (1H, br s, NH), 12.79 (1H, br s, NH-9); ^{13}C NMR (125 MHz, DMSO-d_6) δ 65.7, 92.4, 117.9, 119.9, 122.4, 128.3, 138.6, 141.8, 148.6, 151.7, 156.0, 156.3; LCMS (ES$^+$) m/z 323.3 [M+H]$^+$; HRMS (ES$^+$) calcd for $C_{17}H_{18}N_6O$ [M+H]$^+$ 323.1615, found 323.1615; λ_{max} (EtOH) 356.5, 330.5, 279.0, 235.5 nm.

(*S, E*)-6-(2-(3-(Dimethylamino)pyrrolidin-1-yl)vinyl)-*N*-phenyl-9*H*-purin-2-amine (S52)

The title compound was prepared by reaction of (*S*)-3-dimethylaminopyrrolidine (540 µL, 4.23 mmol). Yellow solid (49 mg, 67%): mp 136-138 °C; IR (cm^{-1}) 2948, 2779, 2121, 1630, 1561, 1528; ^{1}H NMR (500 MHz, $CDCl_3$) δ 1.76-1.84 (1H, m, pyrrolidine H-3"), 2.08 (1H, m, Pyrrolidine CH), 2.20 (6H, s, $N(CH_3)_2$), 2.70 (1H, m, Pyrollidine CH), 3.49-3.50 (4H, m, Pyrrolidine CH_2), 5.40 (1H, d, J = 15.0 Hz, alkene CH), 6.79-7.00 (1H, t, J = 8.0 Hz, H-4'), 7.23-7.26 (2H, dd, J = 7.5, 8.0 Hz, H-3' and H-5'), 7.45-7.46 (2H, d, J = 7.5 Hz, H-2' and H-6'), 8.31 (1H, d, J = 15.0 Hz, alkene CH); ^{13}C NMR (125 MHz, DMSO-d_6) δ 13.9, 29.7, 30.2, 44.3, 65.2, 91.7, 121.1, 122.7, 123.1, 129.3, 137.7, 140.01, 146.2, 151.2, 155.9, 157.7; LCMS (ES$^+$) m/z 350.3 [M+H]$^+$; HRMS (ES$^+$) calcd for $C_{19}H_{23}N_7$ [M+H]$^+$ 350.2085, found 350.2088; λ_{max} (EtOH) 263.0, 282.0, 361.0 nm.

(*E*)-6-(2-(Methyl(2-(pyridin-2-yl)ethyl)amino)vinyl)-*N*-phenyl-9*H*-purin-2-amine (S53)

The title compound was prepared by reaction of *N*-methyl-2-(pyridin-2-yl)ethanamine (590 µL, 4.23 mmol). Yellow solid (72 mg, 92%): mp 88-90 °C; IR (cm^{-1}) 2330, 2118, 1632, 1564; ^{1}H NMR (500 MHz, $CDCl_3$) δ 2.80 (3H, s, CH_3), 3.00 (2H, t, J = 10.0 Hz, CH_2), 3.64 (2H, t, J = 10.0 Hz, CH_2), 5.50 (1H, m, alkene CH), 6.72 (1H, br s, H-8), 6.99 (1H, t, J = 8.0 Hz, H-4'), 7.07 (2H, m, Py-H), 7.25 (2H, dd, J = 7.5, 8.0 Hz, H-3' and H-5'), 7.46 (2H, d, J = 7.5, H-2' and H-6'), 7.49-7.53 (1H, m, Py-H), 8.19 (1H, d, J = 15.0 Hz, alkene CH), 8.48 (1H, d, J = 5.0 Hz, Py-H), 12.59 (1H, br s, NH-9); ^{13}C NMR (125 MHz, DMSO-d_6) 55.0, 56.8, 68.3, 90.8, 117.9, 119.8, 121.8, 122.0, 123.5, 128.2, 136.6, 138.1, 141.9, 148.7, 149.2, 151.5, 155.9, 156.6, 158.5; LCMS (ES$^+$) m/z 372.3 [M+H]$^+$; HRMS (ES$^+$) calcd for $C_{21}H_{21}N_7$ [M+H]$^+$ 372.1936, found 372.1931; λ_{max} (EtOH) 245.5, 280.0, 361.0 nm.

Supplementary Table 1: Isolated yields from reaction of 6-ethynylpurine 68 with secondary amines

Product	Amine	Isolated yield (%)
**S50**	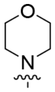	64
**S51**	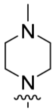	75
**69**	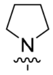	93
**70**	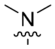	63
**71**	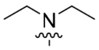	60
**72**	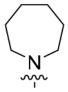	98
**73**	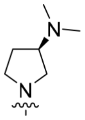	67
**S52**	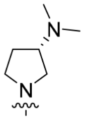	67
**S53**	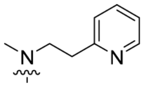	93

Synthesis of enamine derivatives – isolated yields.

Supplementary Table 2: Inhibition of CDK2 and Nek2 by 2-arylamino-*O*[6]-cyclohexylmethylpurines. Complete Nek2/CDK2 inhibitory data for synthesised inhibitors. Complete data including compounds referred to, but not shown in the main article. Note: compounds designated 'S' are shown in electronic supplementary information only.

See Supplementary File 1

Supplementary Table 3: Nek2 and CDK2 inhibition by selected 6-unsubstituted and 6-substituted purines

See Supplementary File 1

Supplementary Table 4: The effect of pre-incubation (2-60 min) of Nek2 with 70 on IC_{50} values

	Time after inhibitor addition					
	**2 min**	**5 min**	**10 min**	**15 min**	**30 min**	**60 min**
Hill slope	0.73	0.59	0.79	0.71	0.72	0.85
IC_{50} (μM)	1.37	0.84	1.58	1.33	1.06	1.47

Supplementary Table 5: Summary of crystallographic analyses

	8	11	6	71
**Crystals**				
Spacegroup	$P2_12_12_1$	$P2_12_12_1$	$P2_12_12_1$	$C2$
Lattice constants *a* (Å)	56.72	56.42	56.73	100.30
b (Å)	73.74	73.49	73.59	57.61
c (Å)	75.08	74.44	75.39	79.92
β (°)	90.00	90.00	90.00	132.99
**Data collection**				
Resolution range (Å)	73.7-1.9	73.5-1.9	73.5-2.3	50.0-2.4
(Highest resolution shell)	(2.0-1.9)	(2.0-1.9)	(2.4-2.3)	(2.5-2.4)
Unique reflections	25458 (3533)	24327 (3035)	14309 (4867)	13101 (1886)
Completeness(%)	99.8 (99.7)	97.4 (86.3)	98.3 (90.6)	99.5 (99.4)
Multiplicity	4.6 (3.5)	3.4 (3.0)	3.5 (2.6)	3.4 (3.5)
Rmerge (%)	7.5 (34.6)	6.1 (38.2)	6.3 (30.2)	16.7 (58.6)
I/σ(I)	15.8 (3.2)	13.2 (3.0)	13.6 (3.1)	4.2 (2.0)
**Refinement**				
Resolution range (Å)	52.63-1.90	36.75-1.90	36.68-2.40	36.68-2.40
*R*factor (%)	22.20	19.60	23.19	18.56
*R*free[a] (%)	25.67	23.52	28.06	24.49
RMS (bonds)	0.008	0.008	0.010	0.007
RMS (angles)	1.057	1.078	1.312	1.031
**Molprobity analysis**				
Clashscore	3.5	4.9	8.61	4.4
Ramachandran favoured (%)	94.9	96.83	91.4	97.0
Ramachandran outliers (%)	0.40	0.40	2.7	0.40

[a] Free *R*factor was computed using 5% of the data assigned randomly.
